# The path to an open-shell metallo-germylene: direct ligation, or reduction and metathesis?

**DOI:** 10.1039/d5sc04265h

**Published:** 2025-09-16

**Authors:** Annika Schulz, Myron Heinz, Max. C. Holthausen, Terrance J. Hadlington

**Affiliations:** a Fakultät für Chemie, Technische Universität München Lichtenberg Strasse 4 85747 Garching Germany terrance.hadlington@tum.de; b Institut für Anorganische und Analytische Chemie, Goethe-Universität Frankfurt Max-von-Laue-Strass 7 60438 Frankfurt Germany max.holthausen@chemie.uni-frankfurt.de

## Abstract

Reaction of chelating cationic germylene ligand [^PhiP^DipGe]^+^ (1; ^PhiP^Dip = {[Ph_2_PCH_2_Si(^i^Pr)_2_](Dip)N}; Dip = 2,6-^i^Pr_2_C_6_H_3_) with the NHC-stabilised Co^0^ system [IPr·Co(η_2_-vtms)_2_] (IPr = [(H)CN(Dip)C:]; vtms = C_2_H_3_(SiMe_3_) gives ready access to the first example of an open-shell metallo-germylene in high yields, in T-shaped Co complex 2. The Co centre in 2 is found to have a low-spin d^7^ electronic structure which bears a high-spin density of the single unpaired electron in this complex, corroborated by SQUID magnetometry, EPR spectroscopy, and quantum-chemical calculations. Detailed analysis of the electronic structure of 2 establishes the electron-sharing covalent nature of the germanium cobalt interaction. Still, the pathway to 2 is not trivial: at first glance, it seems as though complex 2 is formed *via* a simple insertion of Co^0^ into the P–Ge bond in 1. However, modifying reaction conditions leads to the isolation of fragments of complex 2 (*viz*. 3, 4, and 5), all of which are fully characterised. It is ultimately found that these arise from the initial formation of dimeric germanium(i) species 7, formed by reduction of 1 by Co^0^. Depending on stoichiometry, 7 reacts with intermediary Co^I^ species forming fragments 3–5, or the target cobalto-germylene 2. These results thus demonstrated that 2 is in fact formed *via* the homolytic metathesis of a Ge^I^–Ge^I^ bond at Co^I^, so opening an unprecedented route to such metallo-tetrylenes.

## Abstract 2

Reaction of a cationic germylene with Co^0^ forms a unique open-shell metallotetrylene. This may seem a direct ligand addition, but we find a deeper mechanism – an initial reductive formation of a Ge^I^ dimer, followed by its homolytic cleavage by Co^I^

## Graphical abstract

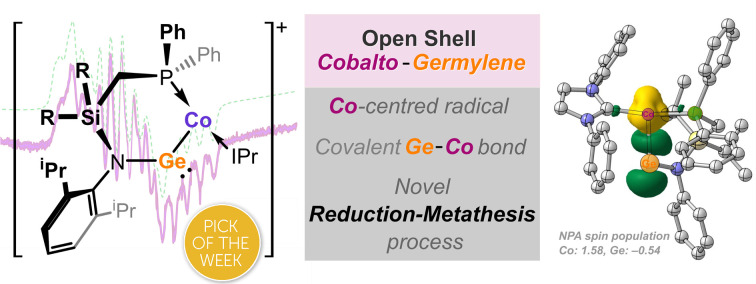

## Introduction

**Fig. 1:**
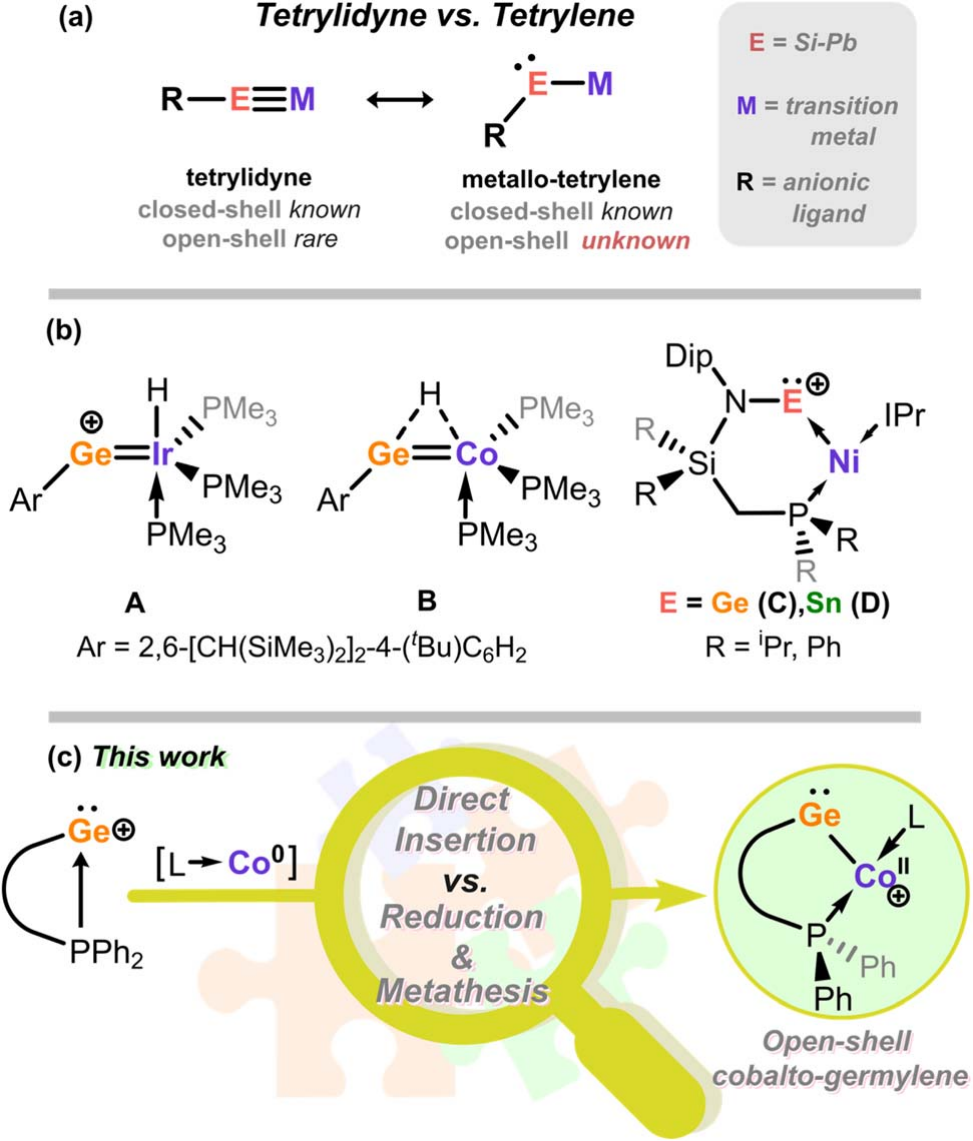
(a) Classical isomers for neutral tetrylidyne species; (b) reported systems closest in electronic nature to group 9 metallo-tetrylenes (A and B), and geometric strain leading to Z-type tetrylene complexes (C and D); (c) this work. L = NHC ligand.

The nature of the bonding between low-valent heavier group 14 elements and d-block metals has long been of interest,^1–6^ particularly in observing both trends and differences with well described carbon chemistry.^1,7–10^ This has often focused on the formation of multiple TM-E bonds (TM = transition metal; E = Si–Pb),^1,2^ given that elements E are more reluctant to partake in multiple bonding relative to C,^11–13^ leading to the isolation of a number of tetrylidyne species bearing formal TM-E triple bonds, which can be directly compared with the well-established carbon congeners, *i.e.* alkylidynes. As for the latter, heavier tetrylidyne derivatives typically bear a linear TM-E-R geometry (Fig. 1(a)).^14–17^ These demonstrate exemplary 1,2-addition and [2 + 2] cycloaddition chemistry,^18–20^ again aligning with carbon congeners. At the other bonding extreme, singly-bonded metallo-tetrylenes can be formed with a bent TM-E-R geometry (*viz.*Fig. 1(a)),^15,21–23^ most often due to electronic saturation of the TM centre, *e.g.* with donor ligands. Closed-shell examples of metallo-germylenes are known for a handful of TMs, namely Cr^II^/Mo^II^/W^II^,^15,19,24–26^ Fe^II^,^27,28^ Pt^II^,^29^ and Zn^II^.^30^ The closest such species to group 9 metallo-tetrylenes are those recently reported by Wesemann *et al.*, *viz*. A and B (Fig. 1(b)),^20,31^ which bear formal multiple Ge-M bonds (M = Ir, Co), and either a cationic Ge centre (A) or a [Ge–H–Co] bridging hydride ligand (B). Whilst these are certainly highly interesting complexes, they cannot be unambiguously described as metallo-tetrylenes, *i.e.* a divalent tetryl centre bound by at least one metallo-ligand. Notably, singlet groundstate metallo-carbene derivatives were discovered as recently as 2022,^32–34^ and triplet derivatives only in 2024.^35^ Though a very small number of open-shell tetrylidyne species are known,^36^ to the best of our knowledge no open-shell metallo-tetrylenes have been reported for Si–Pb, therefore representing an unexplored space in reactive p-block-TM complexation. In order to divulge the chemistry and electronic nature of such species, then, new synthetic protocols should be explored.

Both tetrylidyne and metallo-tetrylene compound classes typically bear a covalent TM-E bond, and for tetrylidyne species additional dative E → TM bonding and concomitant back-bonding.^2^ We have recently demonstrated that cationic tetrylenes with a low coordination number, in conjunction with enforced geometric constraints through chelation, leads to the formation of rare T-shaped Ni^0^ systems in which the cationic tetrylene ligand switches from an L-type to a Z-type ligand, now accepting electron density from Ni^0^ (C and D, Fig. 1(b)).^37^ A similar phenomenon has also been observed in both neutral and cationic silylene-Ni systems reported by Kato *et al.*,^38–40^ as well as in amidinato–tetrylene complexes.^41,42^ We aimed to explore similar methodologies utilizing an open-shell TM synthon, ultimately targeting open-shell tetrylene complexes which cannot form multiple E-TM bonds, and potentially giving access to the novel compound class of open-shell metallo-tetrylenes by formal addition reactions at TM.

Herein we describe utilizing this strategy in low-valent cobalt chemistry, in which the formal insertion of Co^0^ into the P–Ge bond in 1 generates an unprecedented open-shell metallo-germylene featuring a 3-coordinate T-shaped Co^II^ centre, with a low-spin d^7^ electronic configuration (Fig. 1(c)). Although this at first appears as a simple addition of the cationic germylene to cobalt, numerous fragments of the target complex, arising largely from ligand P–C activation and reductive coupling processes, are isolated when reaction times are shortened, signifying a more complex mechanism. This ultimately leads to the finding that an initial reduction pathway proceeds, forming a digermyne congener, which then undergoes oxidative metathesis of the Ge–Ge bond at Co^I^ in forming the final cobalto-germylene. The unique electronic nature of this central species is uncovered through EPR spectroscopy, SQUID magnetometry, and in-depth computational analyses, marking an important new entry into the coordination chemistry of group 14 and late 3d-metals.

## Results and discussion

### Synthesis and characterisation of an open-shell cobalto-germylene

**Scheme 1:**
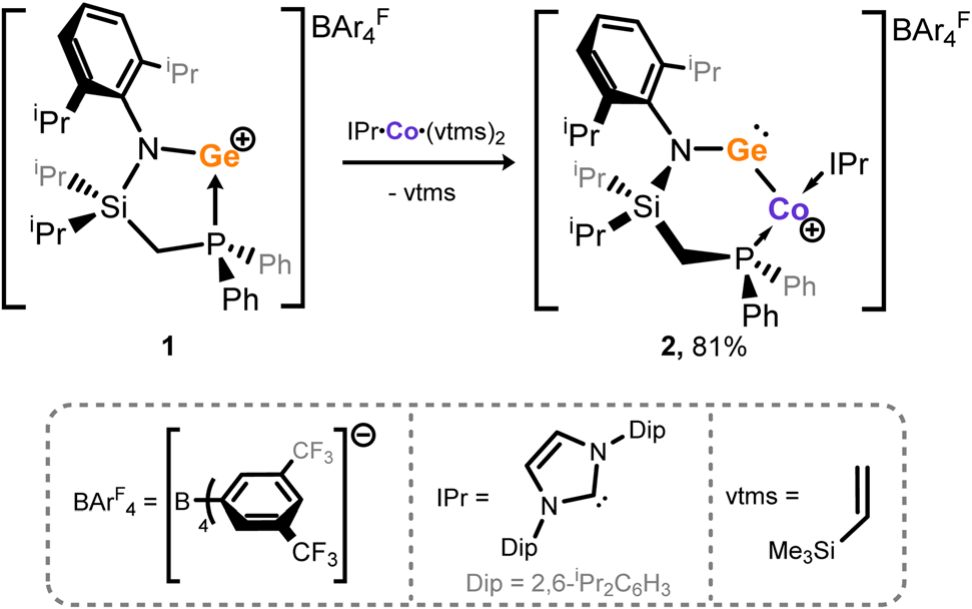
Synthesis of cobalto-germylene complex 2.

**Fig. 2:**
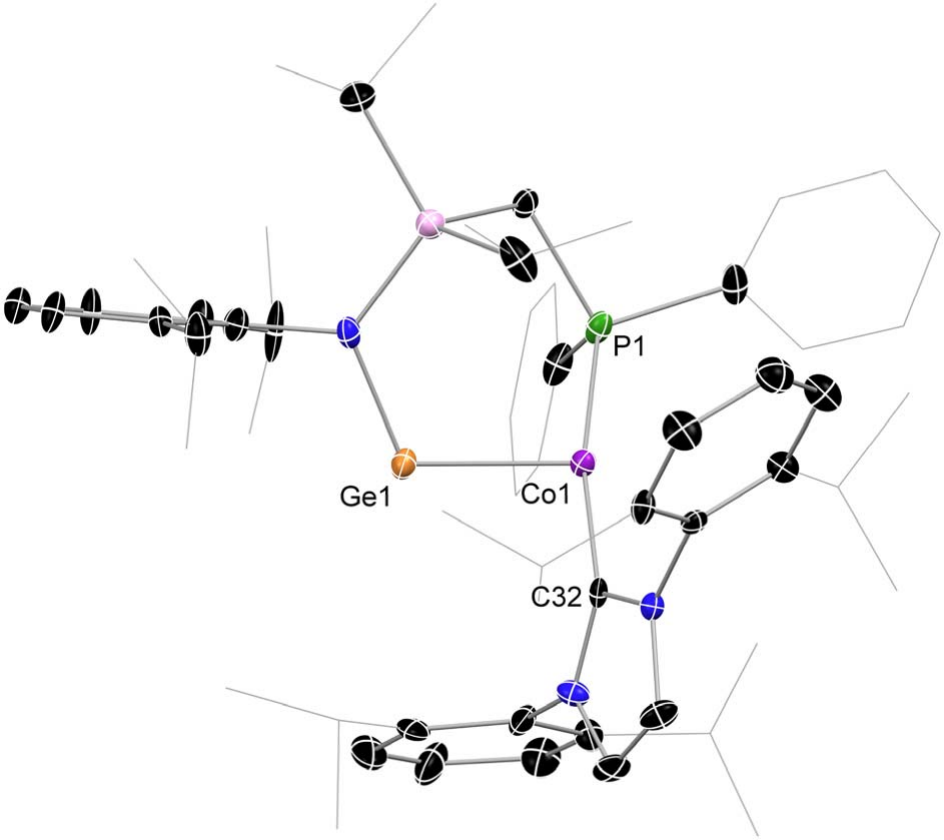
The molecular structure of the cationic part in 2, with ellipsoids at 30% probability, and hydrogen atoms omitted for clarity. Selected bond lengths (Å) and angles (°): Ge1–Co1 2.303(1); P1–Co1 2.235(2); Co1–C32 1.974(6); N1–Ge1 1.860(6); C32–Co1–P1 167.6(2); N1–Ge1–Co1 109.3(2).

**Fig. 3:**
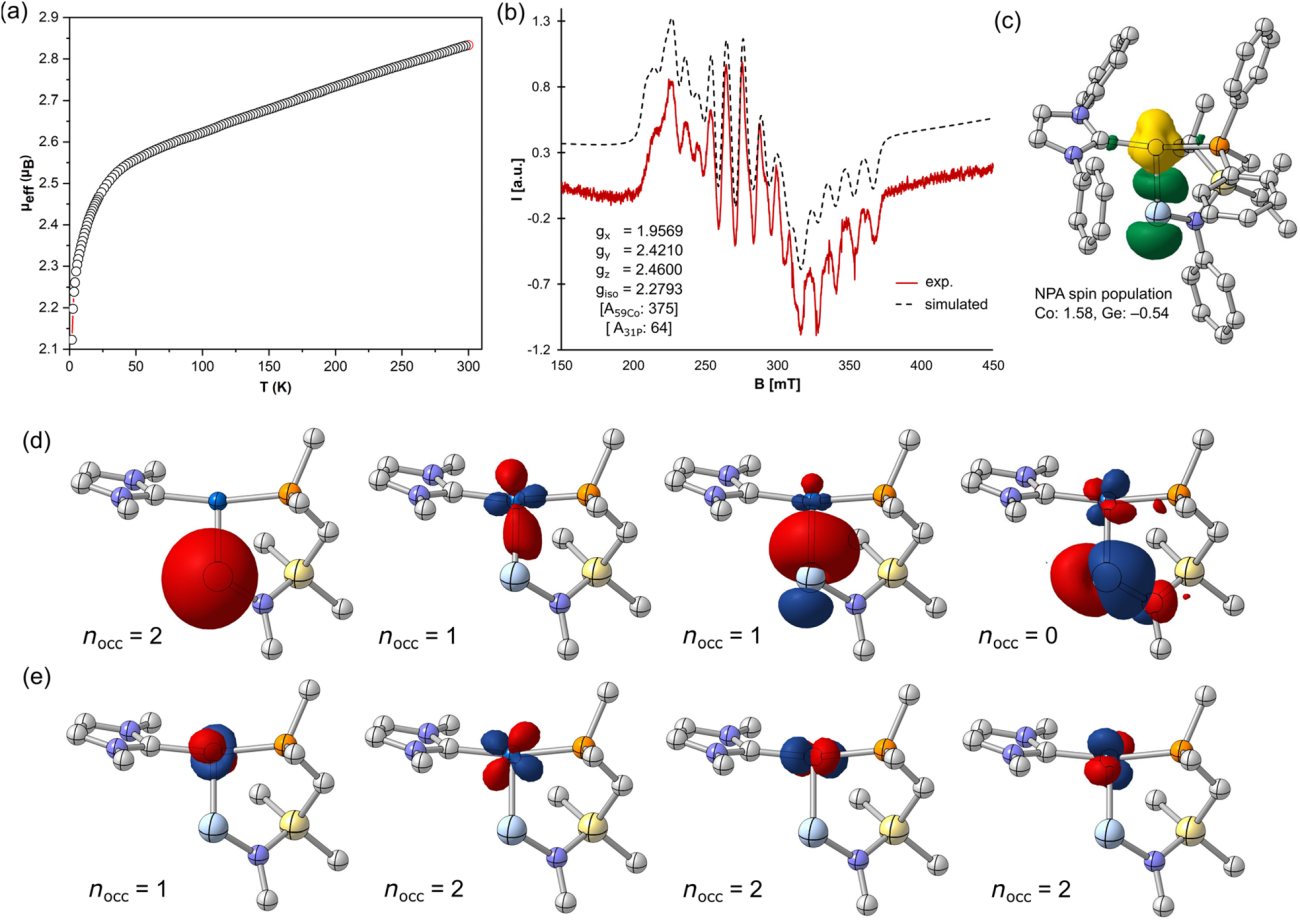
(a) Plot of the magnetic susceptibility of 2*vs.* temperature; (b) the experimental (red line) and simulated (dashed line) EPR spectrum for a toluene glass of 2 at 133 K; (c) spin-density plot of 2 with Co and Ge natural spin populations (hydrogen atoms are not shown, for clarity); (d) α/β averaged NLMOs representing an empty p-type orbital and a doubly occupied Ge-lone pair and two α- and β-NLMOs representing the Co–Ge bond; (e) NLMOs representing non-bonding electron density in d-orbitals; results for doubly occupied orbitals were obtained by averaging over the α and β spin orbitals.

We have recently demonstrated the utility of reported [IPr·M(η_2_-vtms)_2_] (IPr = [(H)CN(Dip)C:]; vtms = C_2_H_3_(SiMe_3_); M = Ni, Fe) complexes as efficient [IPr·M] transfer reagents.^37,43–45^ We therefore targeted related chemistry with Deng's [IPr·Co(η_2_-vtms)_2_].^46^ Addition of toluene to rapidly stirred and pre-cooled (−80 °C) solid mixtures of [^PhiP^DipGe][BAr^F^_4_] (1)^47^ and [IPr·Co(η_2_-vtms)_2_] (Scheme 1) led to an initial rapid colour change to dark green, becoming deep red upon warming to room temperature. After a further 12 h of stirring, the initial deep green colouration is restored. ^31^P{^1^H} NMR spectra for crude reaction mixtures are silent, indicative of the formation of a paramagnetic product. Removal of volatiles from these deep green mixtures and addition of pentane led to formation of large dichroic deep red–green crystals, X-ray structural analysis of which revealed the cationic Ge–Co complex 2 (Fig. 2), in which a distinct T-shaped geometry is observed at Co, isolated in up to 81% yield. This species represents a novel electronic situation for group 14 – cobalt complexes, given the low-coordinate nature of both Ge and Co, as well as the aforementioned T-shaped geometry. Generally, the dearth of base-free germylene–cobalt complexes, and indeed low-valent tetryl element–cobalt complexes in general, allows for little comparison with literature known systems. Complex 2 is perhaps best compared with Wesemann and co-worker's recently reported hydrido-germylene adduct of Co^0^, [Ar*Ge(μ-H)Co(PMe_3_)_3_] (B),^20^††We note that a related Co^I^ complex best described as a cationic cobaltogermylene was also reported in the same publication from Wesemann *et al.*, but metrical data was not obtained. It is thus not discussed here. whereby complex 2 differs in being geometrically constrained, more electron deficient, and indeed bearing a cationic charge. ‡‡We note that 2 crystallizes with two distinct molecules in the asymmetric unit. Only one is discussed here. Complex 2 contains a long Ge–Co bond distance of 2.303(1) Å, extended significantly from that in doubly-bonded B (*d* = 2.1918(4) Å), and closer to those seen in based-stabilised-germylene adducts of [Co_2_(CO)_*n*_] (*n* = 4, 5).^48^ A narrow N–Ge–Co angle of 109.3(2)° (*viz*. 145.2(1)° in B) would also imply a lone-pair of electrons at Ge. This is particularly apparent when comparing this angle to that in our T-shaped Ni^0^ complex A (109.7(1)°), in which the cationic germylene formally behaves as a Z-type ligand. This angle is significantly contracted relative to that in formally L-type germylene systems utilising the same ligand backbone (*e.g.*^PhiP^Dip(Ar)Ge·Ni·IPr; 116.26–118.16^°^).^49^ Finally, the CNHC–Co–P angle of 167.6(2)° aligns with that in the few known T-shaped Co^I^ complexes.^50–53^§§T-shaped species were identified *via* the CCDC, and are defined as those complexes with a 3-coordinate Co centre with an L-Co-L angle of >165°. One additional structural observation relates to the central 6-membered [GeCoPCSiN] ring in this complex, which forms a boat-conformation; this is apparently due to a strong agostic interaction between one ^i^Pr-CH moiety and the Co centre (*d*_Co–H16_ = 2.663 Å; Fig. S46 in SI), which lends additional stability to the low-valent Co centre. Key information pertaining to the electronic nature of 2 was acquired through SQUID magnetometry and EPR spectroscopy, in addition to computational analyses (Fig. 3). The magnetic moment ascertained by SQUID magnetometry (*μ*_eff_^298^ = 2.83 μ_B_, Fig. 3(a)) is somewhat higher than would be expected for the spin-only value of an *S* = ½ spin system (*i.e.* 1.73 μ_B_), likely due to spin–orbit coupling, a known effect for tetryl element complexes of the first-row TMs.^54,55^¶¶A similar effect was observed in our earlier reported open-shell iron complexes. See ref. 43. This effect is lessened in homogenous solutions of 2 as shown by the Evans method (*μ*_eff_^298^ = 2.1 μ_B_), yielding values which align with either a low-spin d^7^ (*i.e.* Co^II^) or a d^9^ (*i.e.* Co^0^) system. A linear increase in the inverse of the molar susceptibility *vs. T* yields a linear plot which intersects at 0 K (Fig. S3 in SI), indicative of typical Curie–Weiss paramagnetic behavior. The X-band EPR spectrum collected using a frozen toluene glass of 2 at 133 K yielded a somewhat broadened but resolved rhombic spectrum with clear hyperfine coupling to ^59^Co (Fig. 3(b)), and is similar to reported examples of germyl-cobalt(ii) systems.^56^ Given the complexity of this spectrum, *g*-values and hyperfine coupling constants were acquired from the fitted spectrum. Here, *g*-values of 1.9569, 2.4210, and 2.4600, giving a giso of 2.2793, agree with a cobalt centred electron. Significant hyperfine coupling to ^59^Co is observable, with a smaller degree of coupling to ^31^P (Table S1).

### In-depth computational analysis of 2

For further insights into the nature of the germanium cobalt interaction we performed quantum chemical calculations on the full molecular system of 2. Initial DFT calculations resulted in a UKS wave function with an increased 〈*S*^2^〉 value of 1.26 (0.75 is expected for a doublet), featuring strong spin polarization about the Co–Ge bond vector in addition to the expected spin density of the unpaired electron localized at the Co centre. In keeping with the EPR data reported above, the spin density plot in Fig. 3(c) shows significant spin density localized on Co, amounting to ∼75%, while ∼25% spin density resides on germanium. A natural bond orbital (NBO) analysis provides first implications on the nature of the germanium–cobalt interaction. The presence of an unoccupied p-type NLMO and an s-type lone pair NLMO both localised at germanium illustrates the germylene character of 2. Most notably, the presence of a single lone pair NLMO at germanium (Fig. 3(d) and S25) rules out its partaking in a dative Ge → Co interaction. Further, four non-bonding NLMOs representing the Co 3d orbitals are found, three doubly occupied, and the singly-occupied d_*z*_^2^ orbital, *i.e.* the spin carrying NLMO (Fig. 3(e)). This situation indicates a formal Co^II^(d^7^) species. Two NLMOs represent the spin-polarized germanium–cobalt interaction, an α NLMO polarized towards cobalt and a β NLMO polarized towards germanium (α and β NLMO shown in Fig. 3(d)).

We attribute the occurrence of this broken symmetry solution to the so-called primogenic repulsion:^57–59^||||We note the absence of any spin-polarization in the corresponding (experimentally unknown) rhodium and iridium complexes, *cf.* SI. Due to the compact nature of the 3d orbitals in first-row TM complexes, Pauli repulsion between the metal sub-valence shell and ligand electrons leads to stretched bonds with poor orbital overlap, generally increasing the importance of non-dynamic electron correlation effects. In our case, this is further aggravated by size mismatches of the interacting orbitals of cobalt and germanium. The observed spin-polarisation in the Co–Ge bonding region arises as a consequence of the pertinent strong non-dynamic correlation effects, which are qualitatively captured within approximate DFT by means of a broken-symmetry (BS) character in unrestricted Kohn–Sham (UKS) wave function representations. While such wave functions relate to clearly unphysical spin densities, the corresponding electron densities as such are qualitatively correct also for multireference (MR) cases.^60,61^

For further scrutiny we performed explicitly correlated multi-reference configuration interaction (MRCI-F12) calculations based on Complete Active Space Self Consistent Field (CASSCF) wave functions on a small molecular model as a benchmark (*cf.* SI). An active space comprising five electrons in five orbitals was found to capture all major non-dynamic correlation effects and a computationally much less demanding perturbative treatment of dynamic correlation by means of NEVPT2 calculations reproduce the benchmark results well. The following bonding analyses on 2 were thus performed at this level of theory (*cf.* SI). These results revealed considerable multi-reference character, with configuration mixing predominantly involving the Ge–Co bonding and antibonding orbitals. This aligns well with the aforementioned broken-symmetry DFT results. Based on the population of these two correlating natural orbitals in the NEVPT2 wave function, Truhlar's M diagnostic of 0.223 substantiates this notion, indicating a pronounced multi-reference character similar to that in the prototypical ozone case.^62^ Equivalent results were obtained for the *n*_rad_ index^63^ computed either based on the 〈*S*^2^〉 expectation value of the UKS wave function or based on the double-excitation CI coefficient from CASSCF/NEVPT2 calculations. All in all, we attribute the spin polarisation along the Ge–Co bond observed in UKS calculations to the recovery of strong non-dynamic electron correlation effects in 2 – the excess spin-density along this bond is merely a non-physical, technical artefact (*cf.* SI for a detailed presentation of results).

**Fig. 4:**
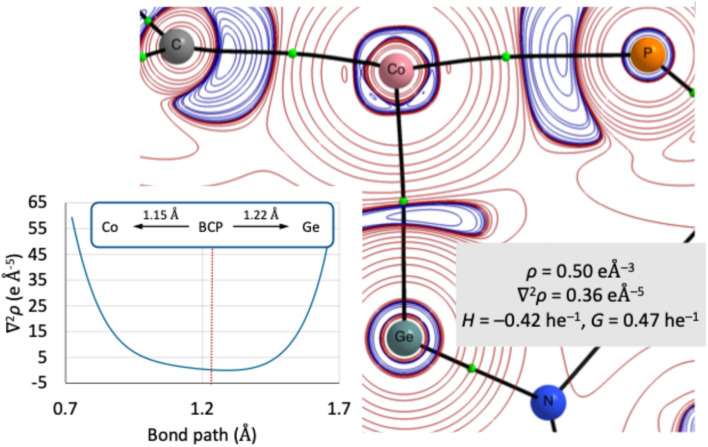
2D plot of ∇^2^*ρ*(r) in the P–Co–Ge plane of 2 with characteristics at the Ge–Co bond critical point, charge accumulation (blue), depletion (red), bond paths (black lines), bcps (green dots). Inset: 1D bond path graphical plot.

With the above results in hand, we performed bonding analyses of the electron density in 2 by means of the quantum theory of atoms in molecules (QTAIM). The analysis obtained from CASSCF(5,5)/NEVPT2 calculations gives a Ge–Co bond path with a bond critical point (bcp, Fig. 4); the corresponding 1D Laplacian profile along the bond path is rather symmetrical with the bcp shifted slightly towards the cobalt atom (Fig. 4, inset). The distinct nature of the Ge–Co bond compared to the other cobalt-ligand bonds is highlighted by comparison of the respective bcp characteristics. The latter bonding interactions are characterized by a low value of *ρ*(r_bcp_), a positive Laplacian ∇^2^*ρ*(r_bcp_), a negative relative total energy density *H*(r_bcp_), and a relative kinetic energy density *G*(r_bcp_) of approximately 1; this set of criteria is typical for donor–acceptor interactions.^64^ At the Ge–Co bcp, however, we also find a low density *ρ*(r_bcp_) and a negative *H*(r_bcp_), whilst the Laplacian is close to 0 and *G*(r_bcp_) is smaller than 1. These characteristics are consistent with a covalent, electron-sharing metal–metal interaction between Ge and Co, supporting the notion of 2 as a cobalto-germylene. As bcps are generally shifted along their associated bond path towards the more electropositive element,^65^*i.e.* Co, we assign a formal +2 oxidation state to cobalt in line with described NBO results.

Further analysis of the electron density employing the electron localization function (ELF) reveals a disynaptic basin between germanium and cobalt with a population of 1.67 and a variance of 1.20. Superposition of ELF and QTAIM basins allows for an evaluation of atomic contributions to the ELF basin:^66^ here, germanium contributes 1.03 electrons and cobalt 0.62 electrons to the shared basin. Comparison with the Co–P/C_NHC_ basins illustrates the distinct nature of the Ge–Co bond. The overall population of the corresponding disynaptic basins is higher for the former bonds and, most notably, cobalt contributions to the basins are significantly lower than those of the P/C_NHC_ atoms, whereas the Ge–Co basin shows more evenly distributed atomic contributions by comparison (see Table S7–S9 in SI).

Considering this collection of experimental and computational results, complex 2 is best described as a cationic, open-shell cobalto-germylene, whereby the germanium centre bears an empty p-type orbital and an s-type lone pair. Unpaired electron density is largely localised at Co, with a low-spin d^7^ (*i.e.* Co^II^) electronic configuration. As such, oxidative addition processes occur at Co in the course of the formation the unique cobalto-germylene 2 – the mechanism for such processes warrants further exploration.

### Mechanistic studies for the formation of 2

**Scheme 2:**
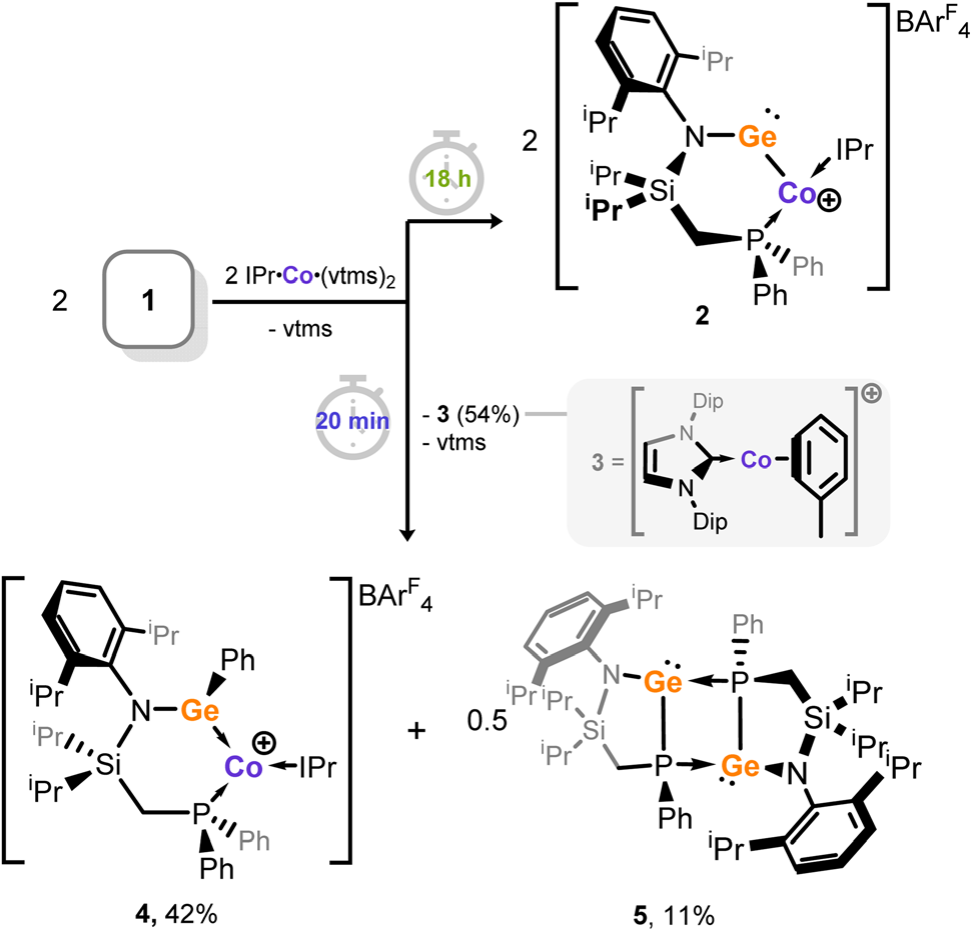
The formation of species 3, 4, and 5 on shortening the reaction time between 1 and [IPr·Co(η_2_-vtms)_2_], leading to complex fragmentation. Presented yields refer to isolated crystalline solids.

**Fig. 5:**
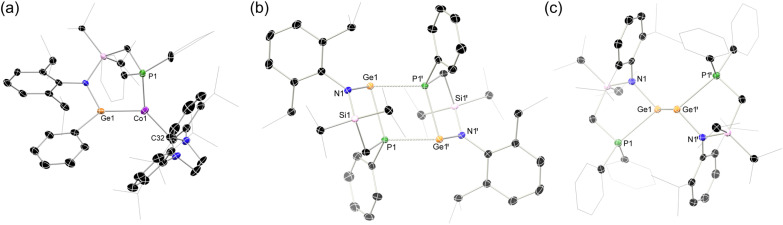
The molecular structure of (a) the cationic part in 4, and the full molecular structures of (b) 5, and (c) 7, with ellipsoids at 30% probability, and hydrogen atoms omitted for clarity. Selected bond lengths (Å) and angles (°) for 4: Co1–Ge1 2.334(2); Co1–C32 2.011(8); Co1–P1 2.727(2); Ge1–Co1–C32 131.3(2); C32–Co1–P1 139.8(2); Ge1–Co1–P1 87.82(7); N1–Ge1–C59 108.3(3). For 5: Ge1–P1 2.4759(7); Ge1–P1′ 2.532(1); N1–Ge1 1.918(2); P1–Ge1–P1′ 74.83(2); Ge1–P1–Ge1′ 105.17(2). For 7: Ge1–Ge1′ 2.6402(9); Ge1–N1 1.933(4); Ge1–P1 2.647(1); N1–Ge1–Ge1′ 100.8(1); P1–Ge1–Ge1′ 109.89(3); N1–Ge1–P1 88.0(1).

As described, the reaction of cationic germylene 1 with [IPr·Co(η_2_-vtms)_2_] proceeds through several colour changes leading to the final product, 2, after 18 h stirring. Upon closer inspection of these reaction mixtures, a pale green precipitate is observed soon after the reaction becomes deep red, *i.e.* within the first 20 min of the reaction. Isolation of this solid by filtration and recrystallisation allowed for the structural elucidation of this species, found to be the Co^I^ cation [IPr·Co(η_6_-tol)][BAr^F^_4_] (3, Scheme 2), which was recently reported by us.^67^ Storage of the remaining reaction solution allowed for the crystallization of two further species: first, an additional cationic Co^I^ complex is found (4; Scheme 2 and Fig. 5(a)), bound by our previously reported (amido)(aryl)-germylene ^PhiP^DipGePh (6).^49^ We presume this germylene arises through formal intermolecular activation of one P–Ph unit of the ^PhiP^Dip ligand. With this point in mind, and balancing the overall reaction equation, we should also observe the neutral phosphido-germylene 5 (Scheme 2); this is presumed to arise through reductive P–Ge bond formation and Ph-transfer (*i.e.* in the concomitant formation of 6). Remarkably, compound 5 can also be crystallised from these reaction mixtures, isolated as its dimer in the solid state (Fig. 5(b)). Notably, these fragmentation products are only isolated when precipitated 3 is removed from reaction mixtures by filtration, indicating that this fragmentation process is feasible only with sub-stoichiometric quantities of 3.

Both complexes 3 and 4 are paramagnetic, open-shell d^8^ Co^I^ complexes. As mentioned, the former arene-coordinated system was recently reported by us, synthesised *via* oxidation of [IPr·Co(η_2_-vtms)_2_],^67^ and bears resemblance to a small number of cationic Co^I^-arene systems in the literature (*e.g.* chelating diphosphine species).^68^ As such, we turn our attention to Ge^II^–Co^I^ complex 4, which is somewhat more interesting in the context of this study. This species bears a neutral germylene ligand bound to a high-spin open-shell Co^I^ centre (*i.e. S* = 1), borne out by the SQUID-derived *μ*_eff_^298^ of 3.54 μ_B_ (Fig. S13 and S14; Evans method: 3.12 μ_B_). The Ge–Co bond in 4 is longer even than that in 2 (*d*_CoGe_: in 2 = 2.292(2) Å; in 4 = 2.334(2) Å), despite the now formal L-type germylene ligand and cationic cobalt centre. This is most likely due to both the dative Ge–Co bond and the high-spin nature of the cobalt centre. The electron deficient, *i.e.* 14-electron Co^I^ centre in 4 leads to a strong puckering of the central 6-membered ring in this complex, on forming two close agostic interactions with one Si–^i^Pr fragment of the ligand backbone (*e.g. d*_Co1H14c_ = 2.473 Å).

On the mechanism of the above described fragmentation process, one can simplify the products formed to two equiv. of an [NHC·Co^I^]^+^ species (*e.g.*3), the dimeric (amido)(phosphido)germylene 5, featuring a newly formed P–Ge single bond, and the (amido)(phenyl)germylene ligand 6. Under the reaction conditions, the cobalt(i) species 3 combines with germylene 6 in the formation of complex 4; this is confirmed using independently synthesised samples of 3 and 6.^49,67^ Overall, then, Co^0^ performs a one-electron reduction of cationic germylene ligand 1. This ultimately leads to the formation of 5 and 6 – though both species contain Ge^II^, the former bears a phosphide ligand, which has thus undergone a 2-electron reduction from P^III^ to P^I^. This species was independently synthesized to unequivocally confirm its connectivity (see SI for details).

**Fig. 6:**
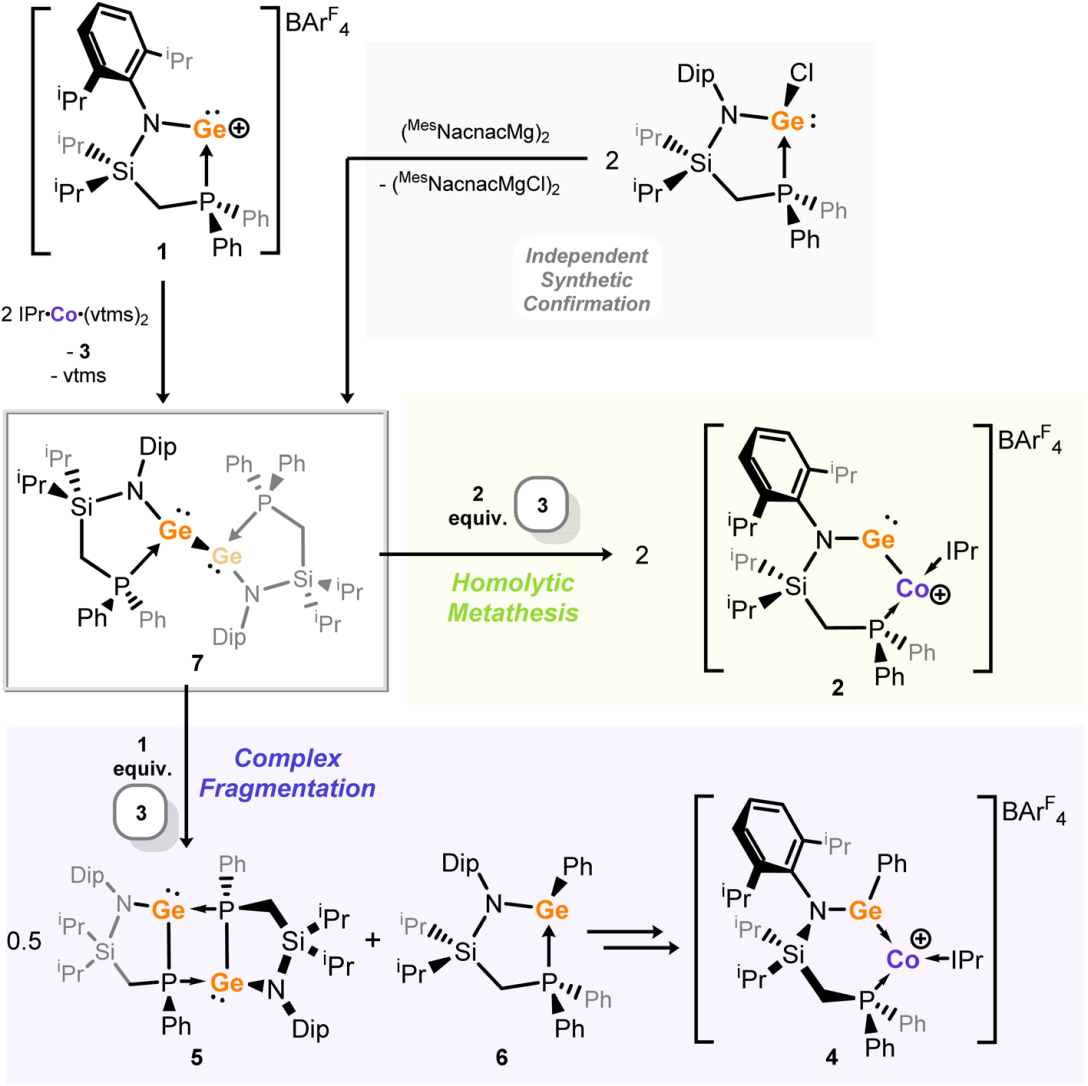
The reaction map for the initial formation of bis-germylene 7 through reduction of 1 by Co^0^, (confirmed by its independent synthesis using a dimeric Mg^I^ compound), followed by the stoichiometry-dependent reactivity of 7 towards cationic cobalt species 3, leading to either cobalto-germylene 2, or species 4–6.

We then looked towards the root of this fragmentation reaction, aiming to gain insights into the overall mechanism for the formation of cobalto-germylene 2. As described, the formation of a Co^I^ species in the initial stage of this reaction (*viz*. 3) suggests that a Ge^I^ species is formed, *i.e.* through single-electron reduction of Ge^II^ species 1. Therein, the reaction for the formation of 2 was conducted, and the solution filtered following precipitation of cobalt(i) cation 3. By maintaining low temperatures during work-up, we were fortunate to obtain a small crop of orange-green dichroic crystals found to be the digermyne 7, formally a dimer of two [^PhiP^DipGe^I^] fragments (Fig. 6 and 5(c)). This species is structurally similar to previously reported base-stabilised dimeric germanium(i) compounds,^69–71^ and will not be discussed in depth here. Importantly, this species can be directly formed by the reduction of the chloro-germylene ^PhiP^DipGeCl by the Jones Mg^I^ dimer,^72^ and isolated in good crystalline yield (see SI for details). The steric encumbrance around the central [Ge–Ge] bond is borne out by the significant broadening of peaks in the ^1^H NMR spectrum of this compound. The single resonance in the ^31^P{^1^H} NMR spectrum is similarly broadened (*δ* = 0.2 ppm; FWHM = 118 Hz). In addition, a LIFDI mass spectrum of this species clearly demonstrates the presence of both dimeric 7 and its monomeric ‘half-peak’ (Fig. S29 and S30 in ESI). These points suggest that the Ge–Ge bond may be readily cleaved.

Reaction of this low oxidation-state germanium species with cationic Co^I^ species 3 in a 1 : 1 stoichiometry (Fig. 6) does indeed lead to the fragments depicted in Scheme 2: (amido)(phosphido)-germylene 5 and (amido)(phenyl)-germylene 6 are clearly observed in both the ^1^H and ^31^P{^1^H} NMR spectra for this reaction mixture (Fig. S37–S39 in SI), whilst the broad paramagnetic signals for cobalto-germylene 2 are observed in the ^1^H NMR spectrum (Fig. S40 in SI). Thus, the effective mechanism in formation of 2 from [IPr·Co(η_2_-vtms)_2_] and cationic germylene 1 most likely proceeds first in reduction of Ge^II^ by Co^0^, forming 3 and 7. This is then followed by a formal homolytic cleavage of the Ge–Ge bond in 7 by Co^I^, leading to 2 (Fig. 6). This is somewhat related to the metathesis of group 14 element(I) dimers by dimeric Mo^I^ species, reported by Power and co-workers,^73^ which similarly led to E–E bond scission (E = Ge, Sn, Pb) and E–Mo bond formation. This thus opens an exciting new strategy for the formation of heteroatomic main group-transition metal complexes using the vast number of established monomeric low-valent transition metal synthons, which we now look towards exploring more broadly in our laboratories.

### Lewis base coordination in 2

**Scheme 3:**
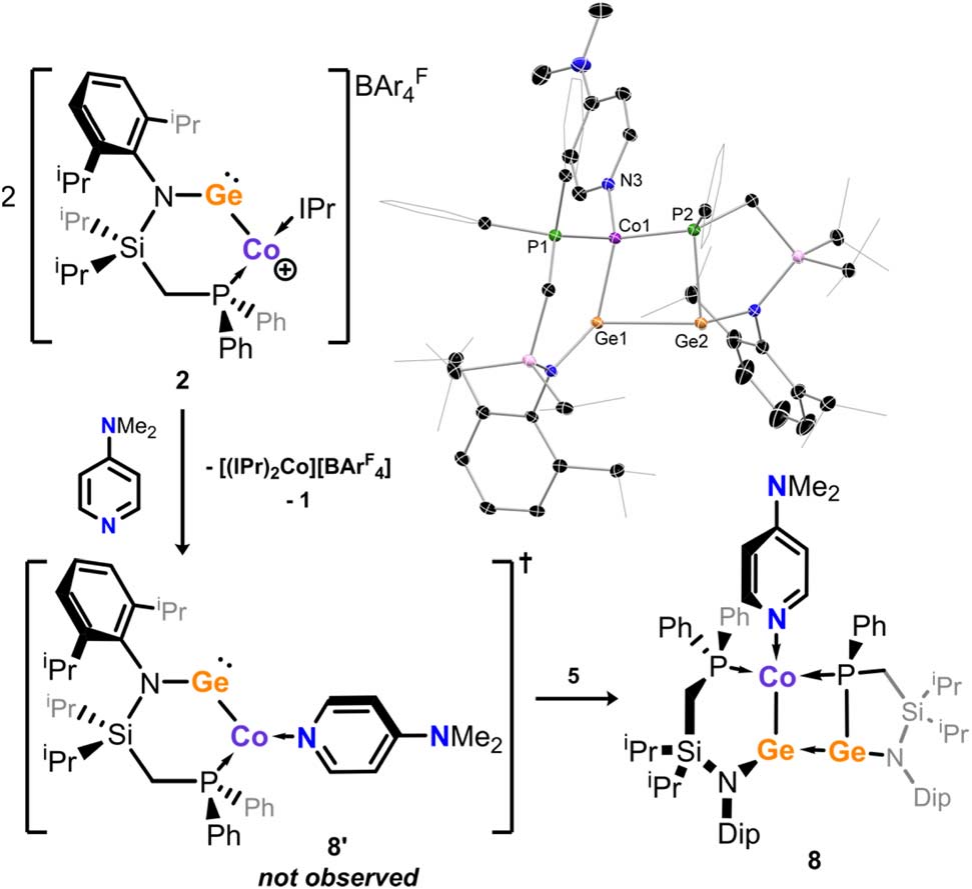
Hypothesised pathway for the formation of 8, upon addition of *N*,*N*-dimethylaminopyridine to 2. Inset: the molecular structure for compound 8.

Given the fragmentation products isolated on the synthetic pathway to 2, we aimed to further define the apparent dynamic behaviour of this species in solution. Cyclic voltammetry experiments using THF solutions of 2 with the ferrocene reference electrode are further indicative of a complex solution behaviour: a quasi-reversible reduction event is observed at *E*_1/2_ = −1.49 V, whilst numerous irreversible oxidation events are found, with *E*_ox_ values between 0.24 and 1.03 V (Fig. X–X in SI). We thus focused on direct coordination chemistry, whereby dissolved 2 was reacted with Lewis basic *N*,*N*-dimethylaminopyridine (DMAP), hoping to stabilise cationic 2 through coordination at Ge. From these solutions red powders could be isolated in low yield, which were found to be highly soluble in pentane, precluding cationic character. Recrystallisation revealed this product to be a unique [Ge_2_Co] complex, 8 (Scheme 3). Analysis of structural parameters in this species would suggest two dative P–Co bonds (*d*_Co1P1_ = 2.124(1) Å; *d*_Co1P2_ = 2.154(1) Å), a formal Ge2–P2 bond (*d*_Ge2P2_ = 2.373(1) Å), a formal Ge1–Co1 bond (*d*_Ge1Co1_ = 2.1825(8) Å), and a long Ge–Ge bond (*d*_Ge1Ge2_ = 2.723(1) Å). Thus, the best description of 8 is a DMAP-coordinated cobalto-germylene (*vis*. 8′, Scheme 3), side-on coordinated by the phosphido-germylene 5 which may be generated upon dissolution of 2. Detailed electronic structure analysis reveals four non-bonding NLMOs representing the Co 3d orbitals, all doubly occupied (*cf.* SI for details); this situation is indicative of a formal Co^I^(d^8^) species. This is consistent with its diamagnetic nature: a complex but well resolved ^1^H NMR spectrum is observed for 8 in solution. The corresponding ^31^P NMR spectrum displays two slightly broadened doublets, with a clear 2*J* coupling for these signals (*δ* = 33.8 and 53.7 ppm, ^2^*J*_PP_ = 103.7 Hz), as expected based on the unsymmetrical molecular structure of 8, with one phosphine and one phosphide moiety.**Though small quantities of 8 can be isolated, it does decompose over time in solution, and as such a well resolved ^13^C NMR spectrum for this species could not be successfully acquired. The formation of this complex further demonstrates the dynamic bond-activation processes at play in solution involving the described low-valent Ge–Co systems. Whilst this has prevented well-defined reactivity studies concerning electronically unique T-shaped complex 2, this does highlight potential reactive pathways for this new class of complex.

## Conclusions

Herein we have described the synthesis and electronic characterization of the first example of an open-shell cobalto-germylene complex, featuring a unique T-shaped, low-spin Co^II^ centre. In conjunction with EPR spectroscopy and SQUID magnetometry, multi-reference computational methods indicate a *S* = ½ d^7^ Co complex, with a high spin-density at this metal centre. The Co–Ge bond is characterized as an electron-sharing covalent bond that features strong non-dynamical correlation effects. Though this species can be accessed in high yield, its formation is not trivial. It initially appears as through direct addition of the cationic germylene to Co^0^ is the formal pathway, but deeper mechanistic studies suggest the initial reductive formation of a germanium(i) dimer, which is ultimately homolytically cleaved by Co^I^ in formation of the cobalto-germylene. The isolation of several species which arise from fragmentation of these intermediates shed light on the dynamic behavior of the covalent interactions in this remarkable complex class. We are presently developing more robust ligand systems as to allow for further investigations which direct this dynamic reactivity towards well-defined catalytic coupling processes, as well as cooperative bond activations at the Ge–Co interface.

## Author contributions

AS carried out all experimental and analytical work. MH carried out all computational work. MCH supervised and devised the computational work. TJH supervised the experimental aspects of this work, and devised the study.

## Conflicts of interest

There are no conflicts to declare.

## Supplementary Material

SC-016-D5SC04265H-s001

SC-016-D5SC04265H-s002

SC-016-D5SC04265H-s003

## Data Availability

CCDC 2382130 (2), 2382132 (4), 2382129 (5), 2434139 (7), and 2382131 (8) contain the supplementary crystallographic data for this paper.^74*a*–*e*^ The data supporting this article have been included as part of the SI. Supplementary information: synthetic protocols for all new compounds, a summary of their analytical data, experimental spectra, a summary of X-ray crystallographic methods and data, and an in-depth description of computational methodology, including additional notes to the support the main text. See DOI: https://doi.org/10.1039/d5sc04265h.
