# Supplementary material for: The path to an open-shell metallo-germylene: direct ligation, or reduction and metathesis?
Source: Chem Sci. 2025 Sep 16;16(43):20265–74. doi: 10.1039/d5sc04265h (PMC12505228; doi:10.1039/d5sc04265h)
Supplement: SC-016-D5SC04265H-s001 [file SC-016-D5SC04265H-s001.pdf]

Supplementary Information (SI) for Chemical Science.
This journal is © The Royal Society of Chemistry 2025

The Path to an Open-Shell Metallo-Germylene: Direct Ligation, or Reduction and Metathesis?

Annika Schulz,[a] Myron Heinz,[b] Max. C. Holthausen,*[,b] and Terrance J. Hadlington*[,a]

[a] Lehrstuhl für anorganische Chemie mit Schwerpunkt neue Materialien, School of Natural Sciences, Technische Universität München, Lichtenberg Strasse 4, 85747 Garching

[b] Institut für Anorganische und Analytische Chemie, Goethe-Universität Frankfurt, Max-von-Laue-Strass 7, 60438, Frankfurt

1. Experimental methods and data……………………..S2

General Considerations…………..………………………….S2

Synthetic details and data………………………………..…S4

NMR, MS, UV/vis, and IR spectra…………………………....S9

2. X-ray crystallographic details…………………….….S30

3. Computational methods and details………………..…S33

4. References…………………………………………....S52

General considerations. All experiments and manipulations were carried out under dry oxygen free argon atmosphere using standard Schlenk techniques or in an MBraun inert atmosphere glovebox containing an atmosphere of high purity argon. THF and diethyl ether were dried by distillation over a sodium/benzophenone mixture and stored over activated 4Å mol sieves. C_6D_6 was dried, degassed and stored over a potassium mirror. All other solvents were dried over activated 4Å mol sieves and degassed prior to use. ^{PhiP}DippGeCl (^{PhiP}Dipp = {[$Ph_2PCH_2Si(^iPr)_2$](Dipp)N}; Dipp = 2,6-$^iPr_2C_6H_3$),[1] ^{PhiP}DippGePh (**6**),[2] [^{PhiP}DippGe][BAr^F_4],[3] IPr·Co·[η^2-(tmvs)$_2$],[4] and [(^{Mes}nacnac)Mg]$_2$ (^{Mes}nacnac = [HC{MeCNMes}$_2$]$^-$; Mes = 2,4,6-$Me_3C_6H_2$) [5] were synthesized according to known literature procedures. All other reagents were used as received.

NMR

NMR spectra were recorded on a Bruker AV 400 Spectrometer. The ^{1}H and ^{13}C{^{1}H} NMR spectra were referenced to the residual solvent signals as internal standards. ^{29}Si{^{1}H} NMR spectra were externally calibrated with $SiMe_4$; for ^{29}Si NMR spectra, Insensitive nuclei enhanced by polarization transfer (INEPT) methods were used for signal enhancement. ^{31}P{^{1}H} NMR spectra were externally calibrated with H_3PO_4.

MS

Liquid Injection Field Desorption Ionization Mass Spectrometry (LIFDI-MS) was measured directly from an inert atmosphere glovebox with a Thermo Fisher Scientific Exactive Plus Orbitrap equipped with an ion source from Linden CMS.[6]

UV/vis

Absorption spectra (UV/vis) were recorded on an Agilent Cary 60 UV/vis spectrophotometer fitted with a fiberoptic coupler feeding into an inert atmosphere glovebox under Ar. Sample preparation was conducted in an argon-filled glovebox by dissolution of each compound in the respective solvent, and the samples collected using a fibreoptic absorption dip-probe, which is directly lowered into each sample. Dilution was conducted in the glovebox as required.

EA

Elemental analyses (C, H, N) were performed with a combustion analyzer (elementar vario EL, Bruker).

SQUID/Magnetic susceptibility measurements

Magnetic susceptibility data collected on solid samples were recorded using a MPMS XL 5 (Quantum Design) superconducting quantum interference device (SQUID) magnetometer with

liquid Helium cooling in a temperature range of 1.8–300 K and a magnetic field of 1.0 T. The samples were placed in a calibrated gelatin capsule and fixed in the center of a plastic straw. Data were corrected for underlying diamagnetism using tabulated Pascal's constant and corrected for the magnetism of the gelatin capsule by comparison to a measurement of the capsule without compound.

EPR

EPR measurements were carried out using a JEOL JES-FA 200 spectrometer at X-band frequency (approximately 9.05 GHz, sweep width 325 mT, modulation frequency 100 kHz, modulation amplitude 0.4 mT, microwave power 5.0 mW). The g values were determined using Mn_{2+} (nuclear spin I = 5/2) embedded in MgO as a standard (fourth line g = 1.981). The temperature was monitored with a JEOL DVT4 temperature controller. Sample preparation was conducted in an argon-filled glovebox by dissolution of ground single crystals of **2** in toluene. The samples were frozen in liquid nitrogen prior to the measurements. The spectra were plotted using Origin Pro2016G (Origin Lab). The EPR spectrum of complex **2** was simulated using the EasySpin software program (Development version 6.0.0-dev.39).[7] The hyperfine couplings to the P and Co atoms were considered in order to simulate the spectra; a line width of 2 mT, a modulation amplitude of 0.4 mT, and microwave frequency of 9.05 GHz were assumed.

Cyclic Voltammetry

Cyclic Voltammetry was carried out with a PalmSens PalmSens4 potentiostat, using 3 mm diameter glassy carbon disk electrodes (ALS Japan) as working electrode and a platinum wire as counter electrode. Prior to use, electrodes were polished with 0.05 µm alumina suspensions (CH Instruments Inc., USA). Ag/Ag^+ (10 mM $AgBF_4$ and 0.1 M $[N(n\text{-}Bu)_4]PF_6$ in THF) was used as the reference electrode, separated via frit. CV measurements were performed in a five-necked glass cell under an Ar atmosphere. Potentials are reported with reference to an internal standard of ferrocenium/ferrocene.

Syntheses details and data

[^{PhiP}DippGe·Co·IPr][BAr$^F_4$], 2. A solid mixture of [^{PhiP}DippGe][BAr$^F_4$] (700 mg, 0.52 mmol) and IPr·Co·[η^2-(vtms)$_2$] (334 mg, 0.52 mmol) was cooled to -80 °C, and toluene (30 mL) was added, with rapid stirring. The mixture immediately became dark green, and was subsequently allowed to warm to ambient temperature. The solution became dark red upon warming, and eventually became dark brown after 5min, with concomitant formation of a yellow-green precipitate. After stirring for a further 20min, this solid redissolved. After stirring overnight, the reaction mixture had become dark green. At this stage, all volatiles were removed *in vacuo*, and pentane added to the residue leading to the formation of deep green-purple crystals, which were isolated by filtration and washing with small amounts of cold toluene, and dried under vacuum, yielding **2** as a deep green powder (750 mg, 0.42 mmol, 81%). Crystals grown from these solutions were found to be significantly twinned, leading to unsuitable data for publication. However, recrystallisation from C_6H_5F/toluene (4:1) mixtures lead to the formation of a large green-purple dichroic crystals suitable for X-ray diffraction analysis.

^{1}H NMR (THF-*d*$_8$, 400 MHz, 298 K): 2.31, 2.76, 4.24, 5.54, 6.56, 7.56, 7.78, 8.26, 9.75, 11.34.

Magnetic moment (Evans' method; THF-*d*$_8$, 400 MHz, 298 K): 2.10 μ_B; (SQUID; crystalline solid, 298 K): 2.83 μ_B.

MS/LIFDI-HRMS found (calcd.) m/z: 1009.4334 (1009.4325) for [M-BAr$^F_4$]$^+$

Anal. calcd. for $C_{90}H_{91}BCoF_{24}GeN_3PSi$: C, 57.74%; H, 4.90%; N, 2.24%; found C, 57.24%; H, 4.88%; N, 2.26%.

λ_{max}, nm (ε, Lcm^{-1}mol^{-1}): 597 (560), 423 (1480), 340 (4080).

[IPr·Co·(η^6-C_7H_8)][BAr$^F_4$], 3. The reaction was conducted as per that for **2**: [^{PhiP}DippGe][BAr$^F_4$] (700 mg, 0.52 mmol) and IPr·Co·[η^2-(tmvs)$_2$] (334 mg, 0.52 mmol) were reacted at -80°C in toluene (30 mL), and brought to room temperature over the course of ~5min, leading to a red-brown solution over a light yellow-green precipitate. At this stage, the solution was separated from the solid by filtration, and washed with pentane (10 mL). Drying of the powder under vacuum allowed for the isolation of an analytically pure light green powder (369 mg, 0.28 mmol, 54%), identified as **3** by comparison with literature data for that species.[8] This was additionally confirmed by recrystallisation of this powder, and collection of single-crystal XRD data for this species.

^{1}H NMR (THF-*d*$_8$, 400 MHz, 298 K): -4.38, -0.93, 1.92, 7.56, 7.79, 8.11, 10.22 11.69.

Magnetic moment (Evans' method; THF-*d*$_8$, 400 MHz, 298 K): 2.85 μ_B.

MS/LIFDI-HRMS found (calcd.) m/z: 539.2811 (539.2836) for [M-BAr$^F_4$]$^+$

Anal. calcd. for $C_{66}H_{56}BCoF_{24}N_2$: C, 56.51%; H, 4.02%; N, 2.00%; found C, 56.39%; H, 4.04%; N, 1.99%.

[^{PhiP}DippGePh·Co·IPr][BAr$^F_4$], 4.

Method A. The reaction was conducted as per that for **2**: [^{PhiP}DippGe][BAr$^F_4$] (700 mg, 0.52 mmol) and IPr·Co·[η^2-(tmvs)$_2$] (334 mg, 0.52 mmol) were reacted at -80°C in toluene (30 mL), and brought to room temperature over the course of ~5min, leading to a red-brown solution over a light yellow-green precipitate. At this stage, the solution was separated from the solid by filtration. All volatiles were removed from the supernatant solution, resulting in a brown oil. Addition of pentane (20 mL) to this oil led to the formation of deep red crystals suitable for X-ray diffraction analysis, confirming the structure of **4**. The remaining crystals were separated from the solvent by filtration, and dried *in vacuo*, yielding **4** as a dark red powder (200 mg, 0.11 mmol, 42%).

Method B. To a solution of ^{PhiP}DippGePh (300 mg, 0.47 mmol) in PhF (5 mL) at -80 °C, [IPr·Co·(η^6-C_7H_8)][BAr$^F_4$] (627 mg, 0.47 mmol) in PhF was slowly added. The reaction mixture immediately turned dark brown, changing to pink after warming to room temperature. The reaction was stirred for further 30min and subsequently filtered. The solution was concentrated to ~5 mL, and pentane (~20 mL) added, leading to the formation of a pink-red solid, which was separated from the supernatant solution by filtration. After washing with pentane (5 mL), **4** could be obtained as a red-pink powder (698 mg, 0.37 mmol, 79%).

^{1}H NMR (THF-*d*$_8$, 400 MHz, 298 K): -24.52, -15.80, -9.91, -8.36, -5.91, -3.00, -2.99, -1.87, 1.64, -1.40, -0.70, 3.12, 3.35, 4.06, 4.88, 5.08, 6.27, 6.28, 6.72, 7.55, 7.77, 8.23, 8.34, 9.62, 12.72, 13.07, 16.17, 17.42, 20.29, 20.31, 24.37, 38.27, 40.78, 41.67.

Magnetic moment (Evans' method; THF-*d*$_8$, 400 MHz, 298 K): 3.50 μ_B; (SQUID; crystalline solid, 298 K): 3.55 μ_B.

Anal. calcd. for $C_{96}H_{96}BCoF_{24}GeN_3PSi$: C, 59.15%; H, 4.96%; N, 2.16%; found C, 57.17%; H, 4.82%; N, 2.10%.

MS/LIFDI-HRMS found (calcd.) m/z: 1086.4682 (1086.4716) for [M-BAr$^F_4$]$^+$

λ_{max}, nm (ε, Lcm^{-1}mol^{-1}): 594 (396), 425 (1080), 344 (3000).

(^{PhiP}DippGePPh)$_2$, 5.

Method A. The reaction was conducted as per that for **2**: [^{PhiP}DippGe][BAr$^F_4$] (700 mg, 0.52 mmol) and IPr·Co·[η^2-(tmvs)$_2$] (334 mg, 0.52 mmol) were reacted at -80°C in toluene (30 mL), and brought to room temperature over the course of ~5min, leading to a red-brown solution

over a light yellow-green precipitate. At this stage, the solution was separated from the solid by filtration. All volatile were removed from the supernatant solution, resulting in a brown oil. To this, pentane (20 mL) was added leading to the formation of dark red crystals of **4**. The supernatant solution was then separated and concentrated to ~3 mL. After storing the solution for a few days at -35 °C, colourless crystals of **5** formed, which were suitable for X-ray diffraction analysis. The supernatant solution was decanted and the crystals dried *in vacuo*, yielding dimeric **5** as a white solid (28 mg, 0.012 mmol of dimer, 0.024 mmol of monomer, 11%).

Method B.

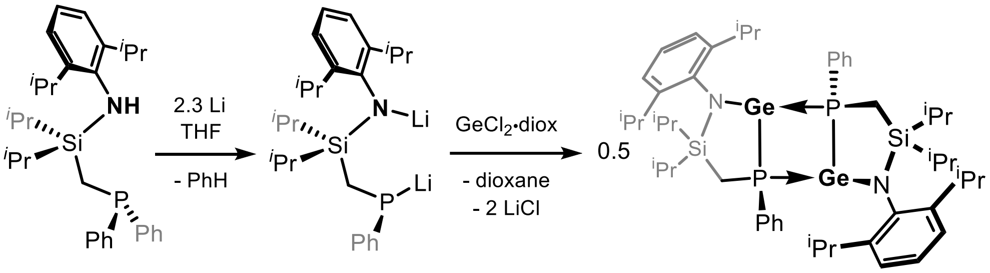

To a solution of ^{PhiP}DippNH (1.00 g, 2.04 mmol) in THF (50 mL) was added lithium granules (32.6 mg, 4.70 mmol). Stirring overnight led to a yellow solution, which was added dropwise *via* a filter canula to $GeCl_2$·1,4-dioxane (473 mg, 2.04 mmol) as a solution in 20 mL THF cooled to -80°C. The reaction mixture was kept at this temperature for three hours, before allowing it to warm up to ambient temperature, over which time the colour of the reaction mixture gradually changed from yellow to dark orange. After stirring for 30 minutes at ambient temperature, all volatiles were removed *in vacuo*. The remaining red solid was washed with cold pentane (15 mL) before the remaining residue was extracted with toluene (30 mL). All volatiles were removed *in vacuo* and **5** was obtained as an off-white solid (280 mg, 0.58 mmol, 28%).

^{1}H NMR (C_6D_6, 400 MHz, 298 K): 0.72 (d, 6H, $^3J_{HH}$ = 7.2 Hz, Si-Pr^i-C*H$_3$*), 0.97 (t, 12H, $^3J_{HH}$ = 7.0 Hz, Dipp-Pr^i-C*H$_3$*), 1.12 (m, 18H, Dipp-Pr^i-C*H$_3$*), 1.23 (d, 6H, $^3J_{HH}$ = 7.0 Hz, Dipp-Pr^i-C*H$_3$*), 1.37 (d, 6H, $^3J_{HH}$ = 7.5 Hz, Si-Pr^i-C*H$_3$*), 1.50 (m, 2H, Si-Pr^i-C*H*), 1.63 (m, 2H, Si-Pr^i-C*H*), 2.26 (d, 4H, $^2J_{HP}$ = 13.9 Hz, Ph_2P-C*H$_2$*), 3.15 (hept, 2H, $^3J_{HH}$ = 6.7 Hz, Dipp-Pr^i-C*H*), 3.64 (m, 2H, Dipp-Pr^i-C*H*), 7.09 (m, 12H, Ar-C*H*), 7.76 (m, Ar, C*H*).

^{13}C{^{1}H} NMR (C_6D_6, 101 MHz, 298 K): δ = 12.3 (s, Ph_2P-*C*H_2), 14.0 and 16.7 (Si-Pr^i -*C*H_3), 18.2 and 18.5 (Si-Pr^i-*C*H), 18.6, 19.1, 20.5 and 20.8 (Si-Pr^i -*C*H_3), 22.8, 23.0, 23.8, 27.9 and 28.0 (Dipp-Pr^i-*C*H_3), 28.5 (Dipp-Pr-*C*H), 28.6 (Dipp-Pr^i-*C*H_3), 28.7 (Dipp-Pr-*C*H), 123.4, 123.8, 123.9, 124.8, 125.7, 128.6, 128.9, 129.3, 132.8, 144.8, 147.1, 148.1 (Ar-*C*).

^{31}P{^{1}H} NMR (C_6D_6, 162 MHz, 298 K): δ = -46.9 (s, *P*PhCH_2Si).

^{29}Si{^{1}H} NMR (C_6D_6, 99 MHz, 298 K): δ = -107.6 (s, *Si*Pr$^i_2$).

MS/LIFDI-HRMS found (calcd.) m/z: 968.3390 (968.3455) for [M], and 485.1693 (485.1723) for [M-^{PhiP}DippGePPh].

(^{PhiP}DippGe)$_2$, 7. After ^{PhiP}DippGeCl (500 mg, 0.84 mmol, 2.0 eq.) and [Mg_2(^{Mes}nacnac)$_2$] (301 mg, 0.42 mmol, 1.0 eq.) were placed at a Schlenk flask, 5 mL of toluene were added at -80 °C. The mixture was subsequently allowed to warm to ambient temperature, the colour gradually changing from yellow to dark brown. After stirring for 2 hours, all volatiles were removed *in vacuo* and the residue was extracted with pentane. Concentrating and storing the solution overnight at -35 °C, yielded **7** as brown crystals (302 mg, 0.27 mmol, 64 %).

^{1}H NMR (C_6D_6, 400 MHz, 298 K): (C_6D_6, 400 MHz, 298 K): 0.95 (br s, 12H, Si-Pr^i-C*H$_3$*), 1.12 (m, 24H, Si-Pr^i-C*H$_3$*, Dipp-Pr^i-C*H$_3$*), 1.19 (d, 12H, $^3J_{HH}$ = 7.0 Hz, Dipp-Pr^i-C*H$_3$*), 1.33 (m, 4H, –), 1.72 (m, 4H, -), 3.65 (m, 4H, Dipp-Pr^i-C*H*), 6.99 (br s, 12H, Ar-C*H*), 7.09 (m, 6H, Ar-C*H*), 7.32 (br s, 8H, Ar-C*H*).

^{13}C{^{1}H} NMR (C_6D_6, 101 MHz, 298 K): δ = 14.2 and 15.3 (s, Ph_2P-*C*H_2), 19.2 (Si-Pr^i-*C*H_3), 20.9 (Dipp-Pr^i-*C*H_3), 22.8 (Si-Pr^i-*C*H_3), 24.5 (Si-Pr^i-*C*H), 26.2 (Dipp-Pr^i-*C*H_3), 27.1 and 28.2 (Dipp-Pr-*C*H), 28.6 (Dipp-Pr^i-*C*H_3), 28.7 (Dipp-Pr-*C*H), 123.3, 124.2, 129.6, 133.6, 137.1, 146.4 (Ar-*C*).

^{31}P{^{1}H} NMR (C_6D_6, 162 MHz, 298 K): δ = 0.28 (br s, *P*Ph_2).

^{29}Si{^{1}H} NMR (THF-*d*$_8$, 99 MHz, 298 K): δ = 3.59 (s, *Si*Pr$^i_2$).

Anal. calcd. for $C_{62}H_{86}Ge_2N_2P_2Si_2$: C, 66.33%; H, 7.72%; N, 2.50%; found C, 66.36%; H, 7.77%; N, 2.46%.

MS/LIFDI-HRMS found (calcd.) m/z: 1122.4275 (1122.4237) for [M], and 562.2128 (562.2114) for [M-(^{PhiP}DippGe·)].

Reaction between (^{PhiP}DippGe)$_2$ (7) and [IPr·Co·(η^6-C_7H_8)][BAr$^F_4$] (3)

Compounds **7** (50 mg, 0,045 mmol, 1.0 eq.) and **3** (62.5 mg, 0.045 mmol, 1.0 eq.) were added to a Schlenk flask under Ar, and 2 mL of toluene were added. The brown mixture was stirred overnight, resulting in a dark green solution. After all volatiles were removed *in vacuo*, the remaining green residue was characterized by ^{1}H and ^{31}P{^{1}H} NMR spectroscopy, in both D_6-benzene and D_8-THF. This clearly evidenced the formation of the T-shaped cobaltogermylene **3**, in addition to (amido)(phosphide)- and (amido)(phenyl)-germylenes **5** and **6**. For comparative NMR spectra, see Figures S37-S40.

(^{PhiP}DippGe·Co·DMAP)·5, 8. Compound **2** (100 mg, 0.05 mmol, 1 eq.) and DMAP (6.5 mg, 0.05 mmol, 1 eq.) were added to a Schlenk flask, cooled to -80 °C, and toluene was added (20 mL). The green reaction mixture was stirred overnight, leading to a brown solution above a blue oil. The supernatant solution was carefully removed *via* cannula, and all volatiles were removed from the oil *in vacuo*. The remaining oily residue was redissolved in pentane (10 mL) and the solution was concentrated to ~3 mL. Storage of the solution for 3 few days at -35 °C led to formation of crystals of **8** suitable for X-ray diffraction, which were isolated by filtration and dried *in vacuo*, yielding a red-blue powder of **8** (13 mg, 43% based on Ge).

^{1}H NMR (THF-d_8, 400 MHz, 298 K): 0.43 (d, 3H, $^{3}J_{HH}$ = 7.3 Hz, Si-Pr^{i}-C*H₃*), 0.57 (d, 3H, $^{3}J_{HH}$ = 6.7 Hz, Dipp-Pr^{i}-CH_3), 0.64 (d, 3H, $^{3}J_{HH}$ = 7.2 Hz, Si-Pr^{i}-CH_3), 0.73 (m, 6H, Si-Pr^{i}-CH_3, Dipp-Pr^{i}-CH_3), 0.81 (d, 6H, $^{3}J_{HH}$ = 7.5 Hz, Si-Pr^{i}-CH_3), 0.95 (m, 5H, Dipp-Pr^{i}-CH_3, Si-Pr^{i}-C*H*), 1.02 (m, 8H, Si-Pr^{i}-CH_3, Si-Pr^{i}-C*H*), 1.12 (m, 6H, Si-Pr^{i}-CH_3, Dipp-Pr^{i}-CH_3), 1.20 (m, 6H, Si-Pr^{i}-CH_3, Dipp-Pr^{i}-CH_3), 1.30 (m, 6H, Dipp-Pr^{i}-CH_3), 2.98 (s, 6H, pyN(CH_3)$_2$), 3.08 (m, 2H, Dipp-Pr^{i}-C*H*), 3.34 (m, 2H, Dipp-Pr^{i}-C*H*), 6.46 (m, 2H, py-Ar-*H*), 6.64 (m, 2H, Ar-*H*), 6.81 (m, 6H, Ar-*H*), 6.89 (m, 2H, Ar-*H*), 6.96 (m, 3H, Ar-*H*), 7.09 (m, 2H, Ar-*H*), 7.27 (m, 7H, Ar-*H*), 8.06 (m, 2H, py-Ar-*H*).

^{31}P{^{1}H} NMR (THF-d_8, 162 MHz, 298 K): δ = 53.8 (d, $^{2}J_{PP}$ = 97.8 Hz *P*Ph), 34.2 (d, $^{2}J_{PP}$ = 112.4 Hz *P*Ph$_2$).

^{29}Si{^{1}H} NMR (THF-d_8, 99 MHz, 298 K): δ = -22.0 (s, *Si*Pr$^{i}_2$).

MS/LIFDI-HRMS found (calcd.) m/z: 1104.3067 (1104.3178) for [M-DMAP-BAr$^{F}_4$]$^+$.

N. B. The low solubility of **8** precluded the acquisition of meaningful ^{13}C NMR data for this compound.

NMR, MS, UV/vis, and IR spectra

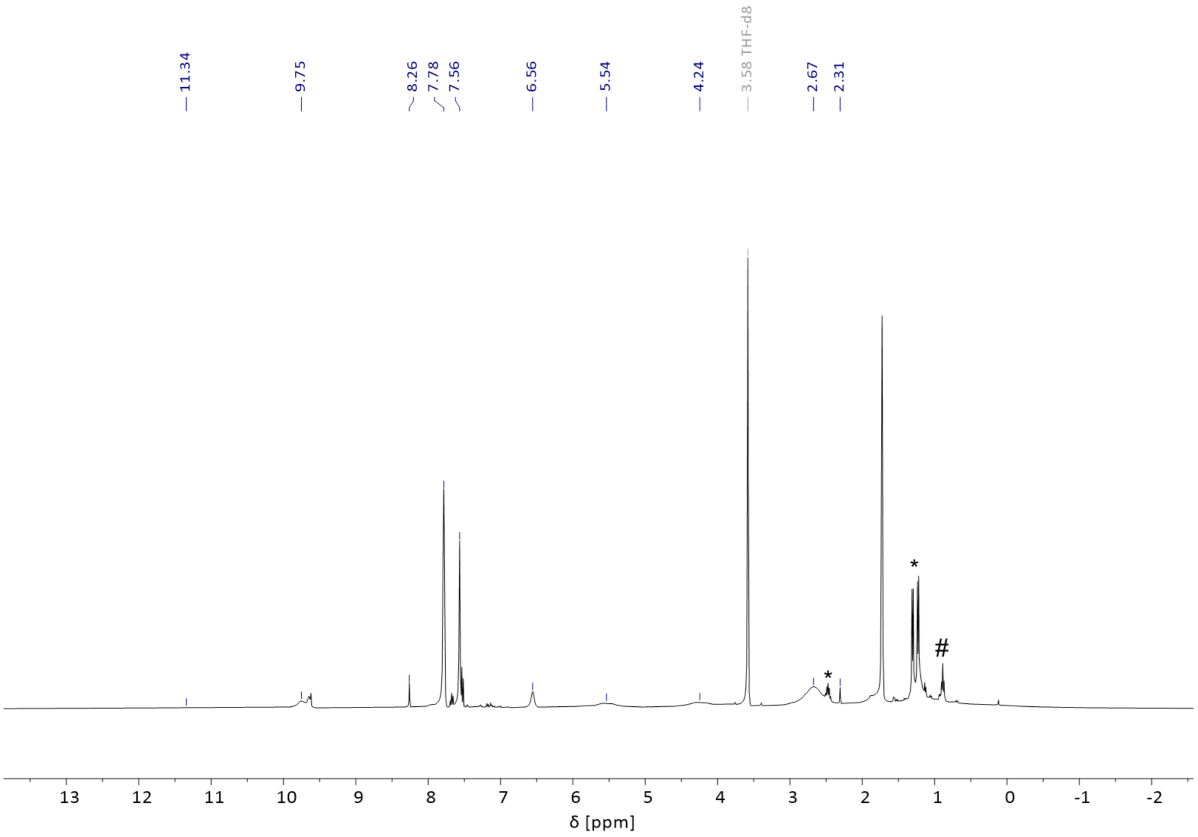

Figure S1. ^{1}H NMR spectrum of **2** as a solution in THF-d_8 at ambient temperature; $^{\#}$indicates residual pentane; *indicates small amounts of free ^{Dipp}NHC.

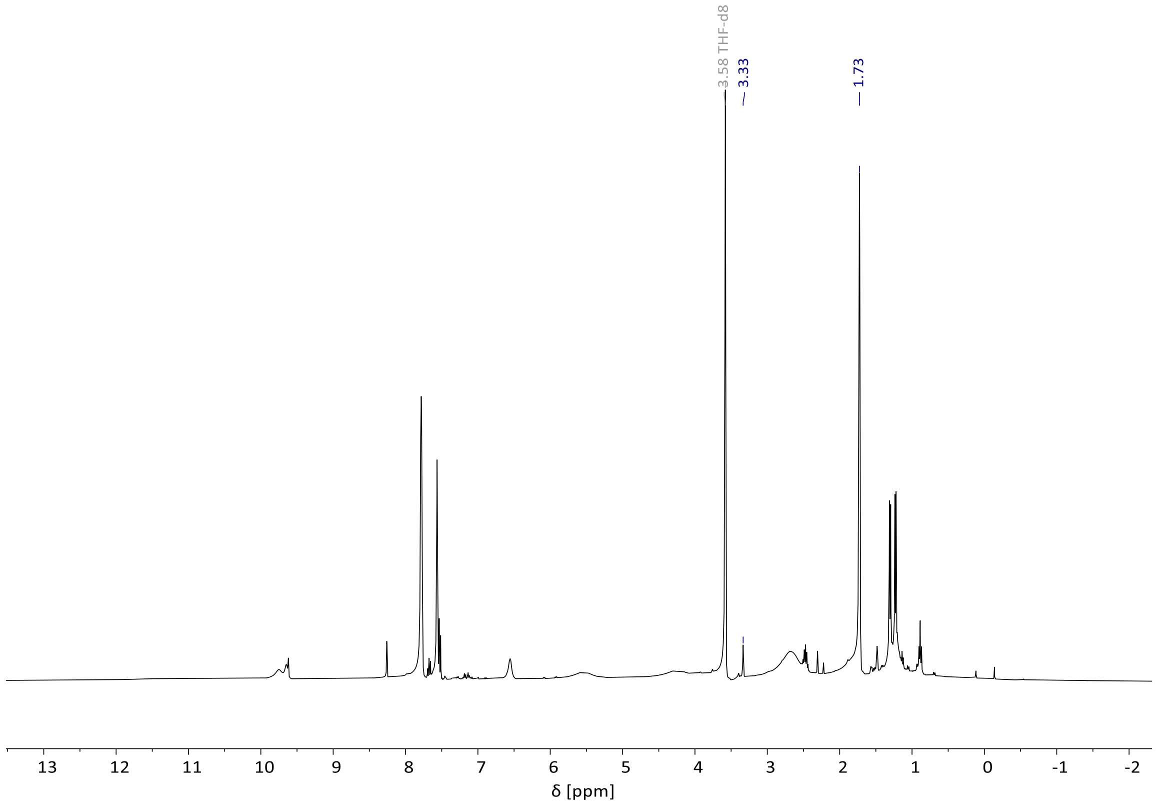

Figure S2. ^{1}H NMR spectrum of **2** as a solution in THF-d_8 at ambient temperature with a THF-d_8 capillary added.

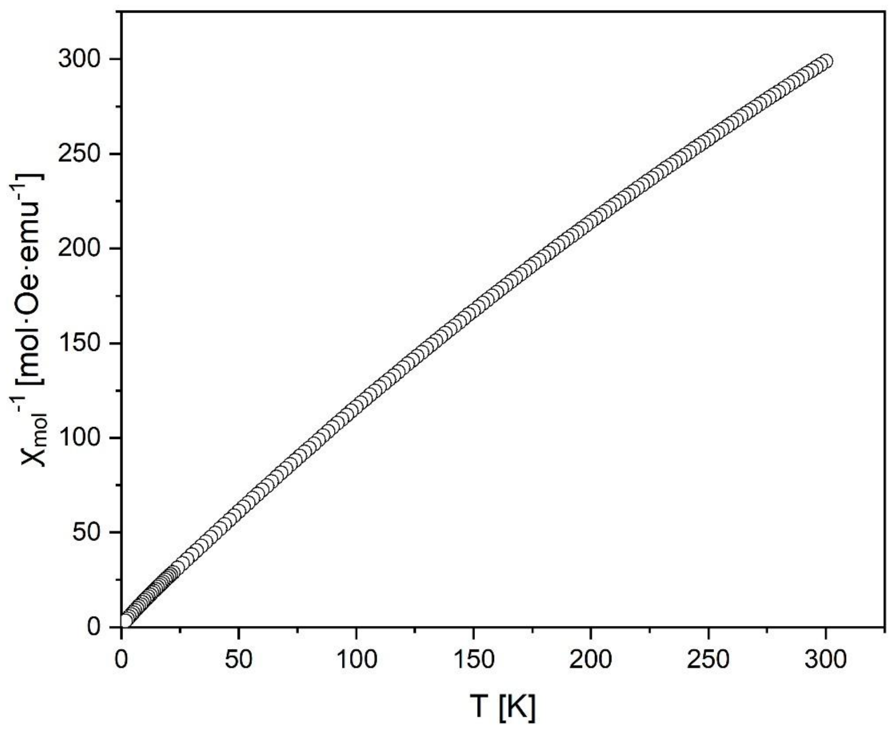

Figure S3. χ_{mol}^{-1} plotted against T for **2** from 1.8 K to 300 K.

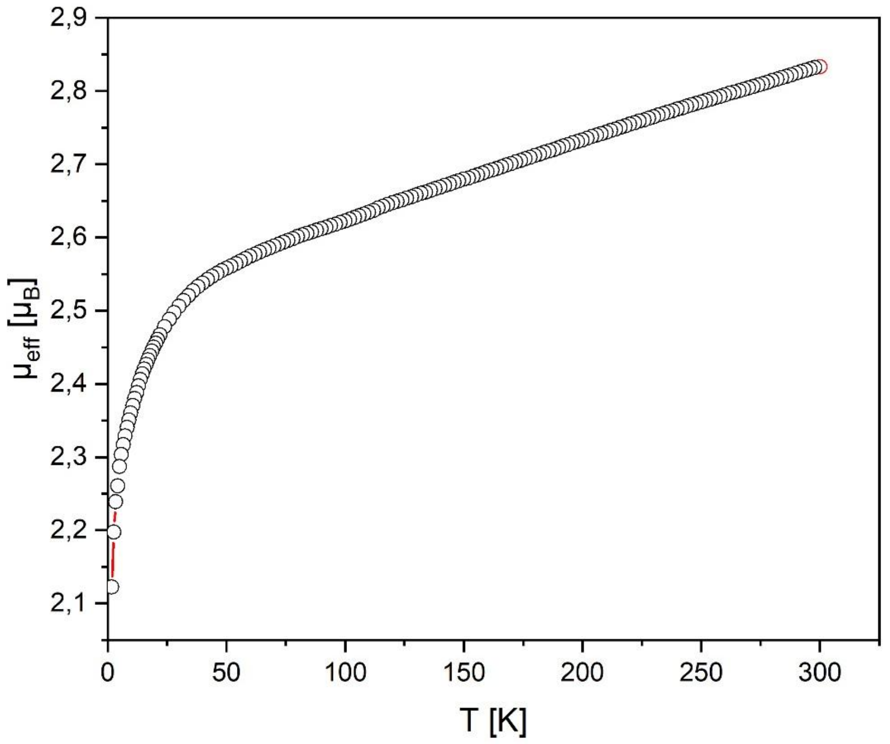

Figure S4. Effective magnetic moment μ_{eff} of crystalline sample of **2** from 1.8 K to 300 K.

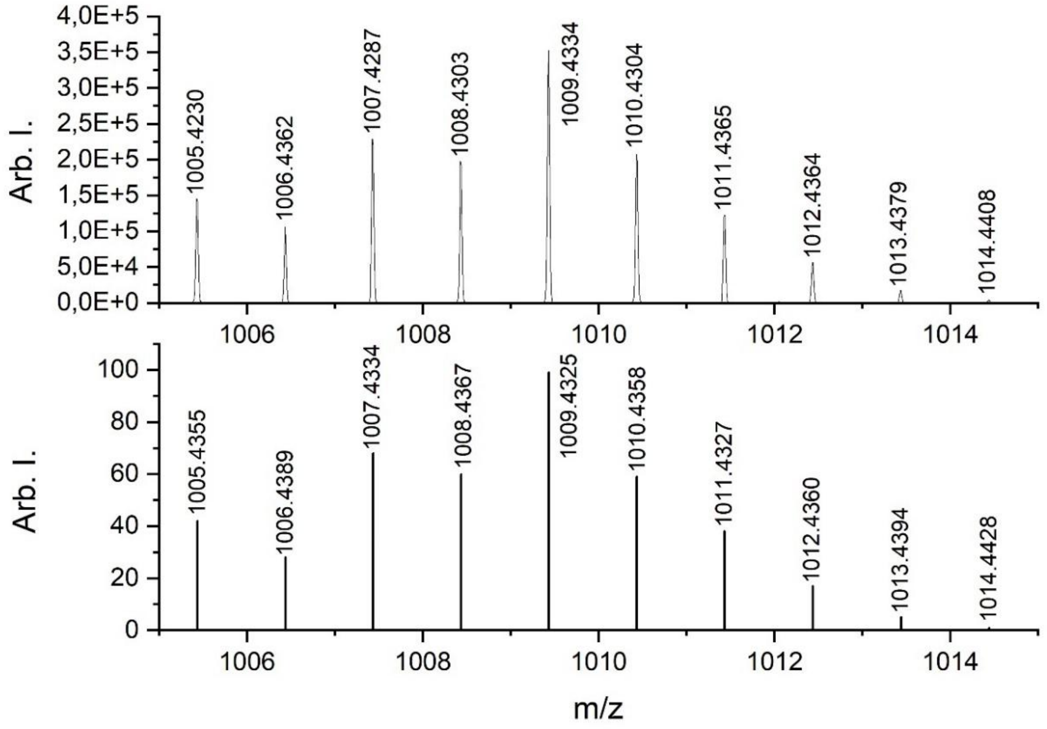

Figure S5. *Top:* Cutout from LIFDI/MS of **2;** *Bottom:* Calculated MS spectrum of [**2**-BAr_4^F]$^+$.

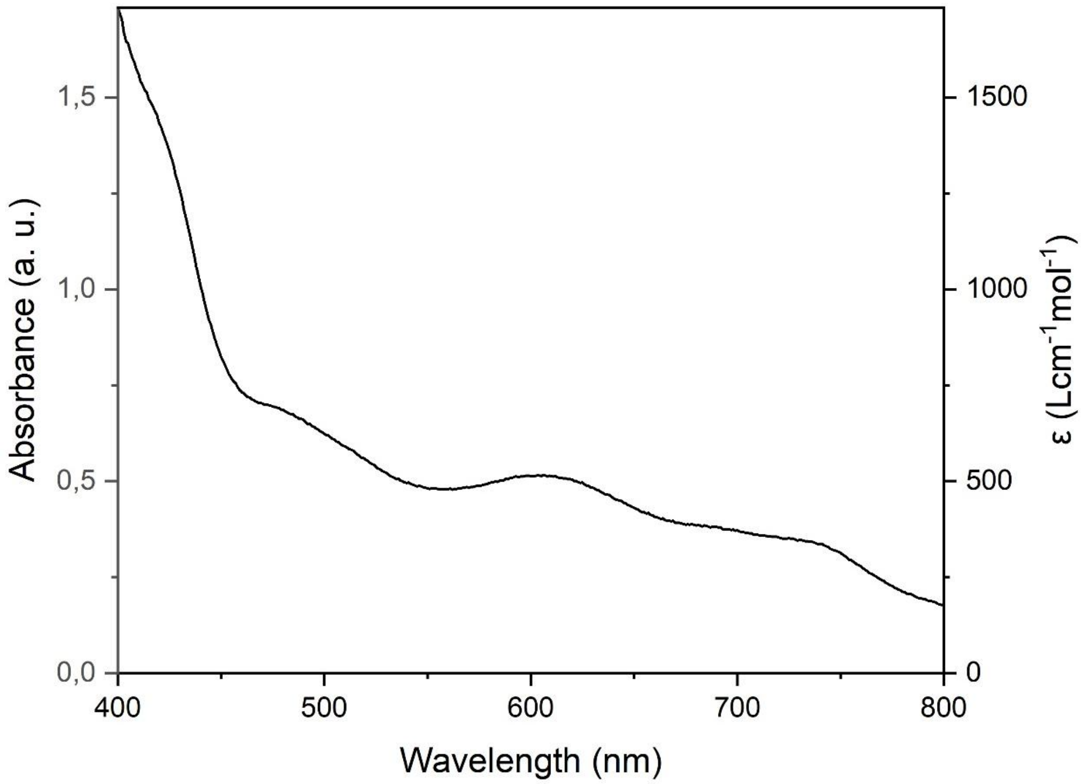

Figure S6. UV/vis spectrum of a 1.0x10^{-3} M solution of **2** in THF at ambient temperature.

Table S1. Simulated EPR parameters for **2**.

	x	*y*	*z*	iso
***g*-tensor**	1.9569	2.4210	2.4600	2.2793
$\mathbf{A_{59Co}}$	308	333	483	375
$\mathbf{A_{31P}}$	0	0	193	64

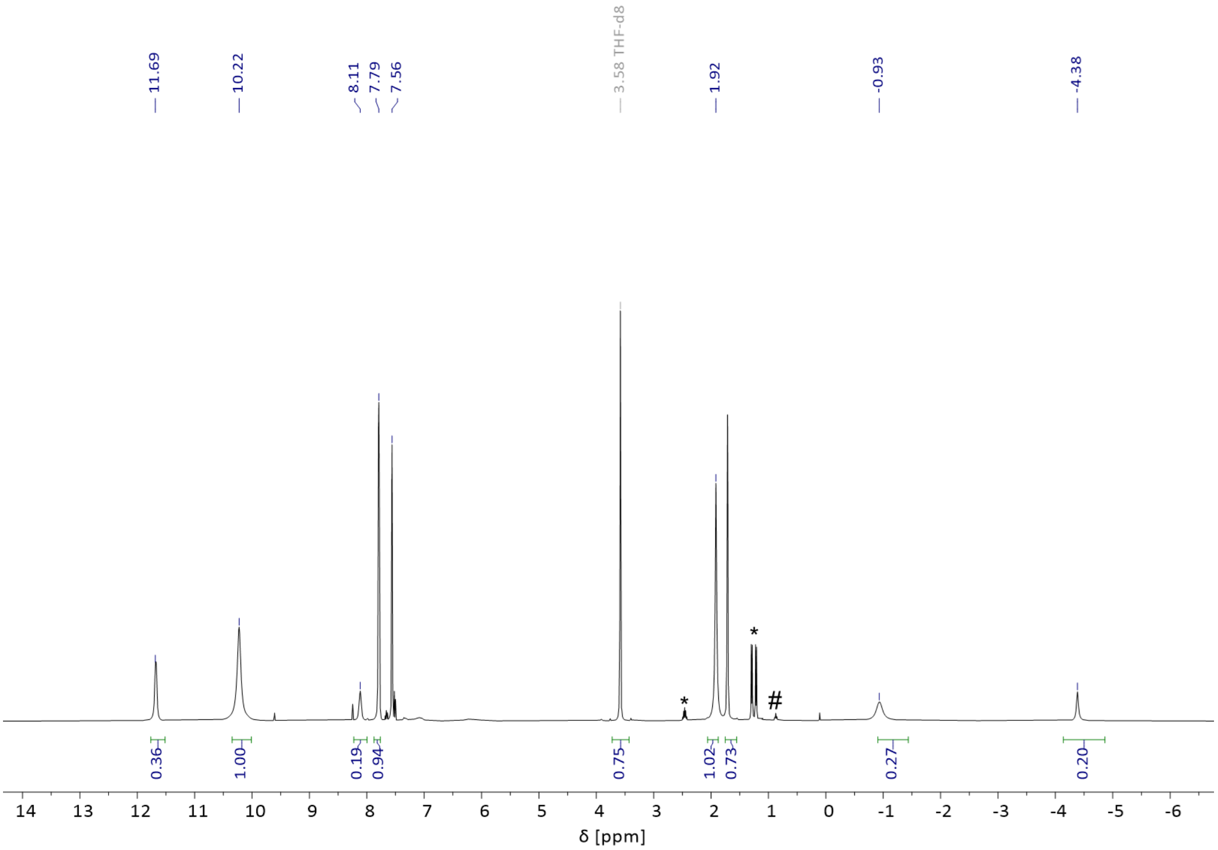

Figure S7. ^{1}H NMR spectrum of **3** as a solution in THF-d_8 at ambient temperature; *indicates small amounts of free ^{Dipp}NHC; $^{\#}$indicates small amounts of residual pentane.

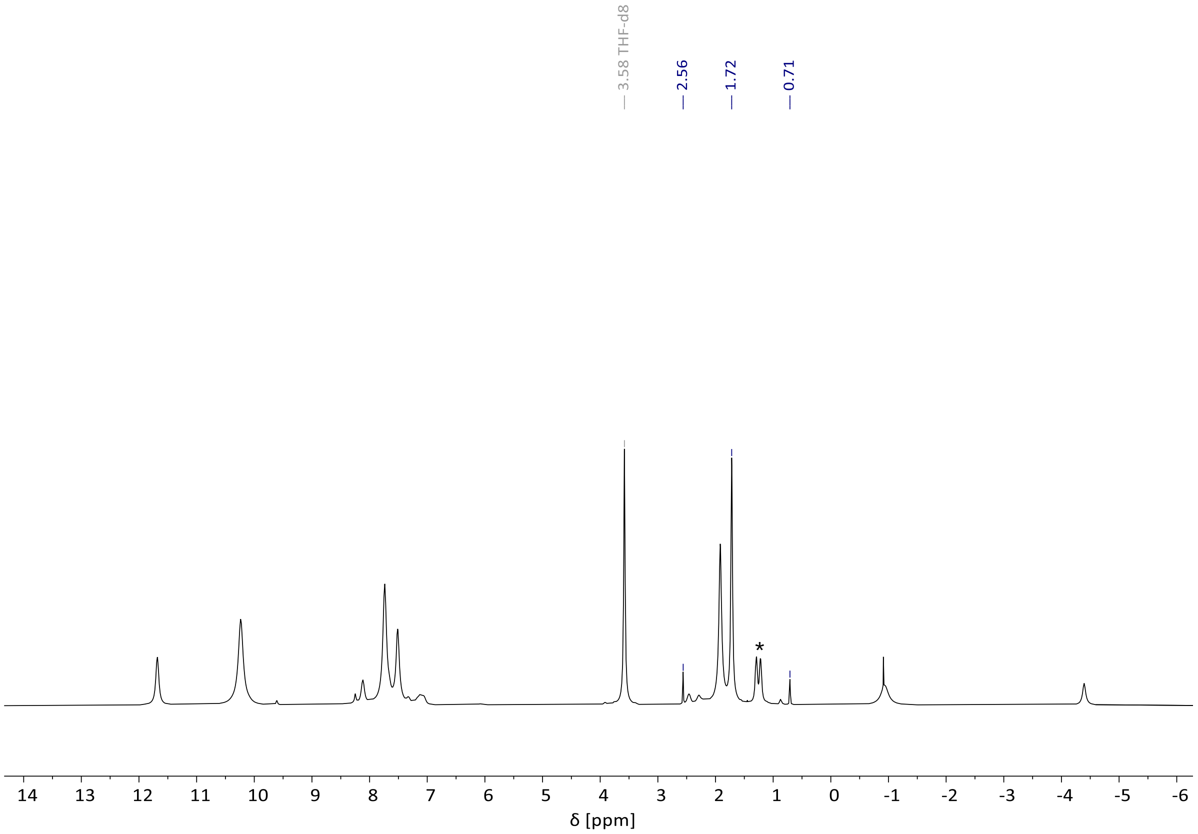

Figure S8. ^{1}H NMR spectrum of **3** as a solution in THF-d_8 at ambient temperature with a THF-d_8 capillary added; * indicates small amounts of free ^{Dipp}NHC.

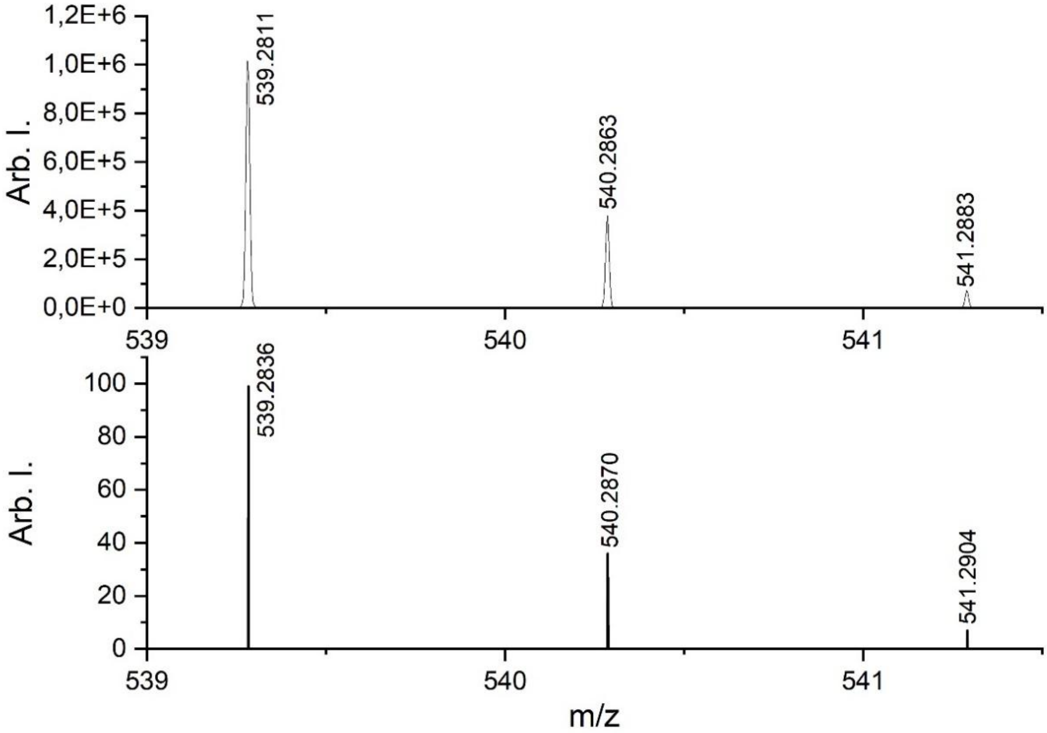

Figure S9. *Top:* Cutout from LIFDI/MS of **3;** *Bottom:* Calculated MS spectrum of [**3**-BAr_4^F]$^+$.

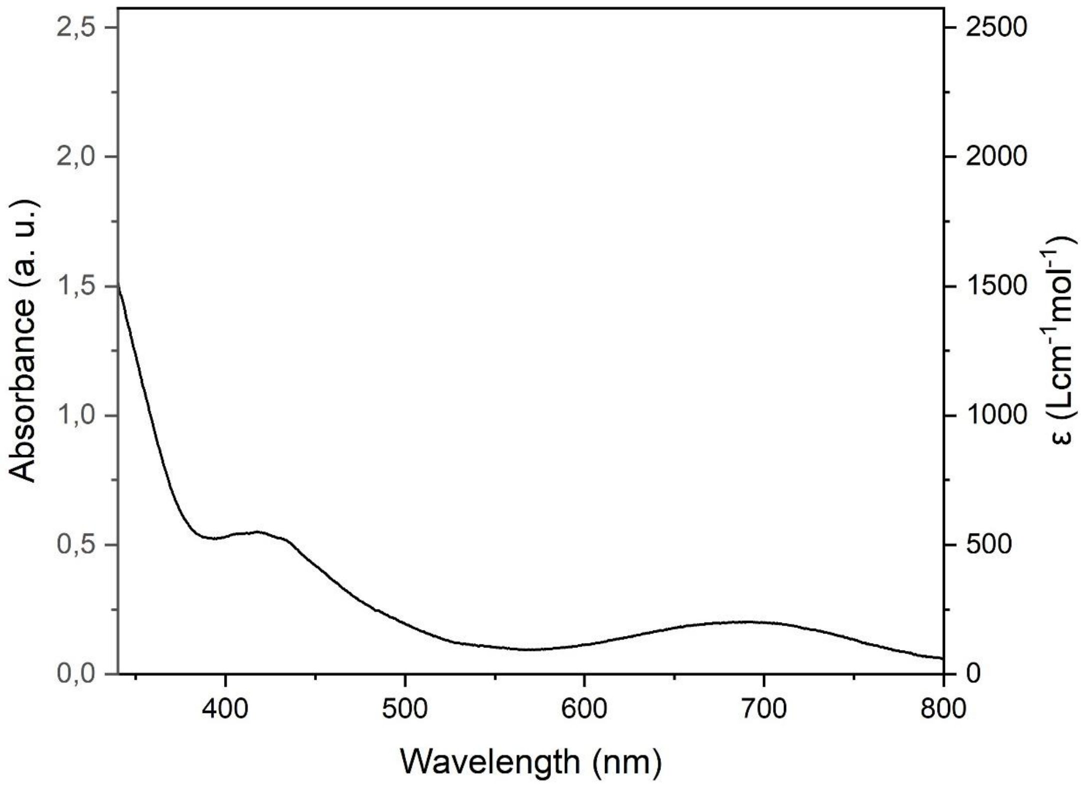

Figure S10. UV/vis spectrum of a 1.0x10^{-3} M solution of **3** in PhF at ambient temperature.

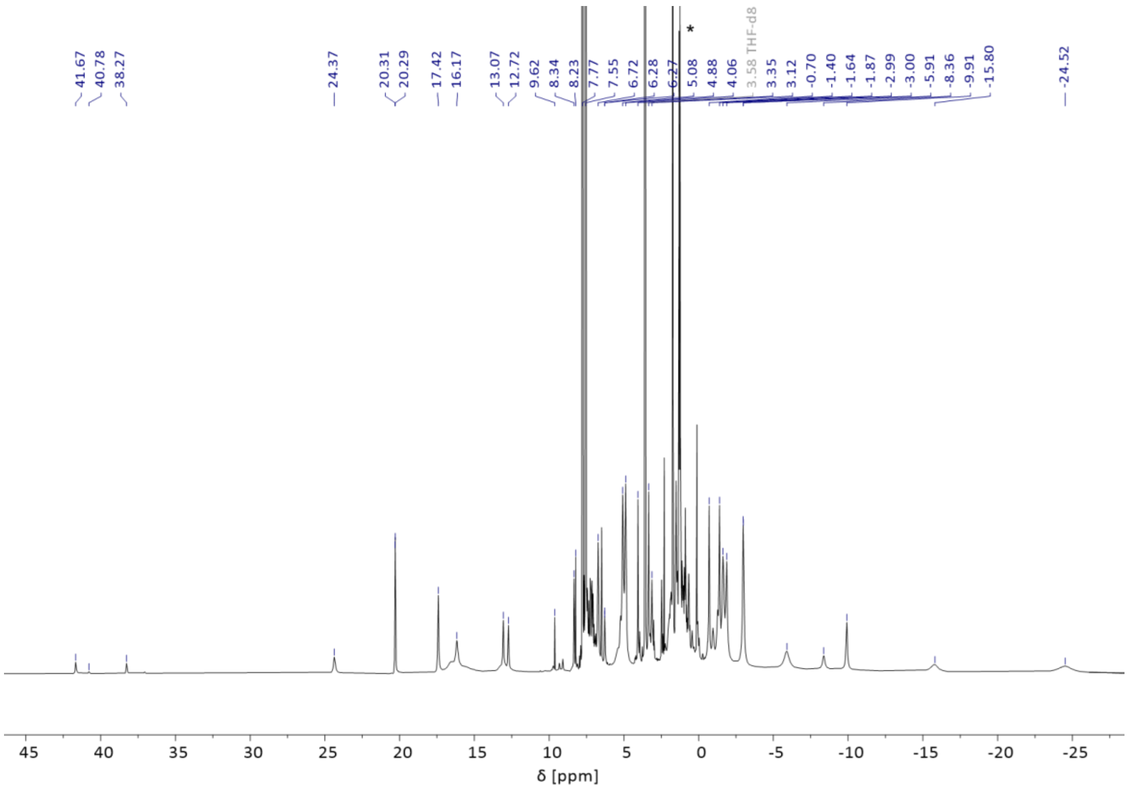

Figure S11. ^{1}H NMR spectrum of **4** as a solution in THF-d_8 at ambient temperature; * indicates small amounts of free ^{Dipp}NHC.

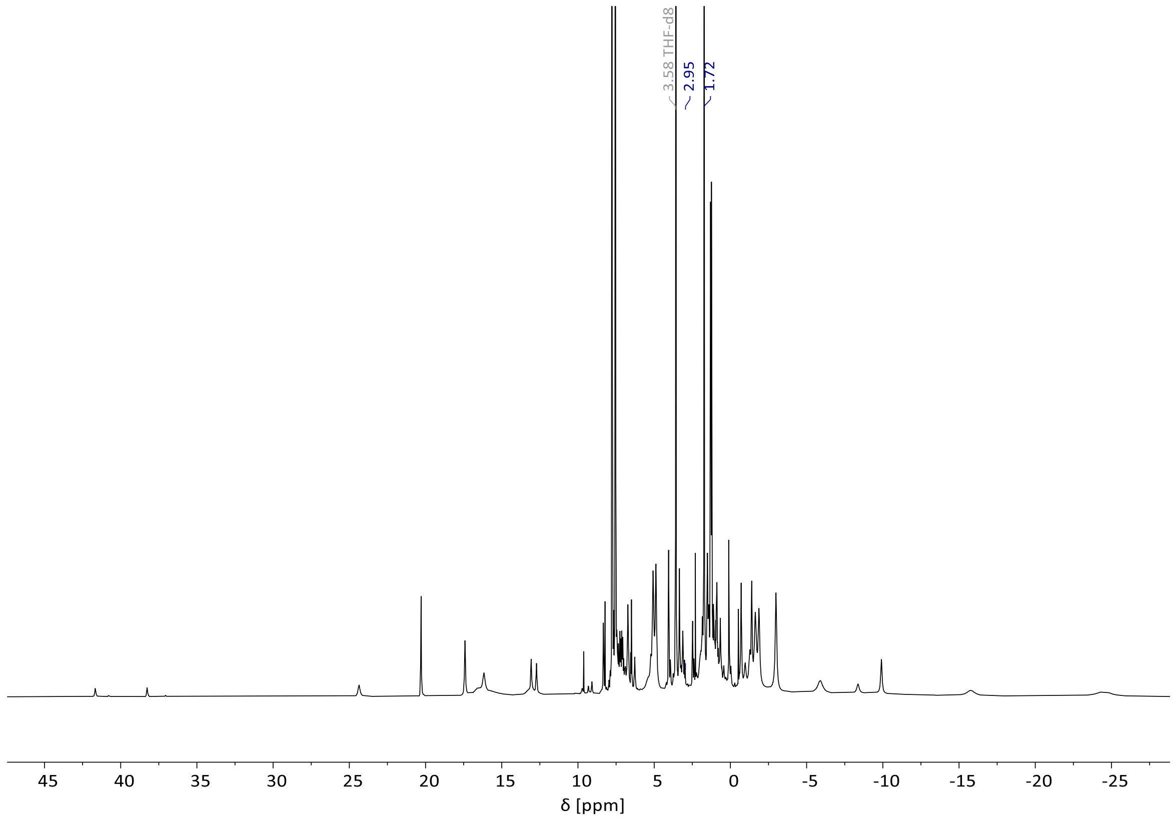

Figure S12. ^{1}H NMR spectrum of **4** as a solution in THF-d_8 at ambient temperature with a THF-d_8 capillary added.

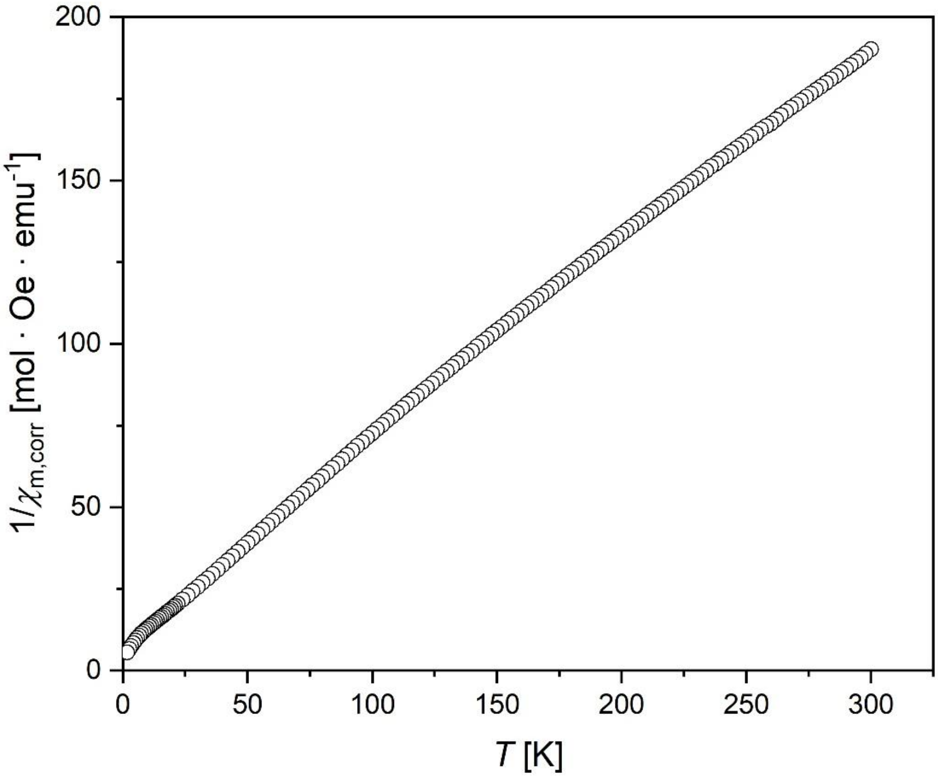

Figure S13. χ_{mol}^{-1} plotted against T for **4** from 1.8 K to 300 K.

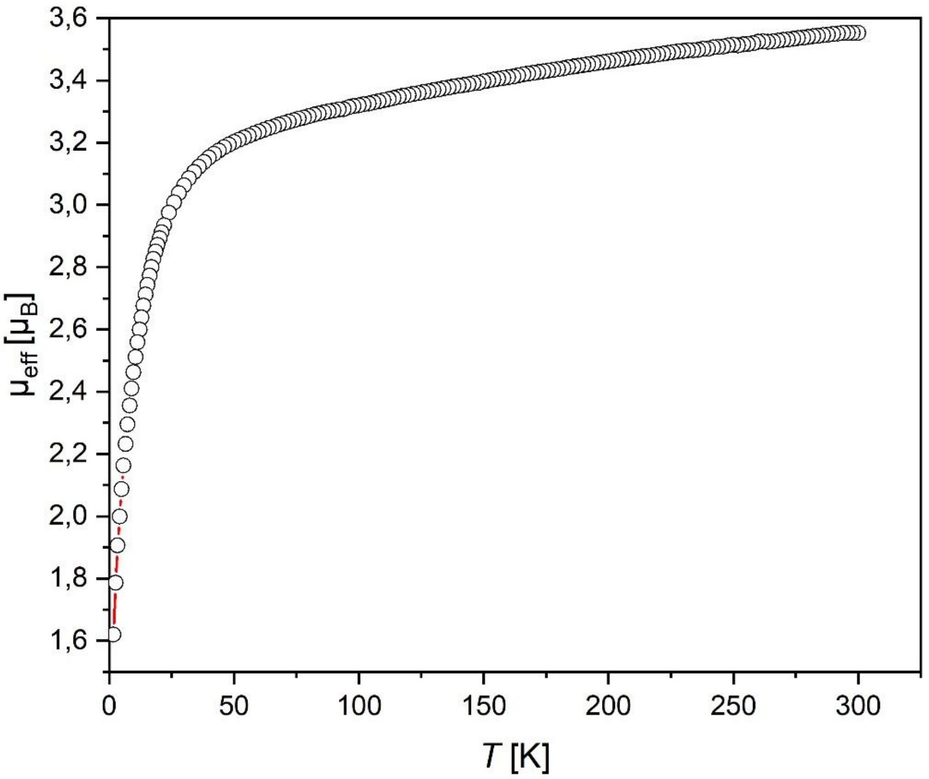

Figure S14. Effective magnetic moment μ_{eff} of crystalline sample of **4** from 1.8 K to 300 K.

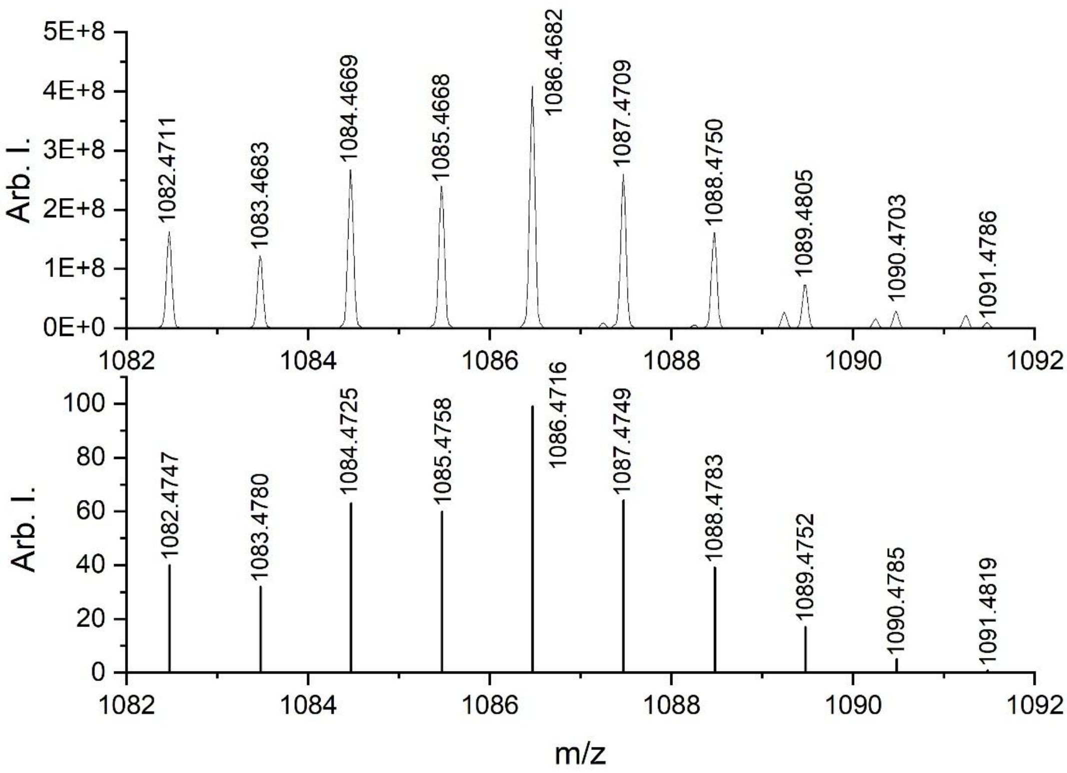

Figure S15. *Top:* Cutout from LIFDI/MS of **4;** *Bottom:* Calculated MS spectrum of [**4**-BAr_4^F]$^+$.

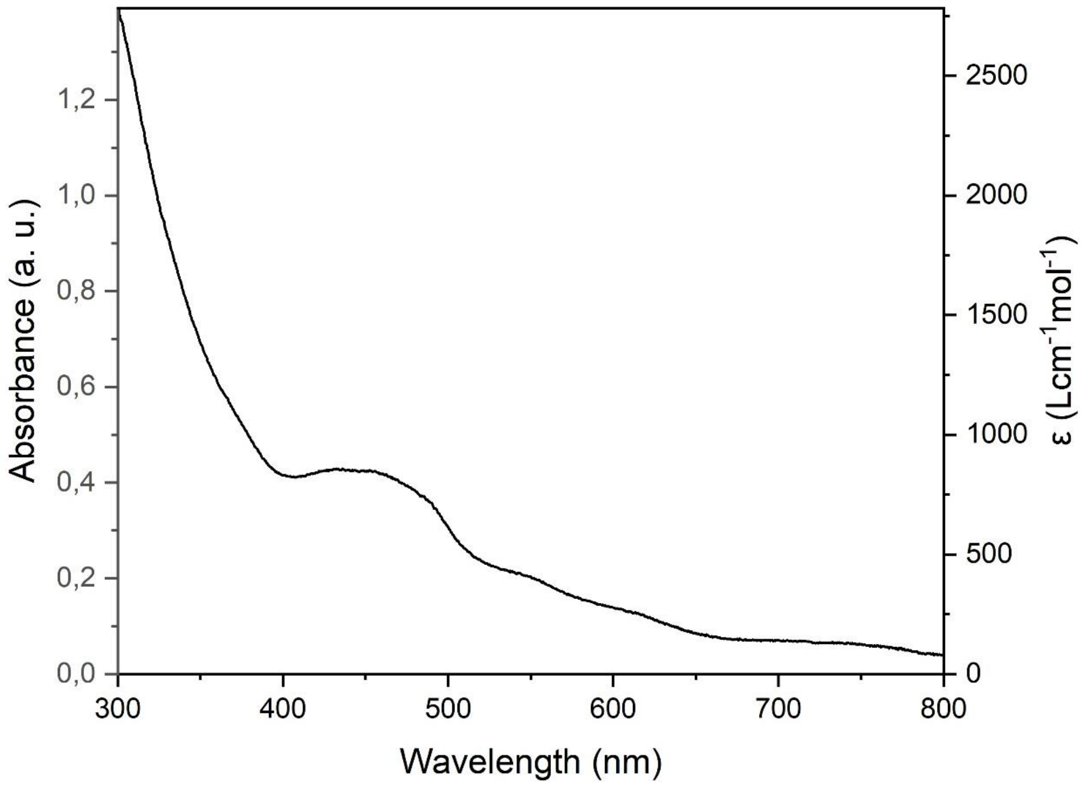

Figure S16. UV/vis spectrum of a 5.0x10^{-4} M solution of **4** in THF at ambient temperature.

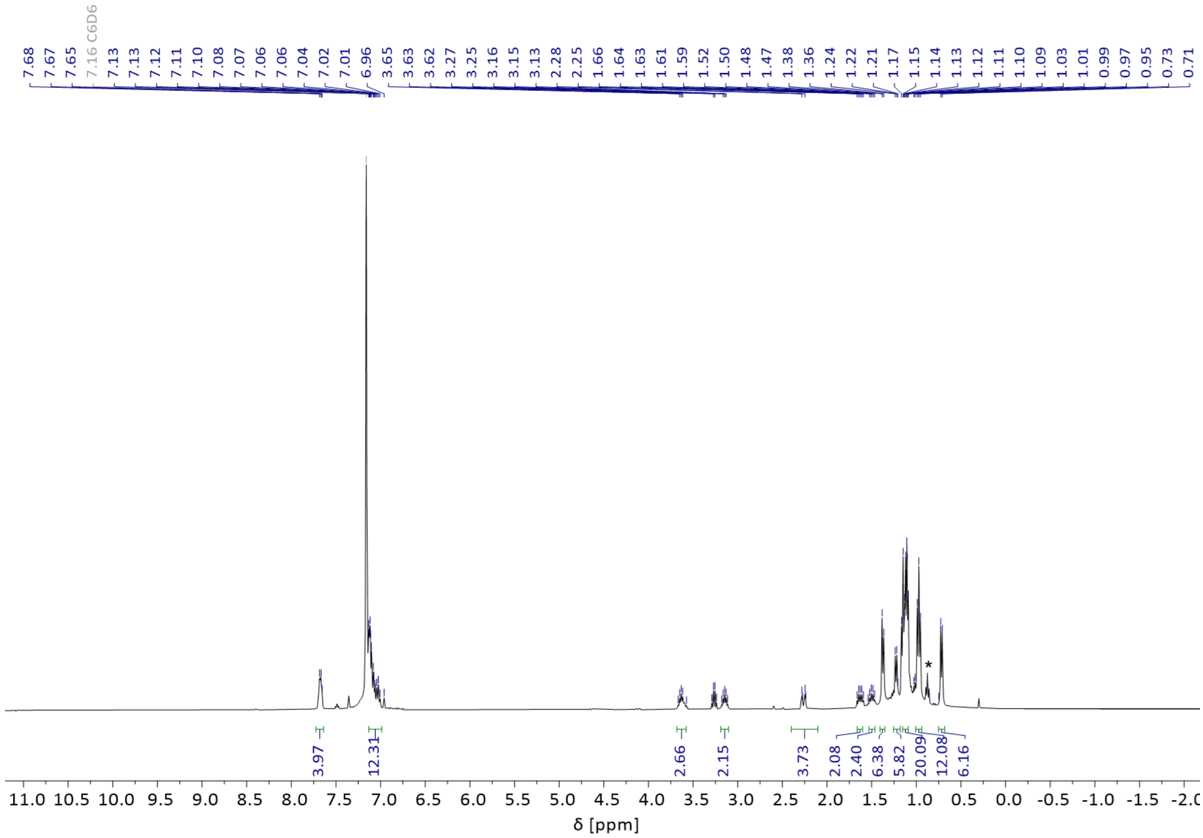

Figure S17. ^{1}H NMR spectrum of **5** as a solution in C_6D_6 at ambient temperature; * indicates small amounts of residual pentane.

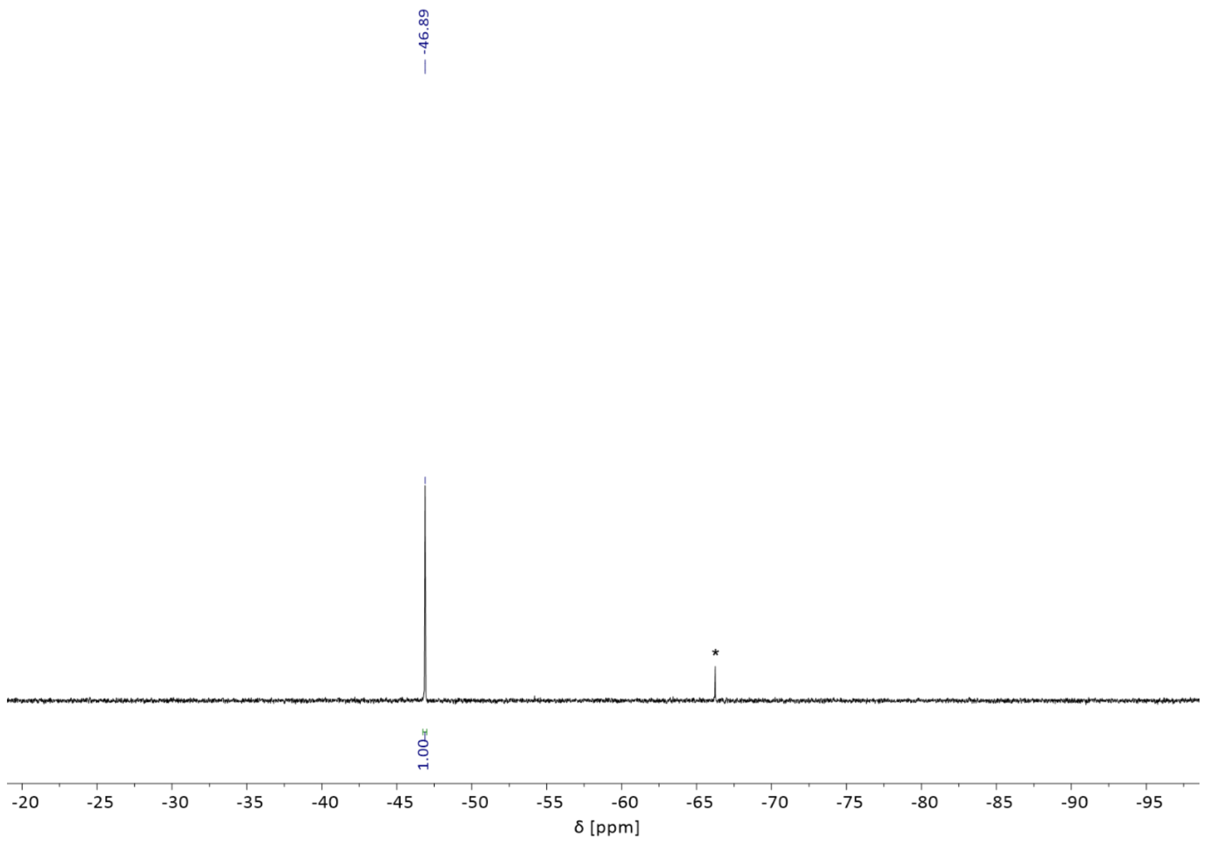

Figure S18. $^{31}P\{^1H\}$ NMR spectrum of **5** as a solution in C_6D_6 at ambient temperature; * indicates an unknown impurity.

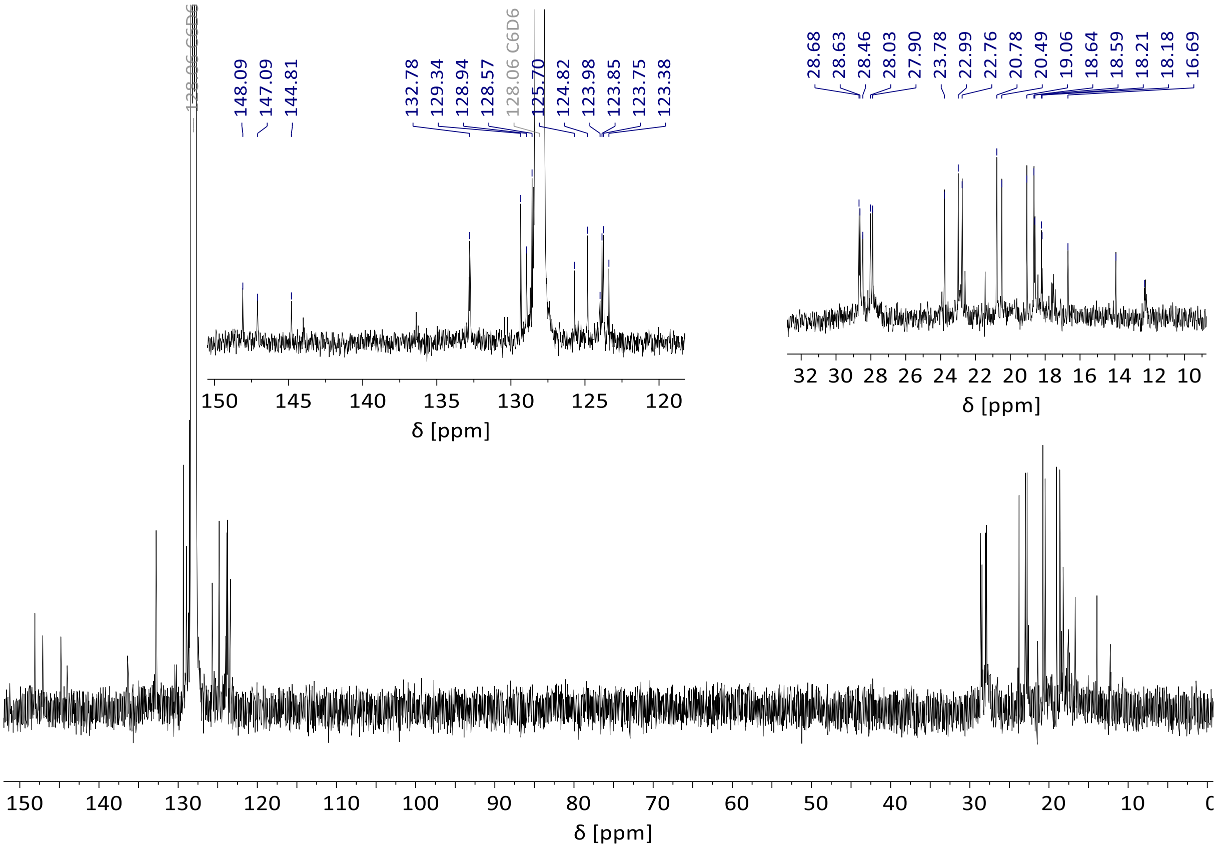

Figure S19. $^{13}C\{^1H\}$ NMR spectrum of **5** as a solution in C_6D_6 at ambient temperature.

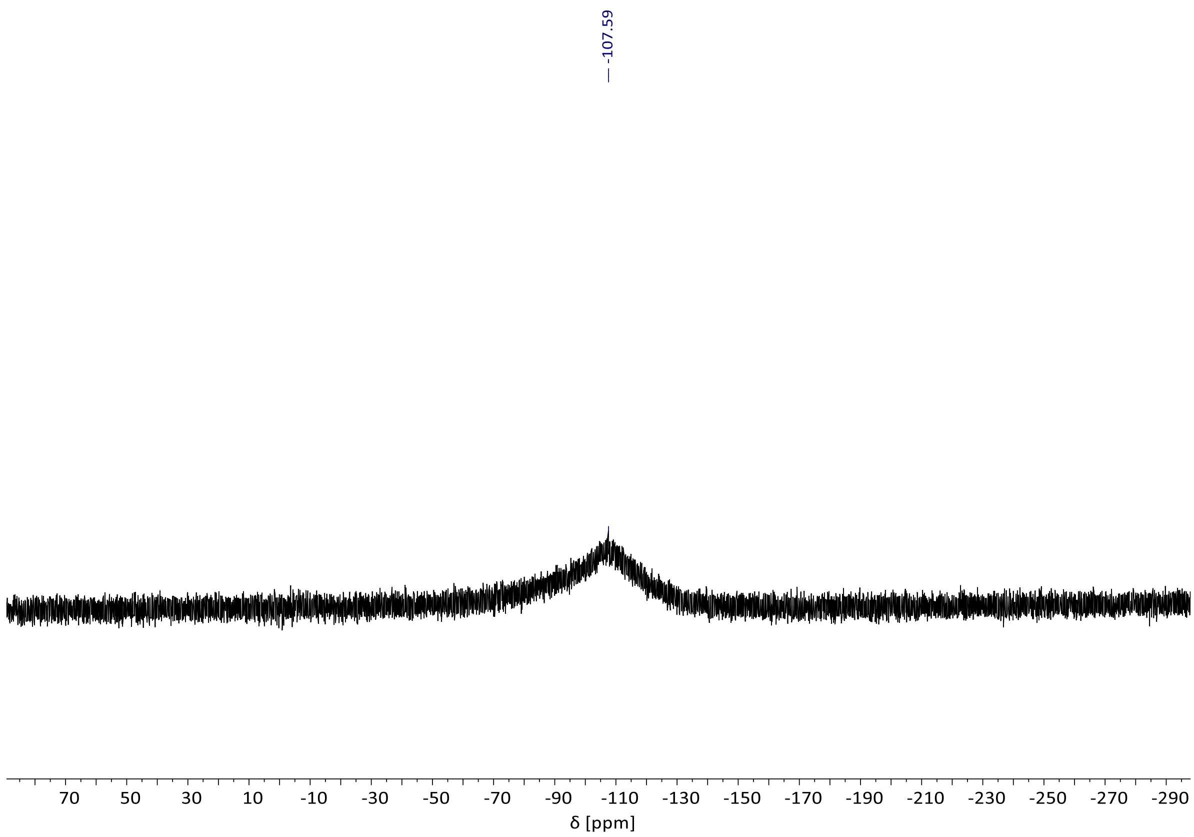

Figure S20. $^{29}Si\{^{1}H\}$ NMR spectrum of **5** as a solution in C_6D_6 at ambient temperature.

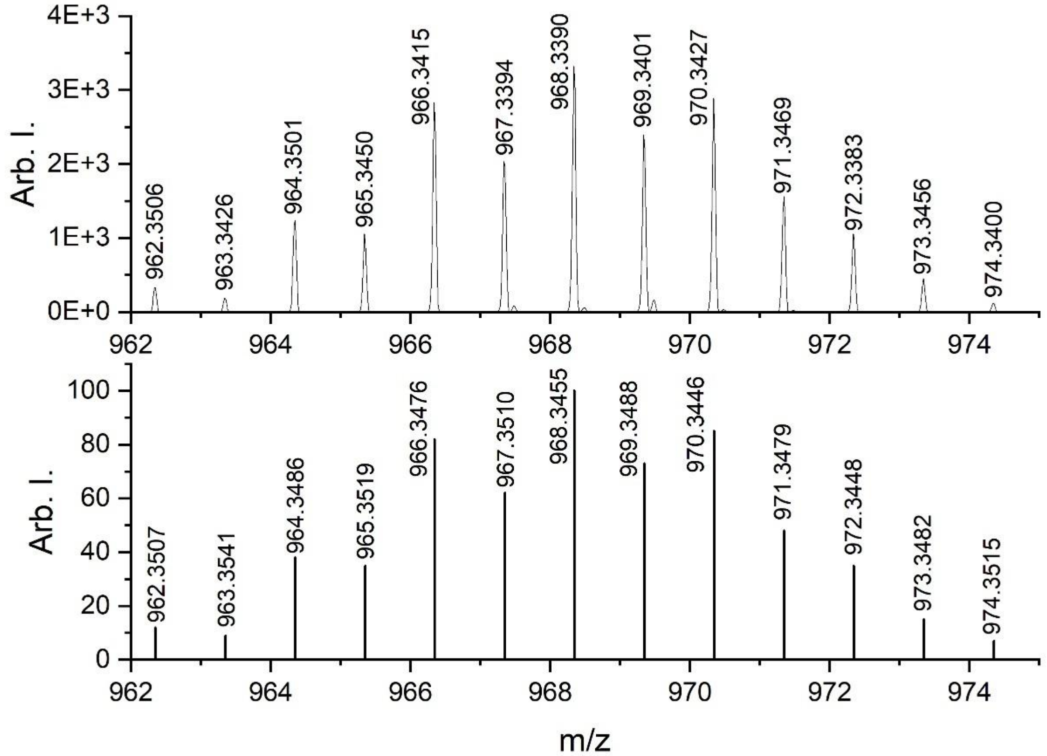

Figure S21. *Top:* Cutout from LIFDI/MS of **5;** *Bottom:* Calculated MS spectrum of [**5**].

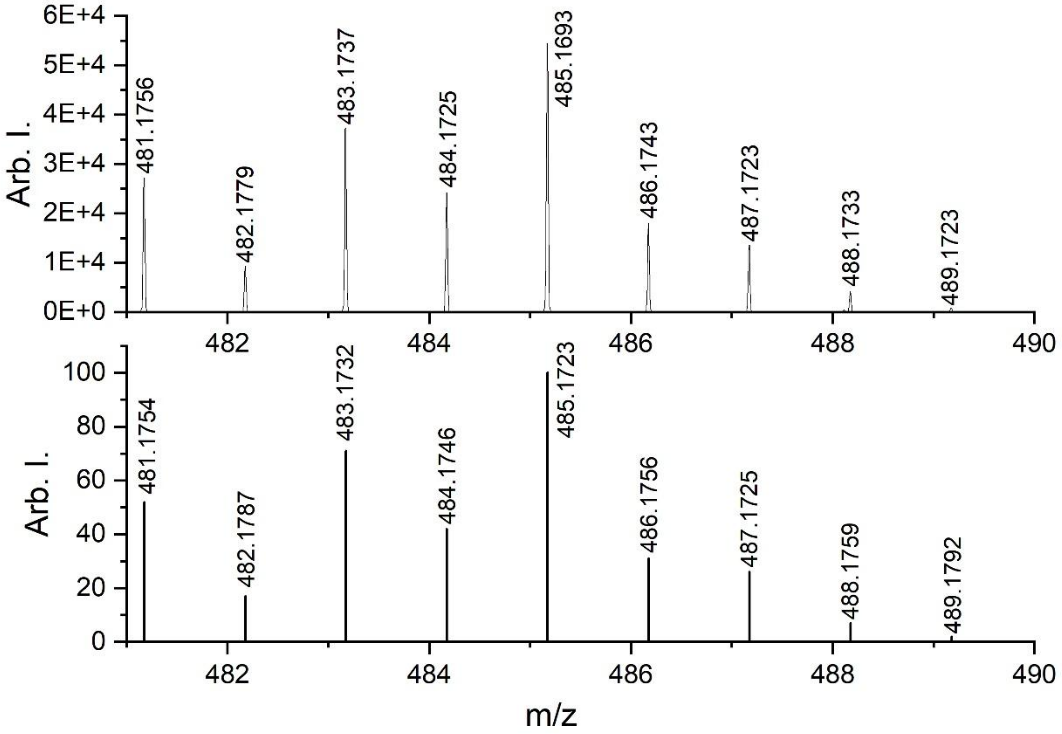

Figure S22. *Top:* Cutout from LIFDI/MS of **5;** *Bottom:* Calculated MS spectrum of the monomeric part in **5**, *i.e.* [^{iPPh}DippGePPh].

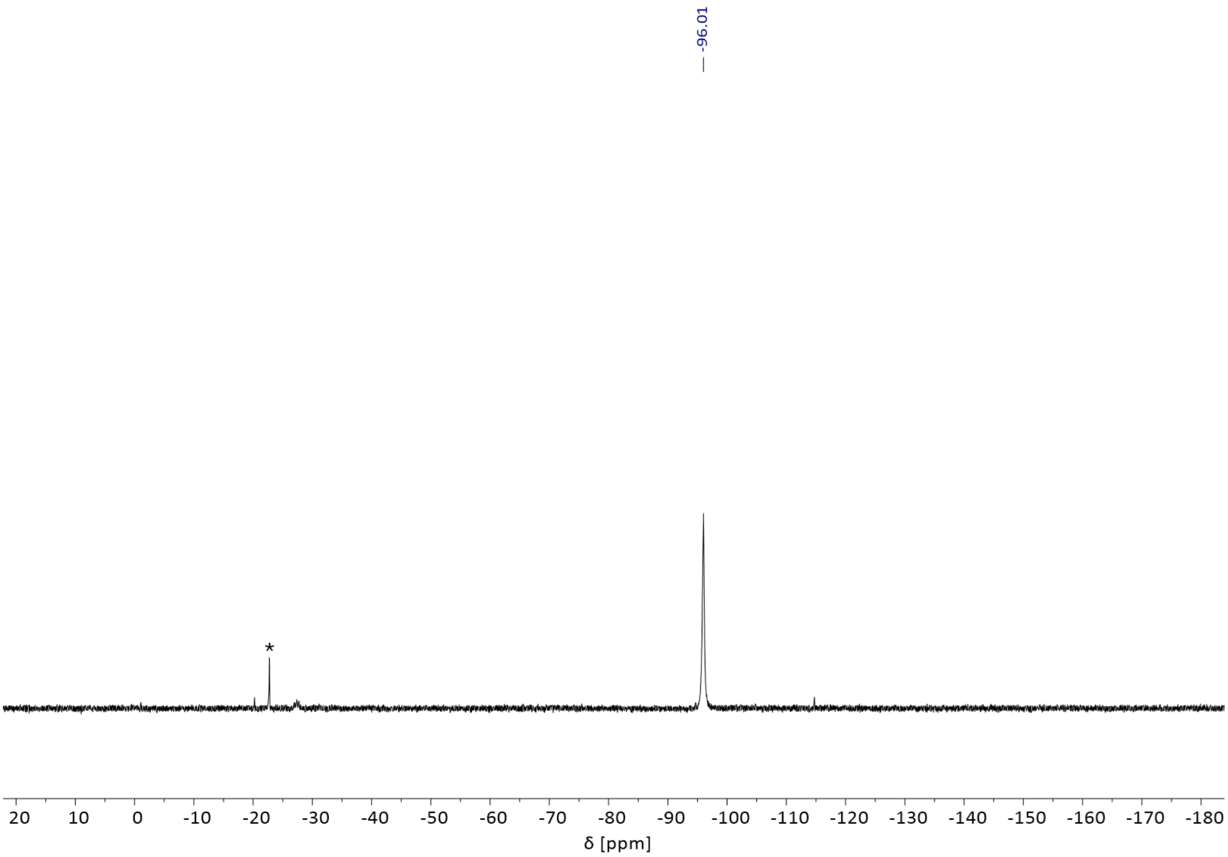

Figure S23. Crude $^{31}P\{^{1}H\}$ NMR spectrum of the first step in the direct synthesis of **5** from ^{PhiP}DippNH, in THF. * marks small amounts of unreacted ^{PhiP}DippNH.

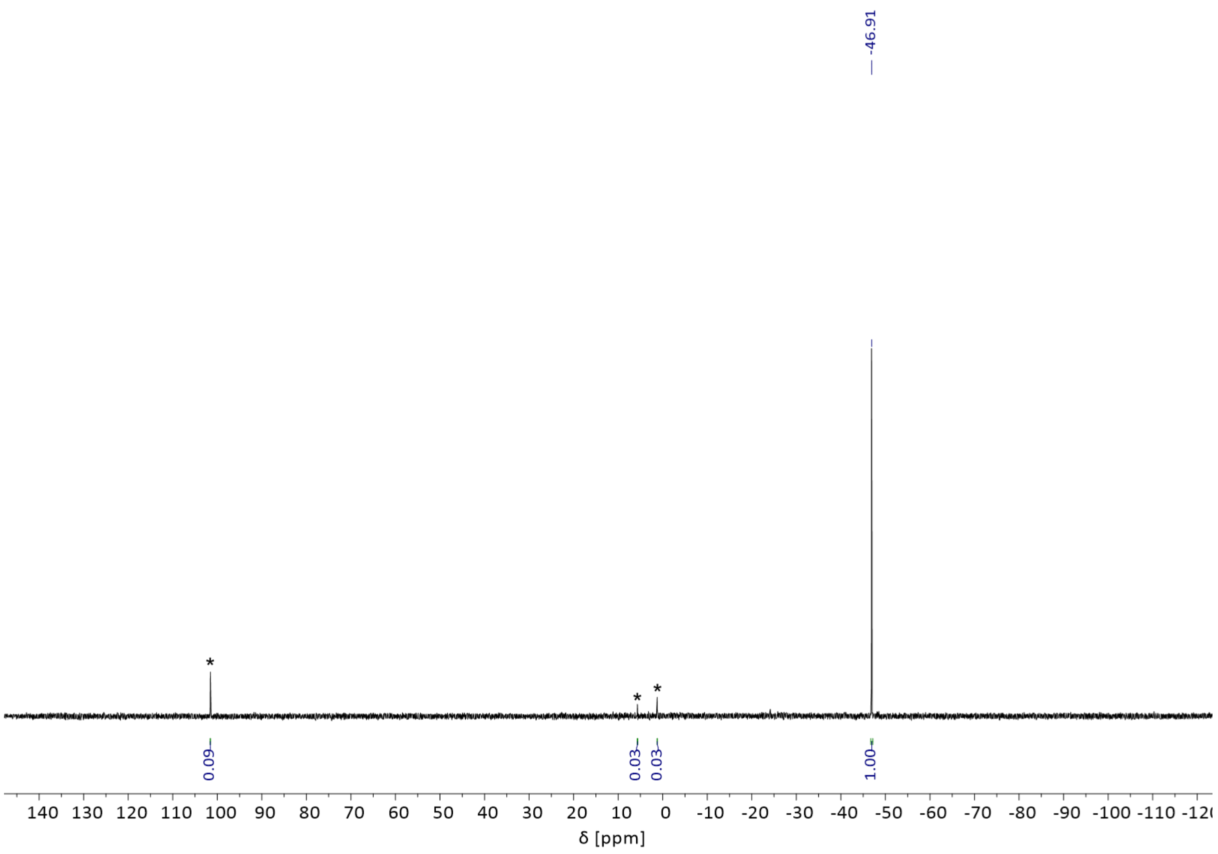

Figure S24. Crude $^{31}P\{^{1}H\}$ NMR spectrum of the second step in the direct synthesis of **5** in THF; *marks unknown impurities.

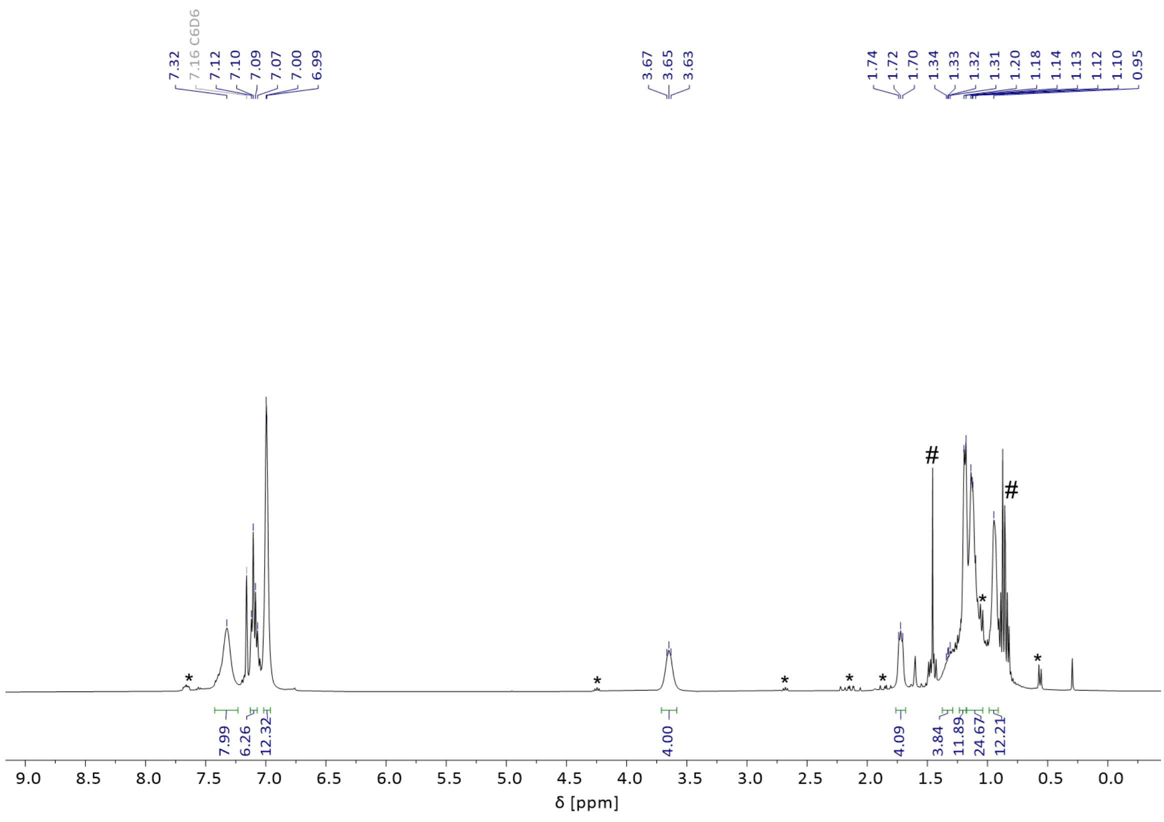

Figure S25. ^{1}H NMR spectrum of **7** as a solution in C_6D_6 at ambient temperature; * indicates small amounts of ^{PhiP}DippGeCl, while # marks impurities caused by paraffin oil.

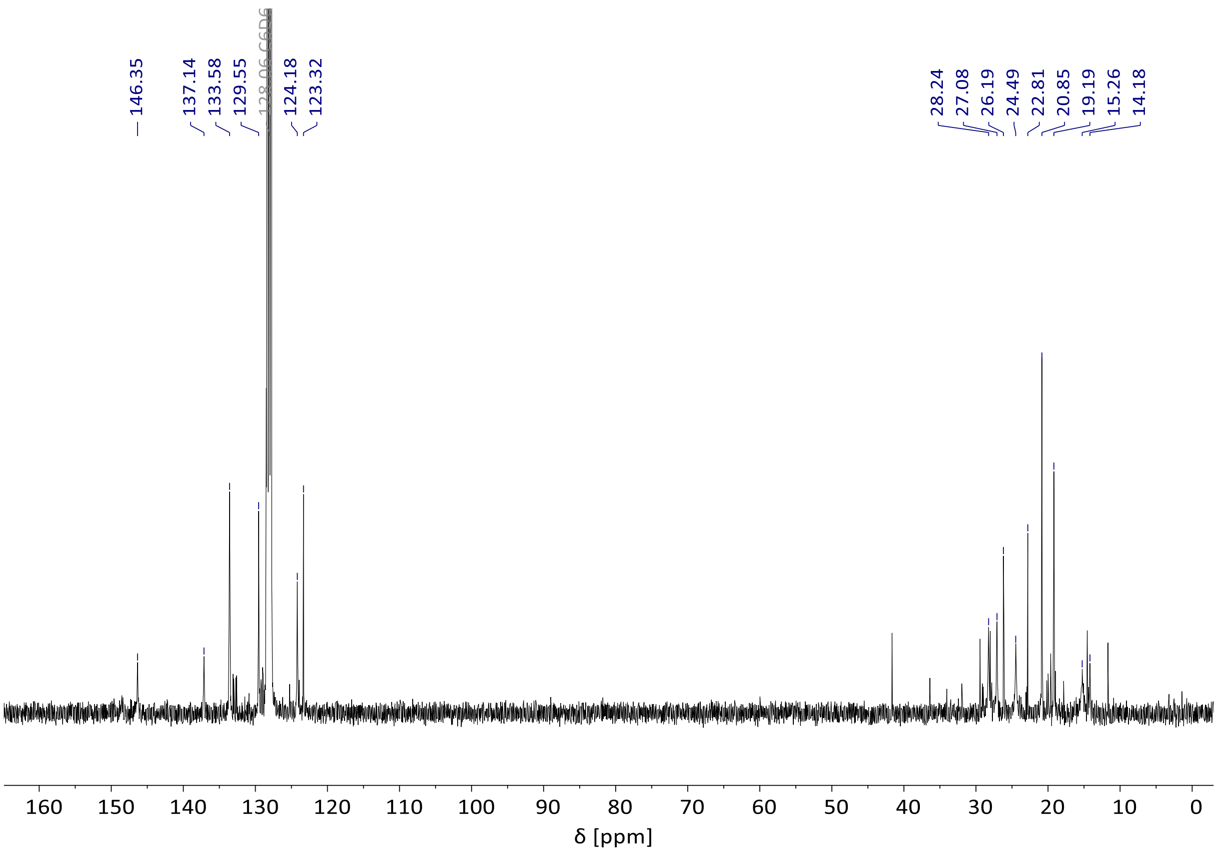

Figure S26. $^{13}C\{^{1}H\}$ NMR spectrum of **7** as a solution in C_6D_6 at ambient temperature.

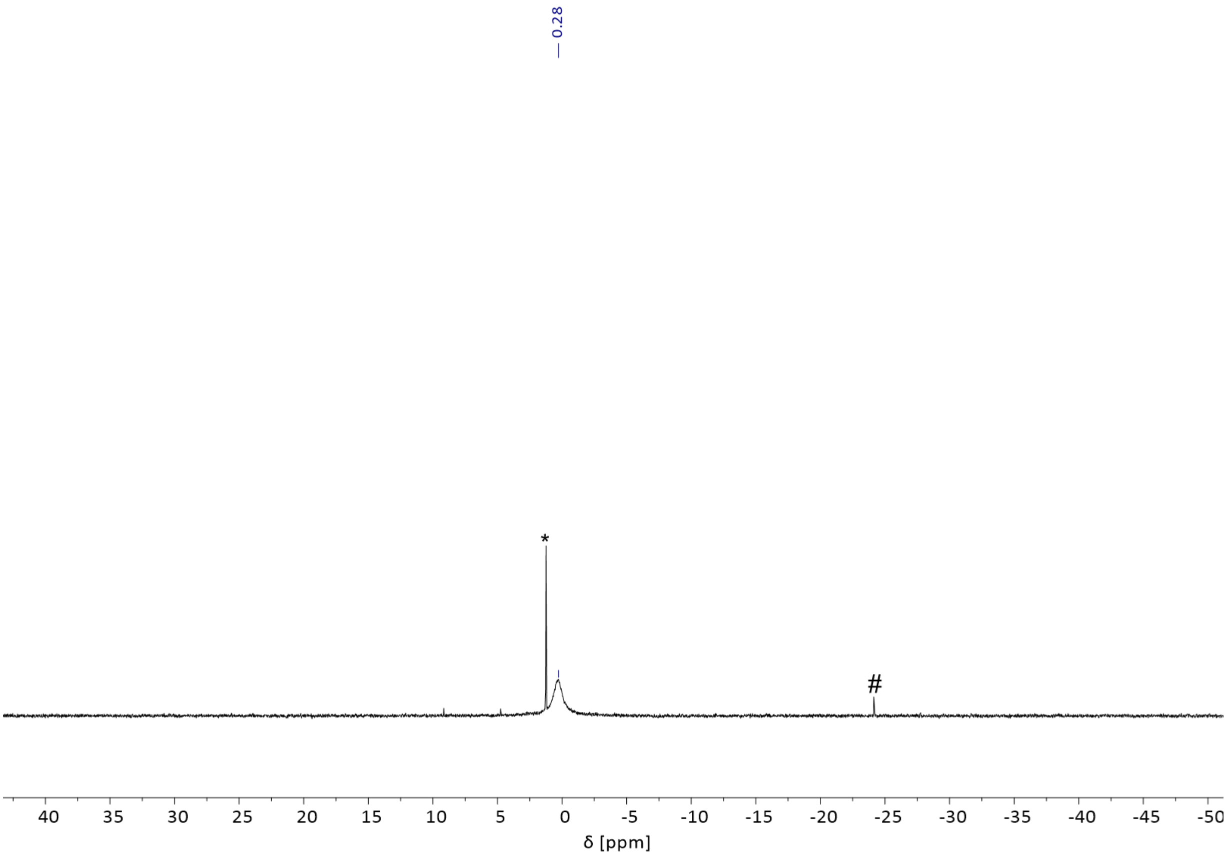

Figure S27. $^{31}P\{^{1}H\}$ NMR spectrum of **7** as a solution in C_6D_6 at ambient temperature; * indicates small amounts of ^{PhiP}DippGeCl, while # marks traces of ^{PhiP}DippNH.

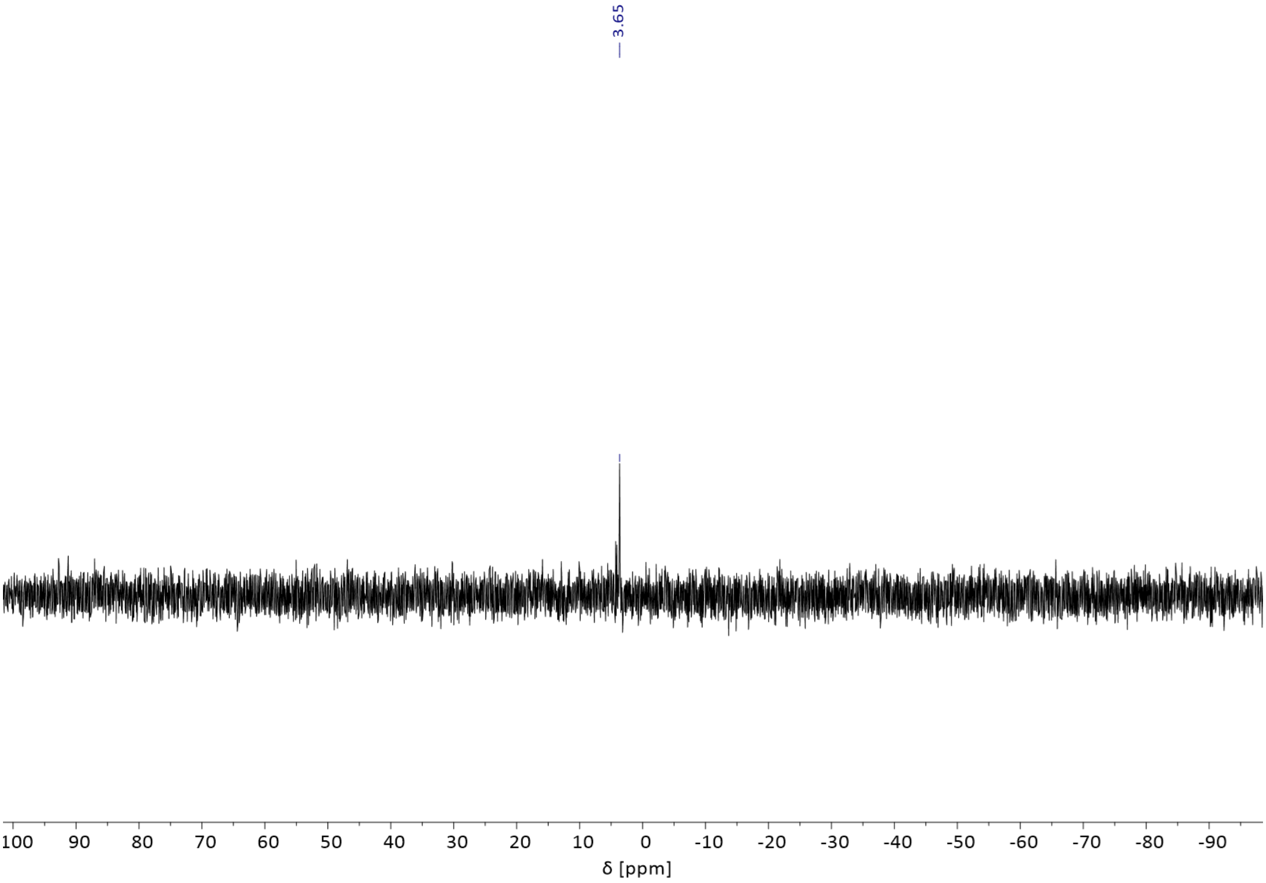

Figure S28. $^{29}Si\{^{1}H\}$ NMR spectrum of **7** as a solution in C_6D_6 at ambient temperature.

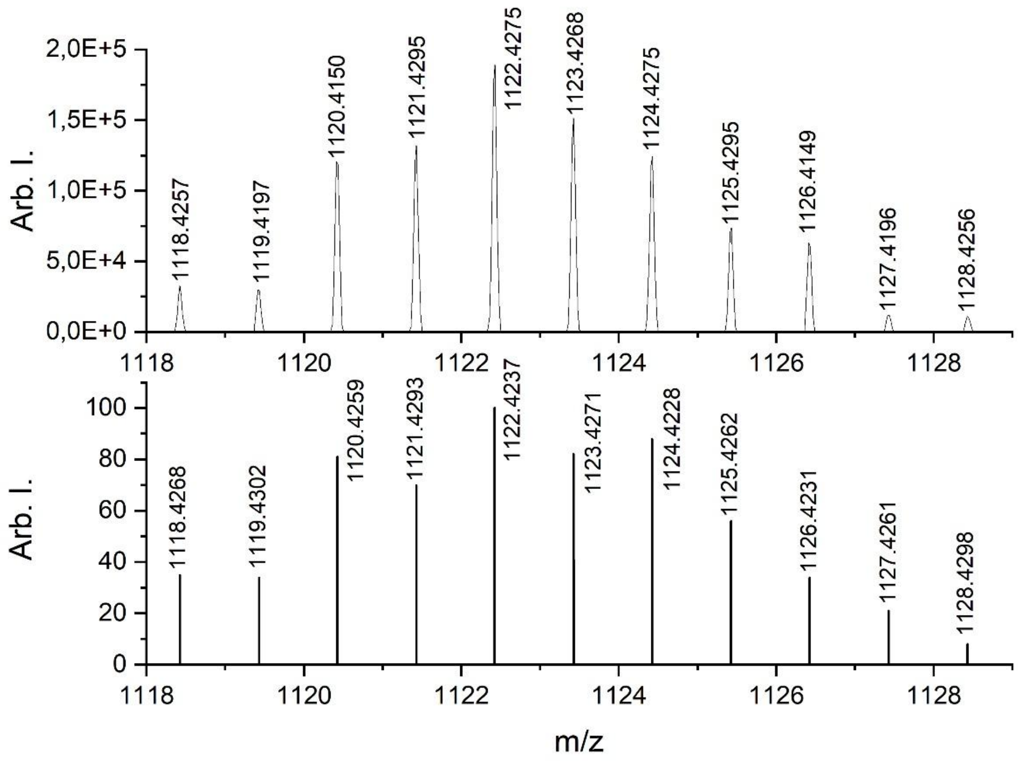

Figure S29. *Top:* Cutout from LIFDI/MS of **7;** *Bottom:* Calculated MS spectrum of **7**.

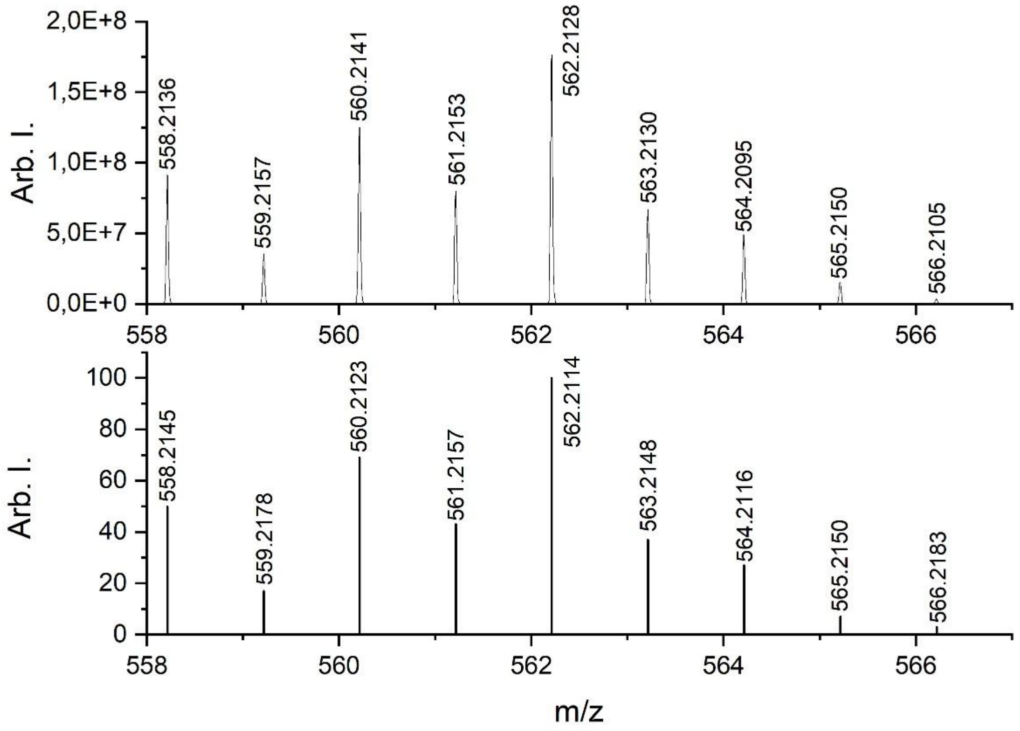

Figure S30. *Top:* Cutout from LIFDI/MS of **7**; *Bottom:* Calculated MS spectrum of [^{PhiP}DippGe·], *i.e.* the homolytically cleaved monomeric form of **7**.

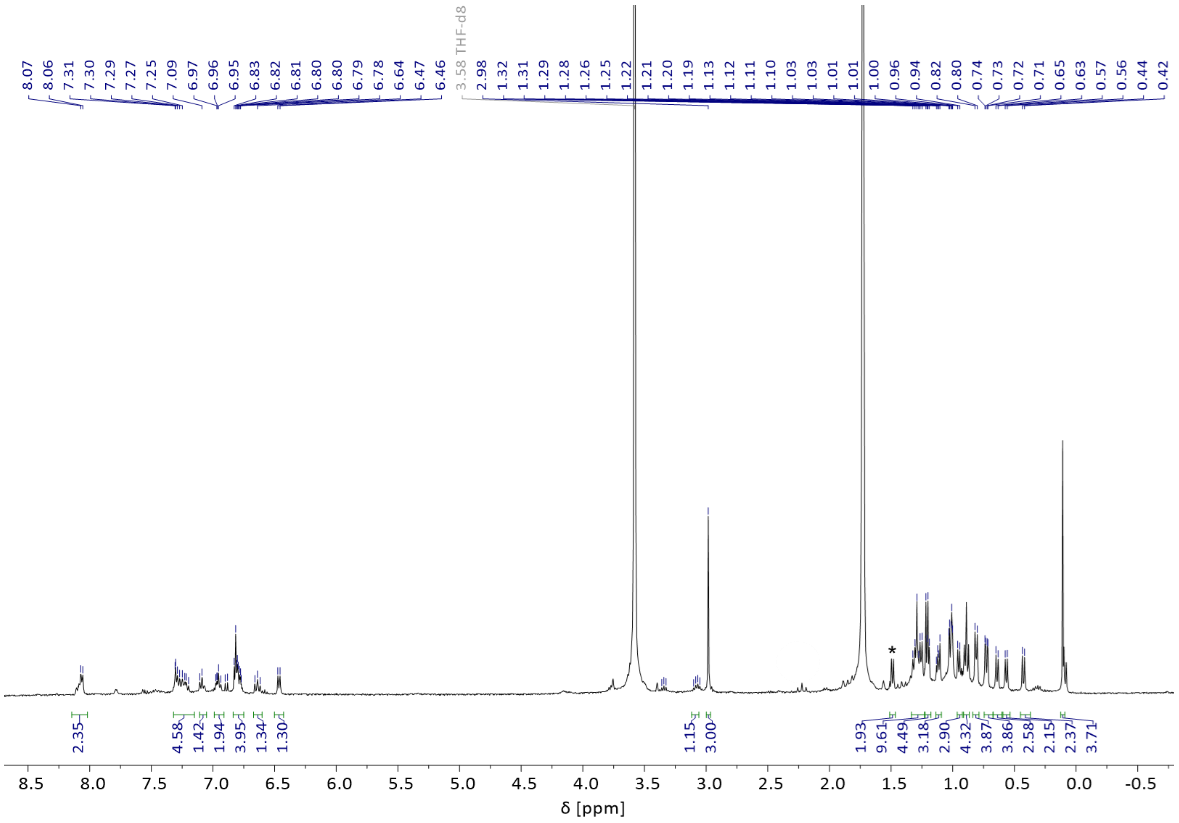

Figure S31. ^{1}H NMR spectrum of **8** as a solution in THF-d_8 at ambient temperature; * indicates small amounts of ^{Dipp}NHC.

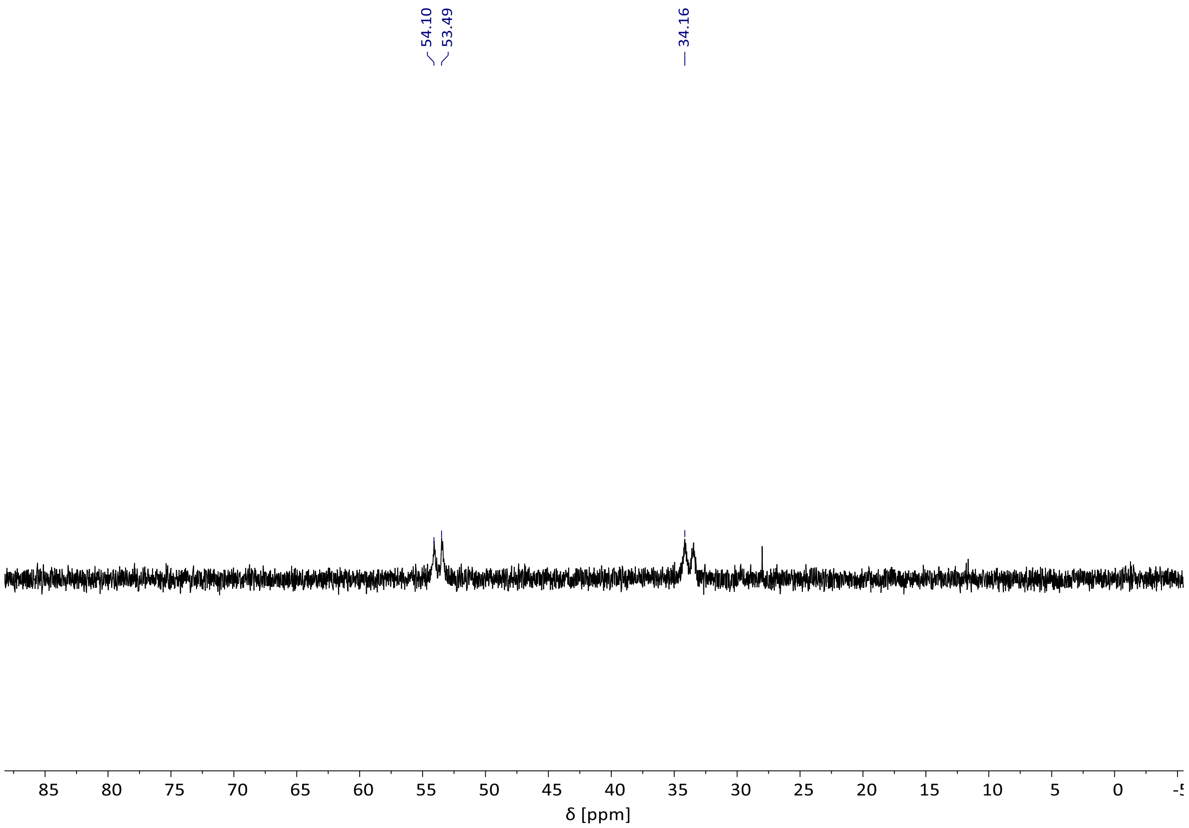

Figure S32. $^{31}P\{^1H\}$ NMR spectrum of **8** as a solution in THF-d_8 at ambient temperature.

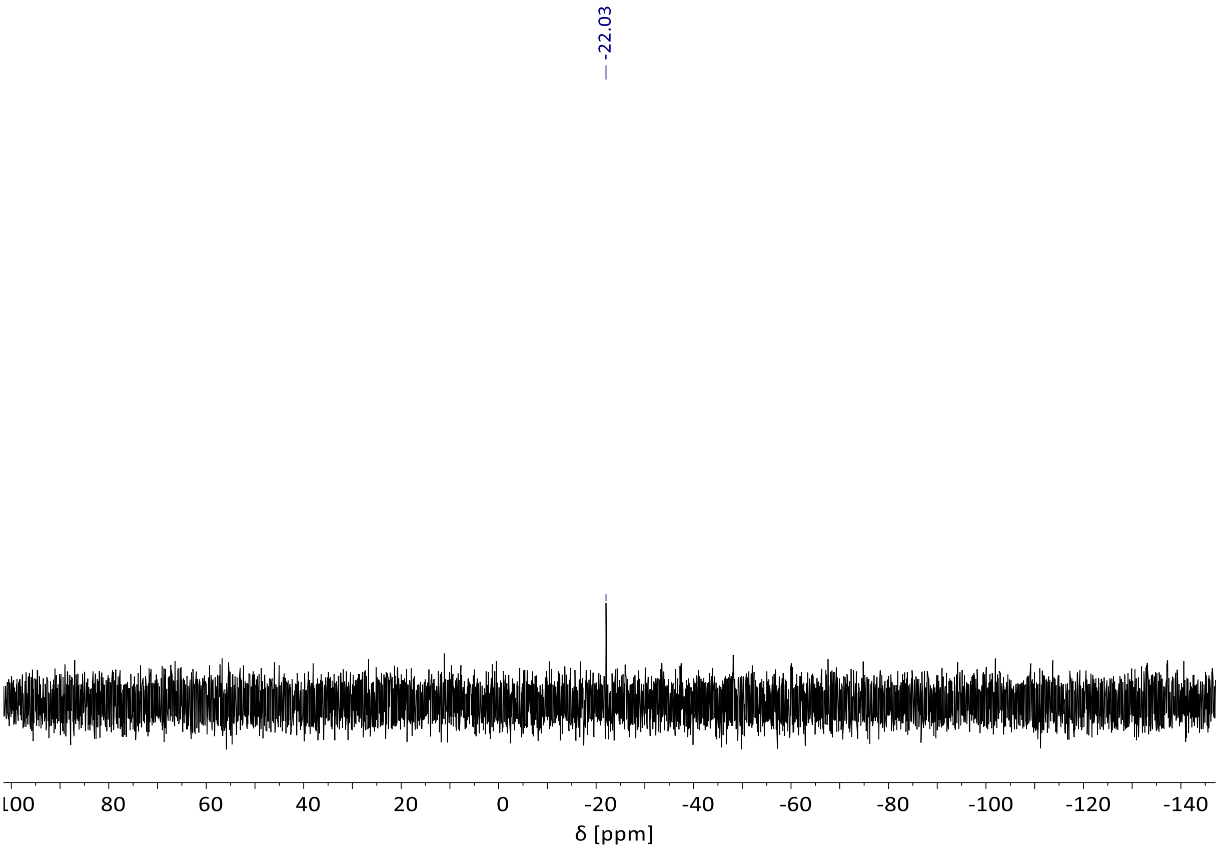

Figure S33. $^{29}Si\{^1H\}$ NMR spectrum of **8** as a solution in THF-d_8 at ambient temperature

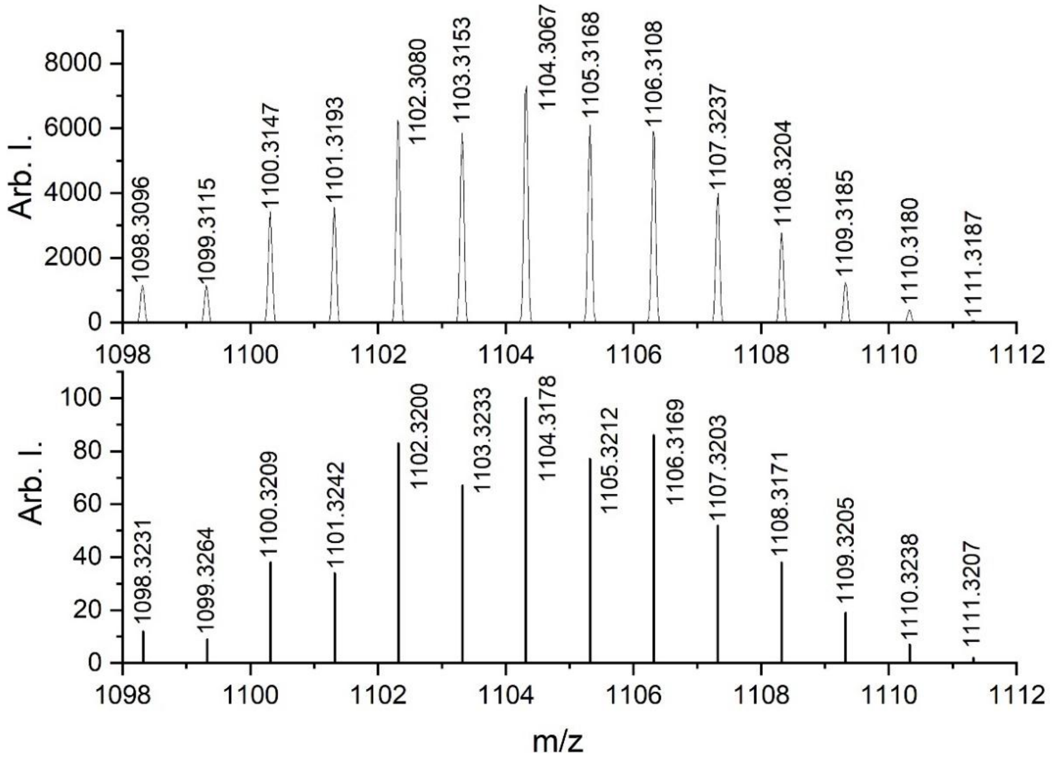

Figure S34. *Top:* Cutout from LIFDI/MS of **8;** *Bottom:* Calculated MS spectrum of [**8**-DMAP-BAr_4^F]$^+$.

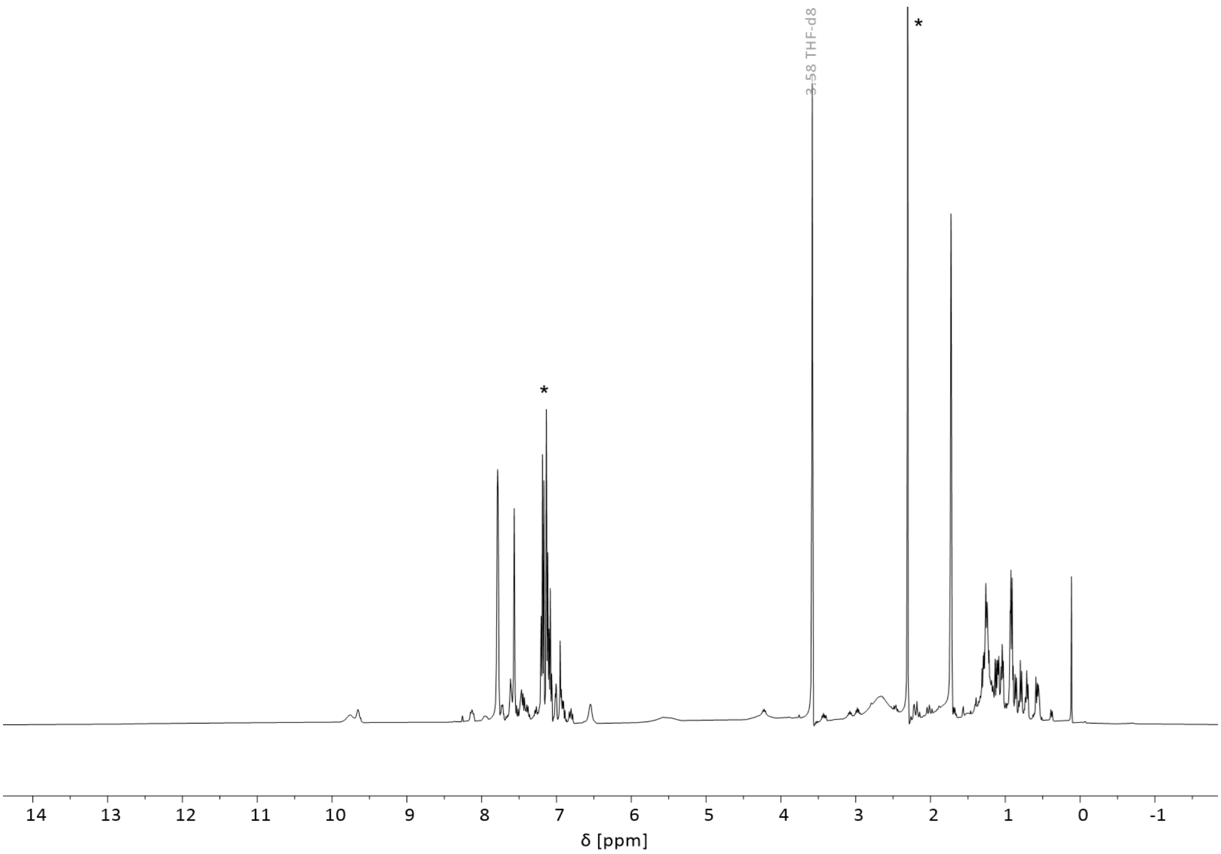

Figure S35. ^{1}H NMR spectrum of reaction of **7** and **3** as a solution in THF-d_8 at ambient temperature; * indicates toluene.

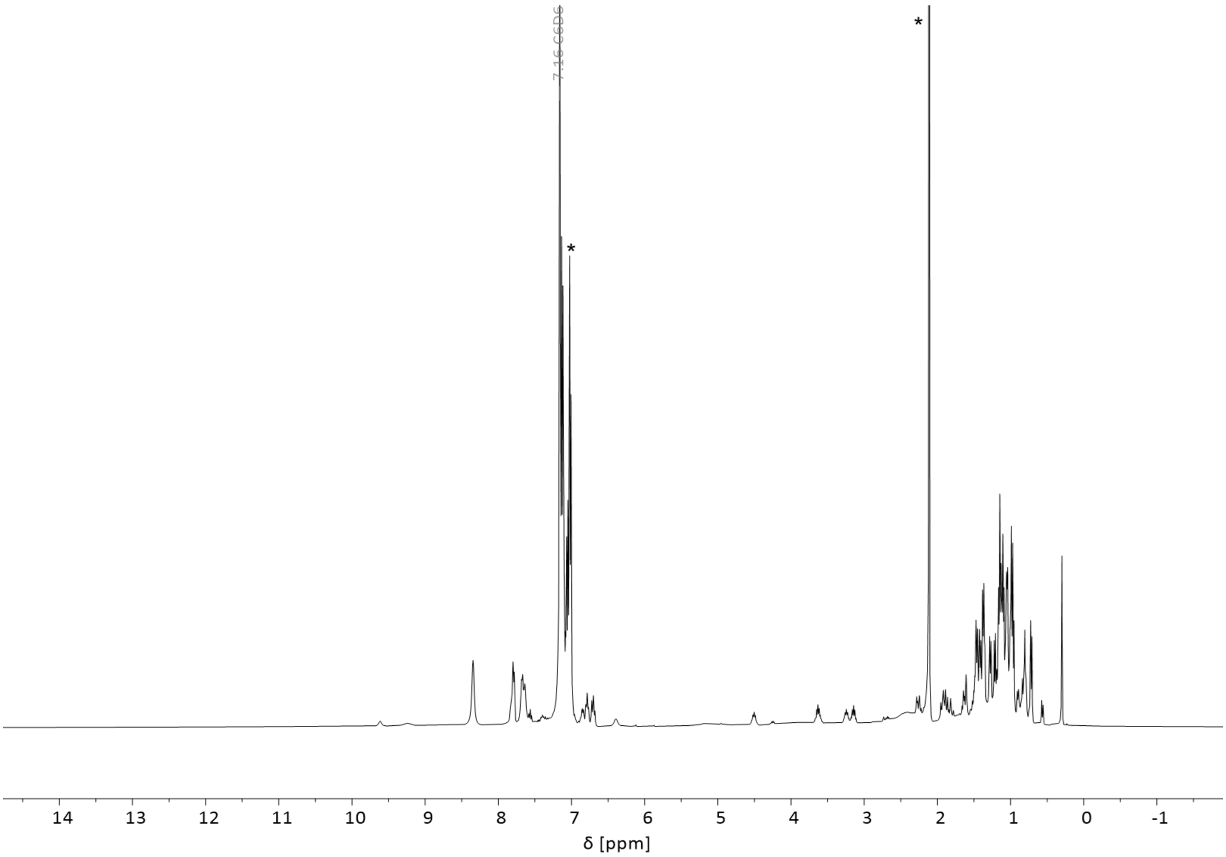

Figure S36. ^{1}H NMR spectrum of the reaction of **7** and **3** as a solution in C_6D_6 at ambient temperature; * indicates toluene.

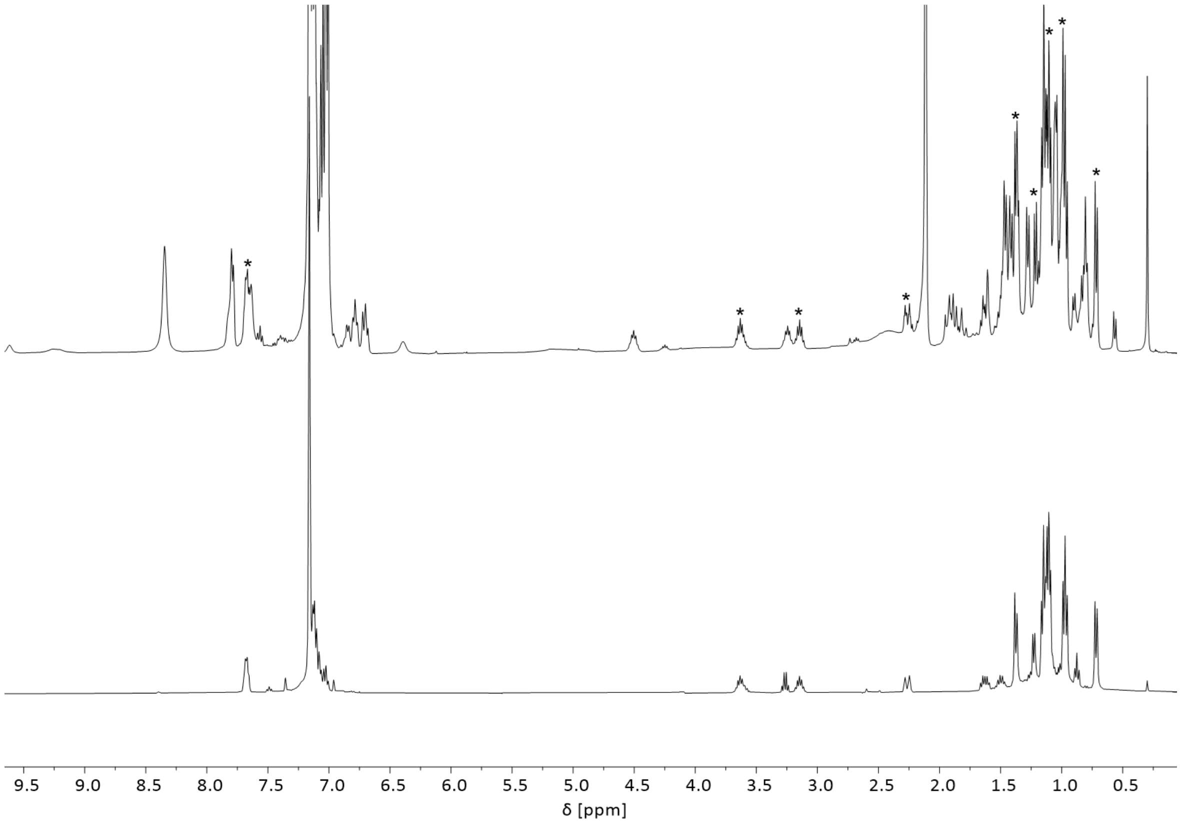

Figure S37. Comparison of the ^{1}H NMR spectrum of (^{PhiP}DippGePPh)$_2$ (**5**) (*Bottom*) to the reaction of **7** and **3** in C_6D_6 (*Top*); * marks **5** in the reaction mixture.

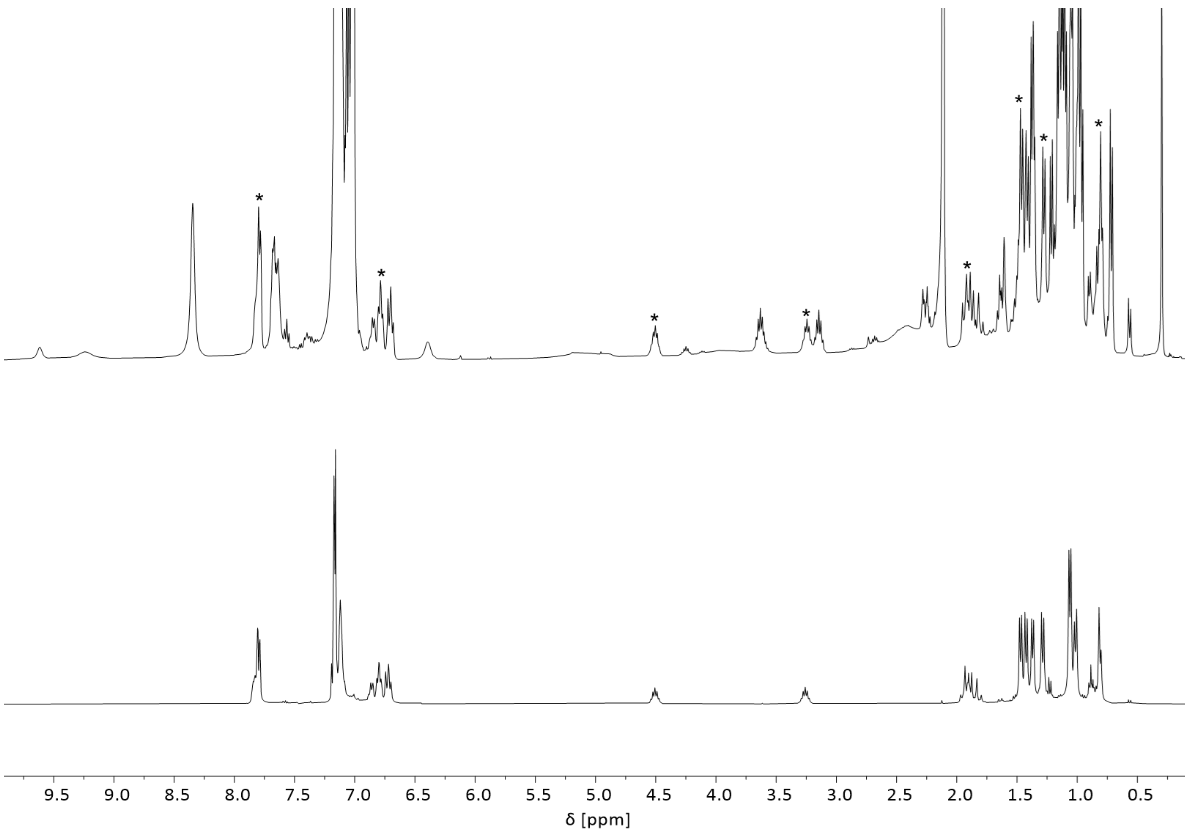

Figure S38. Comparison of the ^{1}H NMR spectrum of ^{PhiP}DippGePh (*Bottom*) to the reaction of **7** and **3** in C_6D_6 (*Top*); * marks ^{PhiP}DippGePh in the reaction mixture.

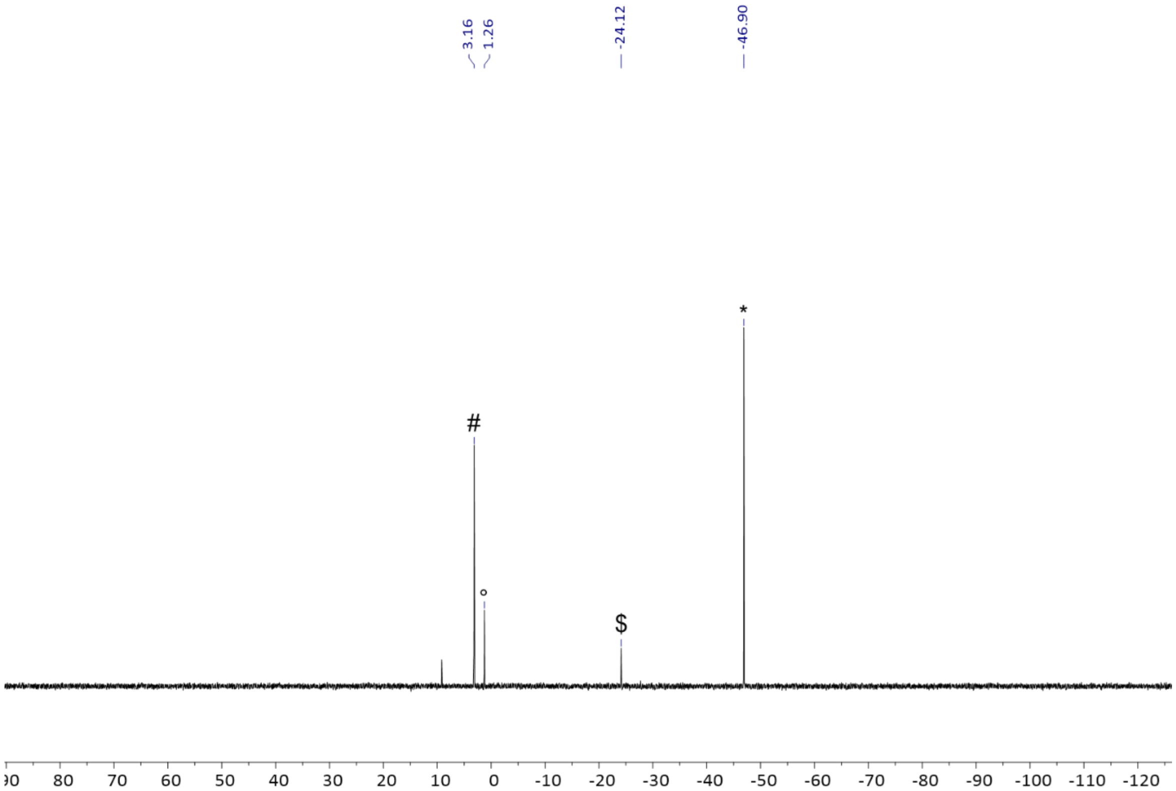

Figure S39. ^{31}P{^{1}H} NMR spectrum of the reaction of **7** and **3** as a solution in C_6D_6 at ambient temperature; * indicates **5**, # marks ^{PhiP}DippGePh, ° marks ^{PhiP}DippGeCl and $ indicates small amount of ^{PhiP}DippNH.

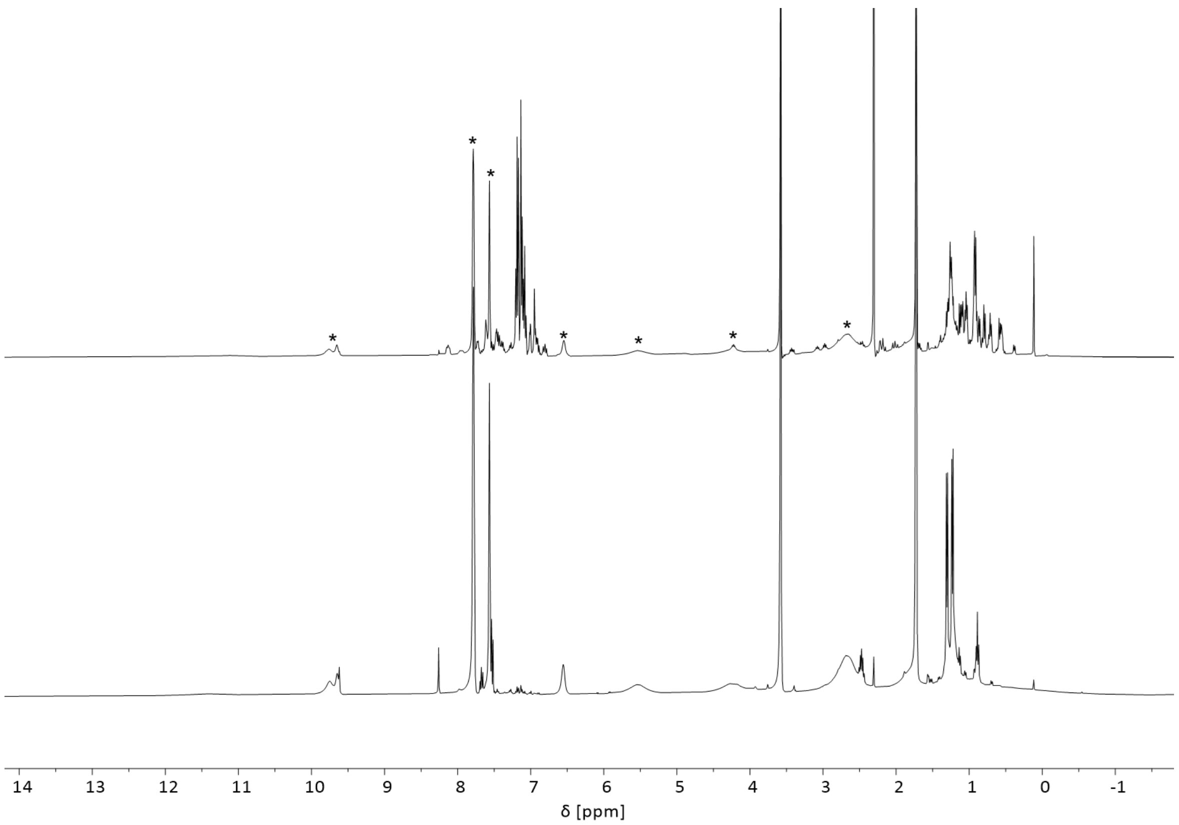

Figure S40. Comparison of the ^{1}H NMR spectrum of [^{PhiP}DippGe·Co·IPr][BAr$^F{}_4$], **2** (*Bottom*) to the reaction of **7** and **3** in THF-*d*$_8$ (*Top*); * marks **2** in the reaction mixture.

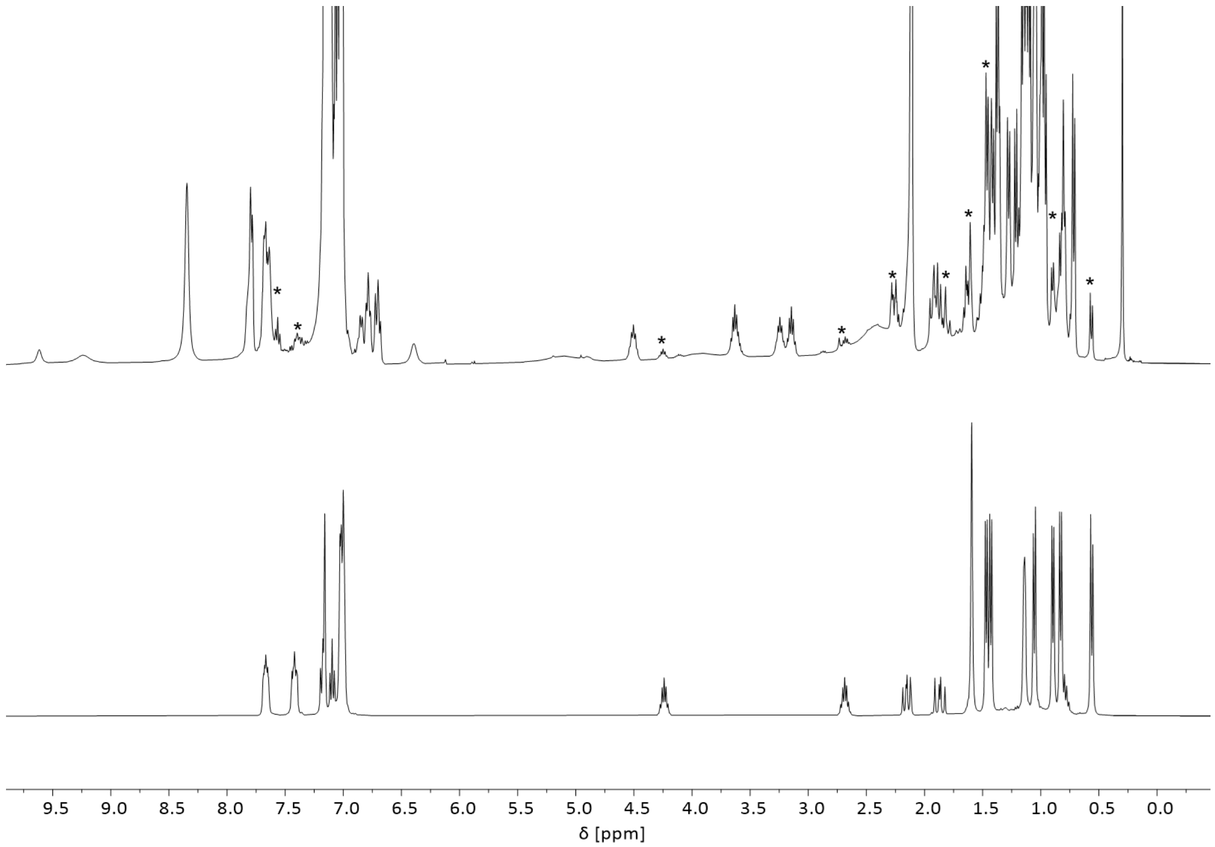

Figure S41. Comparison of the ^{1}H NMR spectrum of ^{PhiP}DippGeCl (*Bottom*) to the reaction of **7** and **3** in C_6D_6 (*Top*); * marks ^{PhiP}DippGeCl in the reaction mixture.

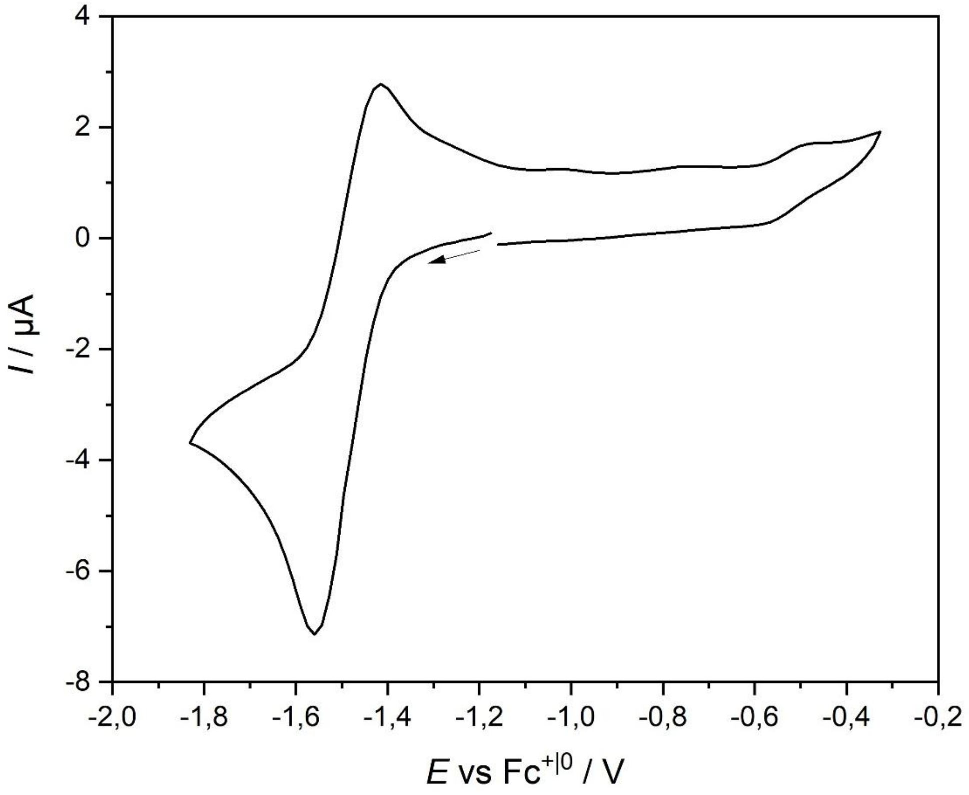

Figure S42. Cyclic voltammogram of a 1 mM solution of **2** in THF under Argon; 0.1 M $[N(n\text{-}Bu)_4][PF_6]$.; 100mV/s scan rate.

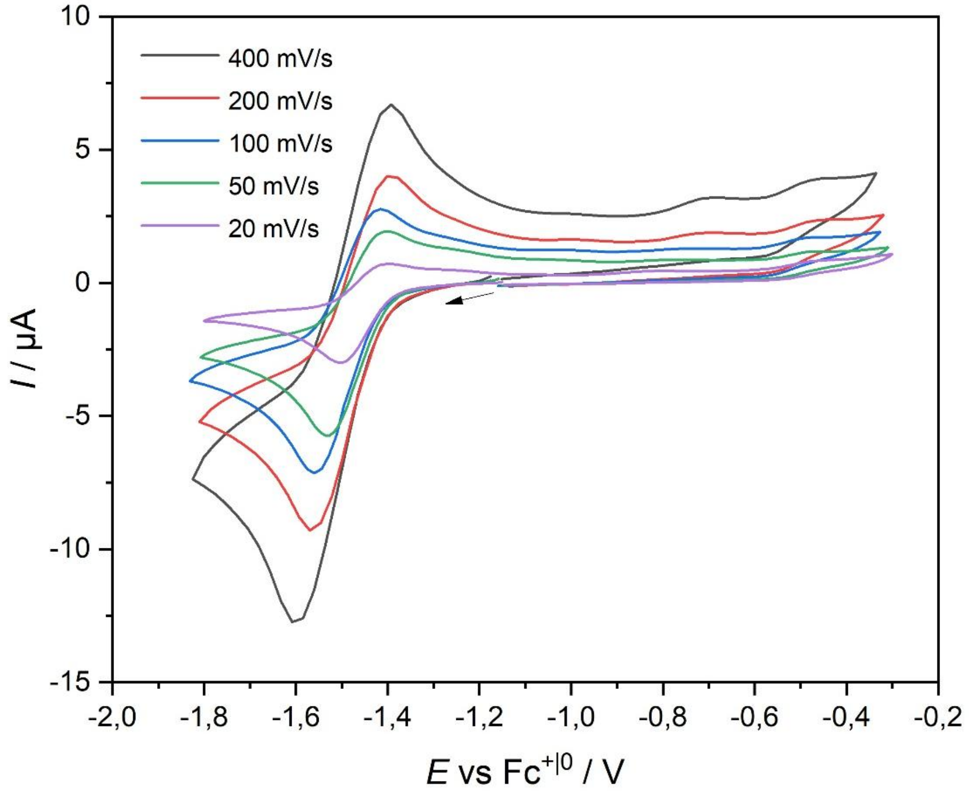

Figure S43. Cyclic voltammogram of a 1 mM solution of **2** in THF under Argon; 0.1 M $[N(n\text{-}Bu)_4][PF_6]$.

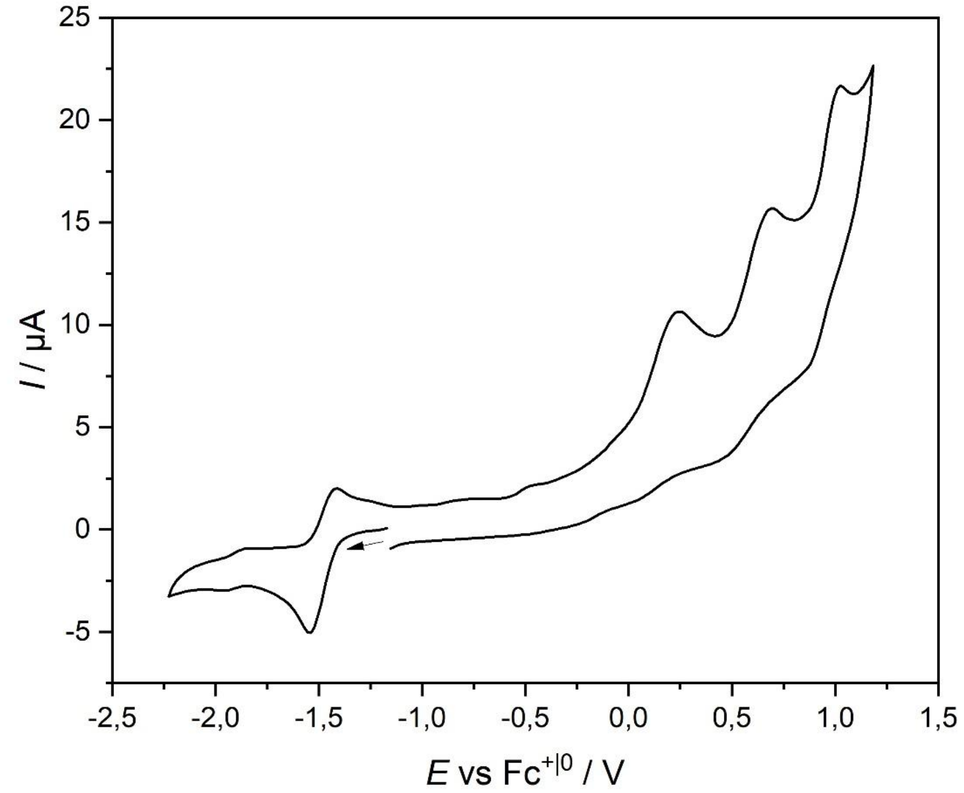

Figure S44. Cyclic voltammogram of a 1 mM solution of **2** in THF under Argon; 0.1 M [N(n-Bu)$_4$][PF$_6$].; 100mV/s scan rate.

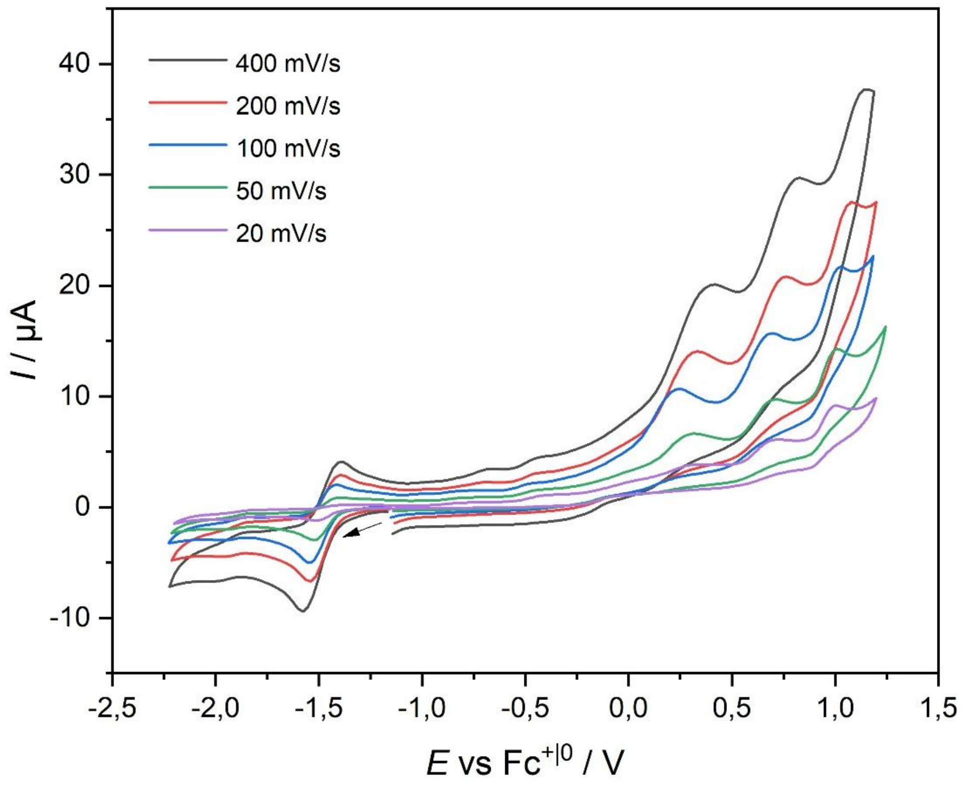

Figure S45. Cyclic voltammogram of a 1 mM solution of **2** in THF under Argon; 0.1 M [N(n-Bu)$_4$][PF$_6$].

2. X-ray crystallographic details

Single crystals of **2**, **4**, **5**, **7**, and **8**, suitable for X-ray structural analysis were mounted in perfluoroalkyl ether oil on a nylon loop and positioned in a 150 K cold N_2 gas stream. Data collection was performed with a STOE StadiVari diffractometer (MoKα radiation) equipped with a DECTRIS PILATUS 300K detector. Structures were solved by using SHELXT-16,[9] and refined by full-matrix least-squares calculations against F^2 (SHELXL-2018).[10] The positions of the hydrogen atoms were calculated and refined using a riding model. All non-hydrogen atoms were treated with anisotropic displacement parameters. Crystal data, details of data collections, and refinements for all structures can be found in their CIF files, which are available free of charge *via* www.ccdc.cam.ac.uk/data_request/cif, and are summarized in Table S3. SQUEEZE was used to mask disordered solvent molecules in the molecular structures of **4** and **8**. Additionally, the data is refining **4** was restricted to 2θ of 50°, given the low ratio of collected to unique reflections for this species.

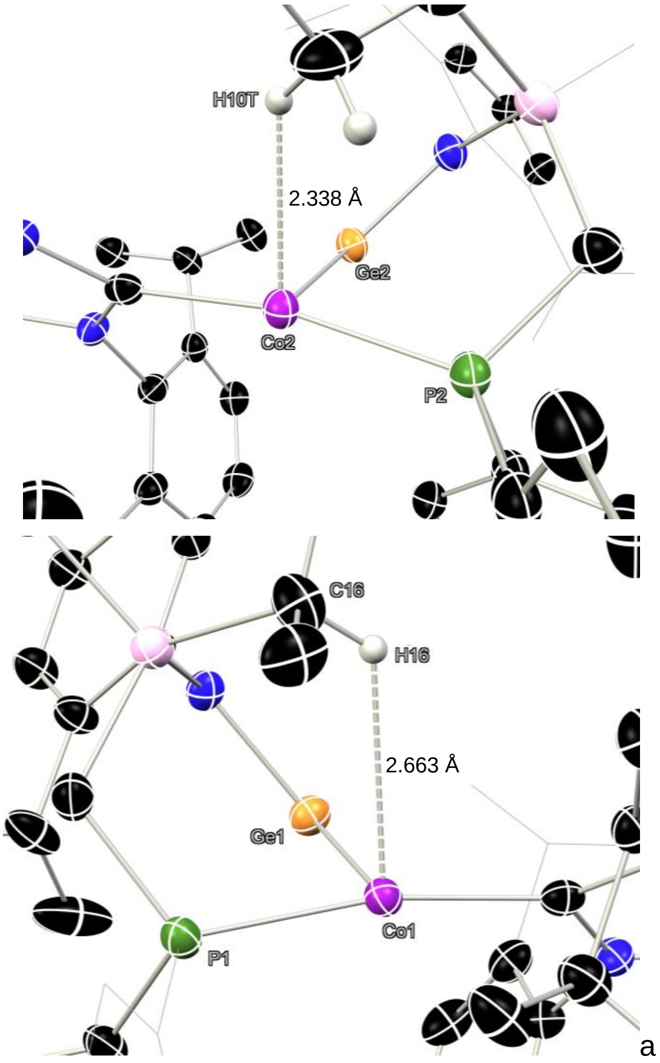

Figure S46. The agostic interactions at play in **2.**

Response to CIF check alerts:

Complex 2

Alert level B

```
_vrf_PLAT220_2
;
PROBLEM: NonSolvent   Resd 1  C   Ueq(max)/Ueq(min) Range        6.4 Ratio
RESPONSE: This is due to disorder in iPr groups. Which we attempted to
model the most extreme cases, the ellipsoids remain large. This, however,
does not effect to overall publishable quality of the data.
;
_vrf_PLAT221_2
;
PROBLEM: Solv./Anion  Resd 3  F   Ueq(max)/Ueq(min) Range        9.1 Ratio
RESPONSE: This is due to significant disorder in the BArF anion, which has
been modelled as such, and had multiple constraints applied.
;
_vrf_PLAT230_2
;
PROBLEM: Hirshfeld Test Diff for    F38      --C171     .        7.7 s.u.
RESPONSE: This is due to significant disorder in the BArF anion, which has
been modelled as such, and had multiple constraints applied.
;
_vrf_PLAT230_2_2
;
PROBLEM: Hirshfeld Test Diff for    F39      --C171     .       10.9 s.u.
RESPONSE: This is due to significant disorder in the BArF anion, which has
been modelled as such, and had multiple constraints applied.
;
_vrf_PLAT230_2_3
;
PROBLEM: Hirshfeld Test Diff for    F41      --C172     .       23.0 s.u.
RESPONSE: This is due to significant disorder in the BArF anion, which has
been modelled as such, and had multiple constraints applied.
;
_vrf_PLAT230_2_4
;
PROBLEM: Hirshfeld Test Diff for    F47      --C180     .        8.0 s.u.
RESPONSE: This is due to significant disorder in the BArF anion, which has
been modelled as such, and had multiple constraints applied.
;
_vrf_PLAT910_2
;
PROBLEM: Missing FCF Reflection(s) Below Theta(Min)[Deg]=       2.17 Note
RESPONSE: Given the overall quality and completeness of the data (R1 =
5.51%; completeness = 99.7%), this does not affect the accuracy of the
structure.
```

Complex 4

Alert level B

```
_vrf_DENSD01_4
;
PROBLEM: The ratio of the submitted crystal density and that
RESPONSE: This is due to the inclusion of solvent molecules in the .ins
file following the SQUEEZE function. This corrects the MW and D values
correct.
;
```

```
_vrf_PLAT026_4
;
PROBLEM: Ratio Observed / Unique Reflections (too) Low ..        37% Check
RESPONSE: Though this may be the case due to the small size and sensitivity
of this crystal, it does not affect the completeness or quality of the data
(99.5% and 8.59%, respectively), which are both of a publishable standard.
Better quality data could not be collected for this compound as yet.
;
_vrf_PLAT046_4
;
PROBLEM: Reported Z, MW and D(calc) are Inconsistent ....      1.263 Check
RESPONSE: This is due to the inclusion of solvent molecules in the .ins
file following the SQUEEZE function. This corrects the MW and D values
correct.
;
_vrf_PLAT910_4
;
PROBLEM: Missing FCF Reflection(s) Below Theta(Min)[Deg]=       2.34 Note
RESPONSE: Given the overall quality and completeness of the data (R1 =
8.59%; completeness = 99.5%), this does not affect the accuracy of the
structure.
```

Complex 8

Alert level B

```
_vrf_DENSD01_8
;
PROBLEM: The ratio of the submitted crystal density and that
RESPONSE: This is due to the inclusion of solvent molecules in the .ins
file following the SQUEEZE function. This corrects the MW and D values
correct.
;
_vrf_PLAT046_8
;
PROBLEM: Reported Z, MW and D(calc) are Inconsistent ....      1.154 Check
RESPONSE: This is due to the inclusion of solvent molecules in the .ins
file following the SQUEEZE function. This corrects the MW and D values
correct.
;
_vrf_PLAT910_8
;
PROBLEM: Missing FCF Reflection(s) Below Theta(Min)[Deg]=       2.46 Note
RESPONSE: Given the overall quality and completeness of the data (R1 =
4.73%; completeness = 99.7%), this does not affect the accuracy of the
structure.
```

Table S2. SQUEEZE details for **4**

Compound	*Void Nr*	*Void average…*			*Void Volume*	*Void Count Electrons*	*Void Content*
		x	*y*	*z*			
**4**	1	0.006	0.5	0.5	691	146	4(C_5H_{12})
	2	0.202	0.742	0.803	22	4	*NA*
	3	0.488	-0.014	0.254	45	8	*NA*
	4	0.512	0.014	0.746	45	8	*NA*
**8**	1	0	0	0	464	57	2(C_5H_{12}), 2($C_2H_{10}O$)

Table S3. Summary of X-ray crystallographic data for compounds **2**-**8**.

	**2**	**4**	**5**	**7**	**8**
empirical form.	$C_{90}H_{91}BCoF_{24}GeN_3PSi$	$C_{96}H_{96}BCoF_{24}GeN_3PSi$	$C_{50}H_{76}Ge_2N_2P_2Si_2$	$C_{62}H_{86}Ge_2N_2P_2Si_2$	$C_{63}H_{91}CoGe_2N_4P_2Si_2$
formula wt	1872.04	1949.14	968.42	1122.62	1226.62
crystal syst.	orthorhombic	triclinic	monoclinic	monoclinic	triclinic
space group	*Pca2₁* → $Pca2_1$	P-1	$P2_1/n$	$P2_1/n$	P-1
a (Å)	32.500(7)	13.450(3)	15.500(3)	13.860(3)	13.120(3)
b (Å)	19.920(4)	16.990(3)	9.040(18)	10.420(2)	13.450(3)
c (Å)	28.050(6)	22.840(5)	18.050(4)	20.490(4)	21.660(4)
α (deg.)	90	94.15(3)	90	90	88.00(3)
β (δεγ)	90	99.57(3)	99.30(3)	93.40(3)	76.10(3)
γ (deg.)	90	92.39(3)	90	90	72.30(3)
vol (Å^3)	18160(6)	5125.4(19)	2495.9(9)	2954.0(10)	3531.6(14)
Z	8	2	2	2	2
ρ(calc) (g.cm^{-3})	1.369	1.356	1.289	1.262	1.291
μ (mm^{-1})	0.639	0.574	1.351	1.151	1.202
F(000)	7688	2172	1024	1188	1460
T (K)	150(2)	150(2)	150(2)	150(2)	150(2)
reflns collect.	151789	64594	30334	14111	49478
unique reflns	35383	17935	4893	5768	13892
R_{int}	0.0487	0.1604	0.0259	0.0693	0.0592
R1 [$I > 2\sigma(I)$]	0.0551	0.0859	0.0295	0.0576	0.0409
*w*R2 (all data)	0.1492	0.2429	0.0696	0.1547	0.0943
CCDC No.	2382130	2382132	2382129	2434139	2382131

Full molecular structures of all new compounds (*i.e.* 2, 4, 5, 7, and 8) in order, with ellipsoids at 30% probability and hydrogen atoms omitted:

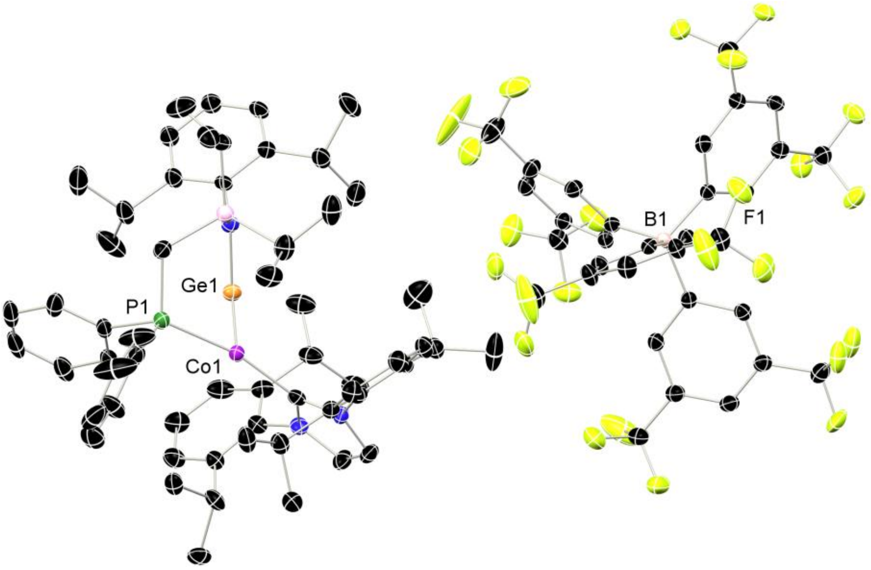

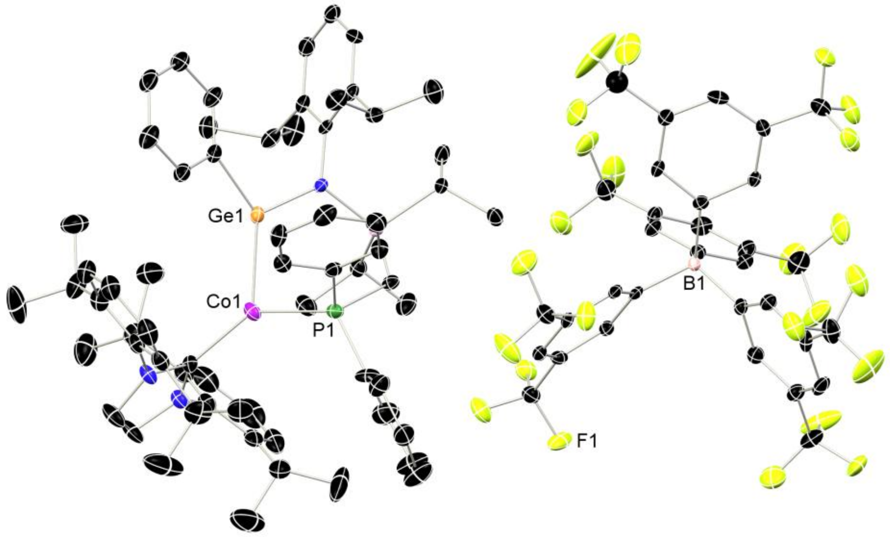
Ge1
Co1
P1
B1
F1

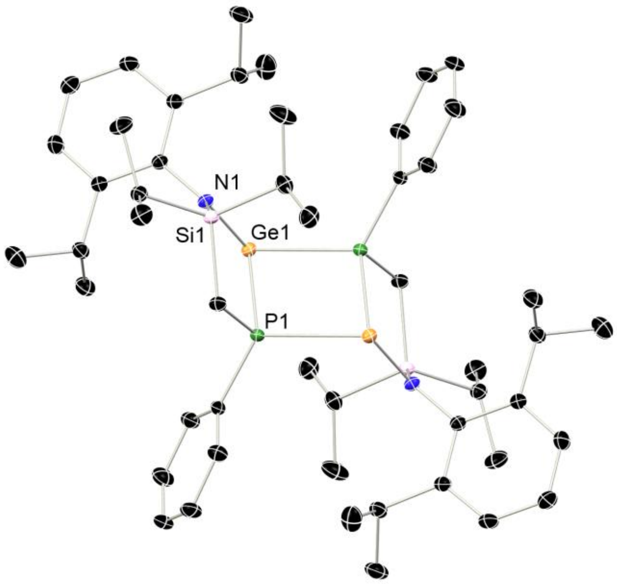
N1
Si1
Ge1
P1

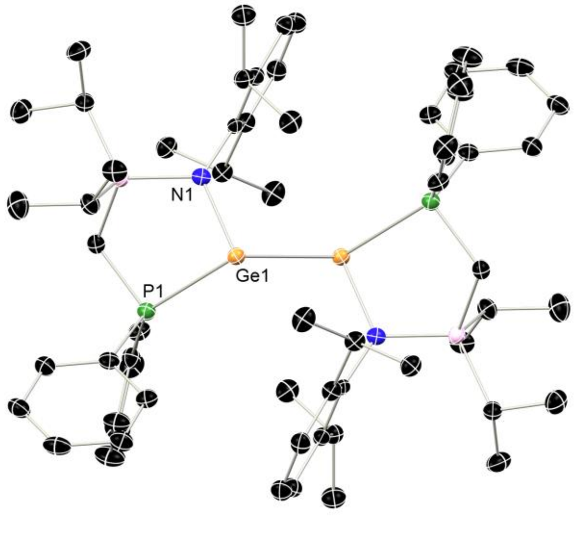
N1
P1
Ge1

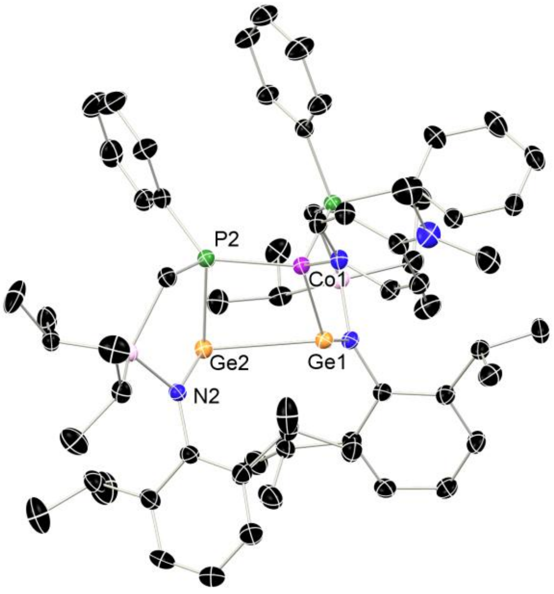
P2
Co1
Ge2
Ge1
N2

3. Computational Methods and Details

Geometry optimizations and harmonic frequency calculations were performed using the ORCA program package[11] (Version 5.0.4) employing the PBE0[12] density functional combined with the def2-SVP[13] basis set and the D3 dispersion correction with Becke-Johnson dampening.[14] Optimized structures were characterized as minima by eigenvalue analysis of the computed Hessians. Improved energies on optimized structures were obtained in single point calculations with the larger def2-TZVP basis set.[13] Quasi restricted orbitals (QROs)[15] and unrestricted corresponding orbitals (UCOs) were generated using the UNO/UCO keyword in ORCA 5.0.4. The fractional occupation number weighted electron density (FOD) analysis[16] was performed with the PBE0 functional and a smearing temperature of 10000 K in ORCA 5.0.4. NBO analyses were performed using NBO6[17] interfaced with the Gaussian16 program.[18]

Partly contracted NEVPT2 calculations[19] based on CASSCF wave functions employing the def2-TZVP basis set were used to generate natural orbitals for QTAIM and ELF analysis. WFN files for QTAIM were generated from the natural orbital file using the orca2mkl tool for conversion into the molden format. The molden files were then converted into the WFN format using MOLDEN2AIM.[20] Direct conversion using the orca2aim tool produced faulty WFN-files, which led to missing electrons in the QTAIM analysis.

For a smaller model system, single point calculations using the explicitly correlated MRCI-F12[21] method based on CASSCF(5,5) wave functions were performed using the Molpro2020.2 program.[22] The F12-optimized correlation-consistent polarized triple-zeta orbital[23] and auxiliary[24] basis sets of the cc-pVTZ-F12 family were used in these calculations for all non-metal atoms. For the Co atom, the aug-cc-pVTZ orbital basis[25] was used in conjunction with the universal JKfit option for integral fitting in the Fock matrix construction along with the aug-cc-pVTZ/MP2Fit[26] auxiliary basis used for the many-electron integrals and CABS representation. For the Ge atom the aug-cc-pVQZ orbital basis was used in conjuction with the cc-pV5Z/JK[27] basis for integral fitting along with the aug-cc-pvQZ/MP2fit[24(b)] auxiliary basis used for the many-electron integrals and CABS representation. The basis sets were chosen based on the presence of functions with high angular momentum necessary for explicitly correlated post Hartree-Fock calculations. Within the MRCI-F12 calculation 40 orbitals consisting of core orbitals of non-hydrogen atoms were excluded from the MRCI treatment.

QTAIM analyses were performed with the AIMALL program.[28] The Multiwfn program[29] was used to extract 1D-Laplacian profiles along bond paths and to perform ELF analyses.[30] Energy decomposition analysis calculations were performed using the ADF program (version 2019.304)[31] employing the PBE0 functional combined with the D3 dispersion correction and the TZ2P basis set.[32]

Pictures of molecular structures were generated with the Cylview[33] and ChemCraft[34] programs. Averaging of α/β spin orbitals was performed in ChemCraft.

Structural Comparison of Compound 2

Table S4. Selected metrics of the PBE0-D3BJ/def2-SVP optimized and the X-RAY structure for the full molecular model of **2**. Hydrogen atoms and isopropyl groups of Dip-substituents not shown.

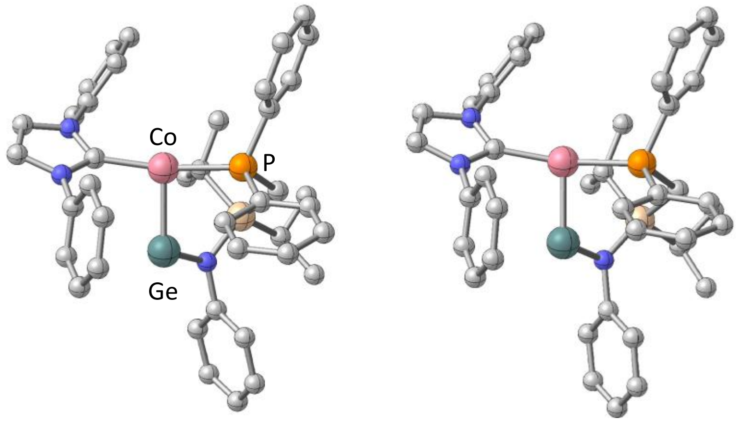

	PBE0-D3BJ	exp. X-RAY
Co–Ge	2.37 Å	2.29 Å
Co–P	2.27 Å	2.24 Å
Co–C^{NHC}	1.96 Å	1.97 Å
C^{NHC}–Co–P	168.3 °	167.5 °
C^{NHC}–Co–Ge	98.2 °	100.6 °

Additional DFT results on the electronic structure of 2

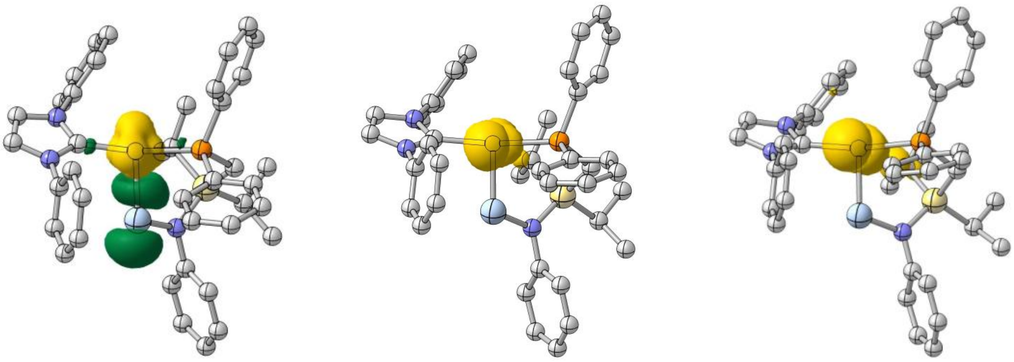

Co (⟨S^2⟩ = 1.26)		Rh (⟨S^2⟩ = 0.75)		Ir (⟨S^2⟩ = 0.75)	
	Spin pop.		Spin pop.		Spin pop.
Co	1.58	Rh	0.79	Ir	0.83
Ge	–0.54	Ge	0.11	Ge	0.01
P	–0.01	P	0.02	P	0.01

Figure S47. Spin density plots of the full molecular model of **2** and the corresponding rhodium and iridium complexes (the latter two are experimentally unknown) with natural spin populations, hydrogen atoms and isopropyl groups of Dip-substituents not shown (UPBE0/def2-TZVP results, isosurfaces at ±0.005 a_0^{-3}).

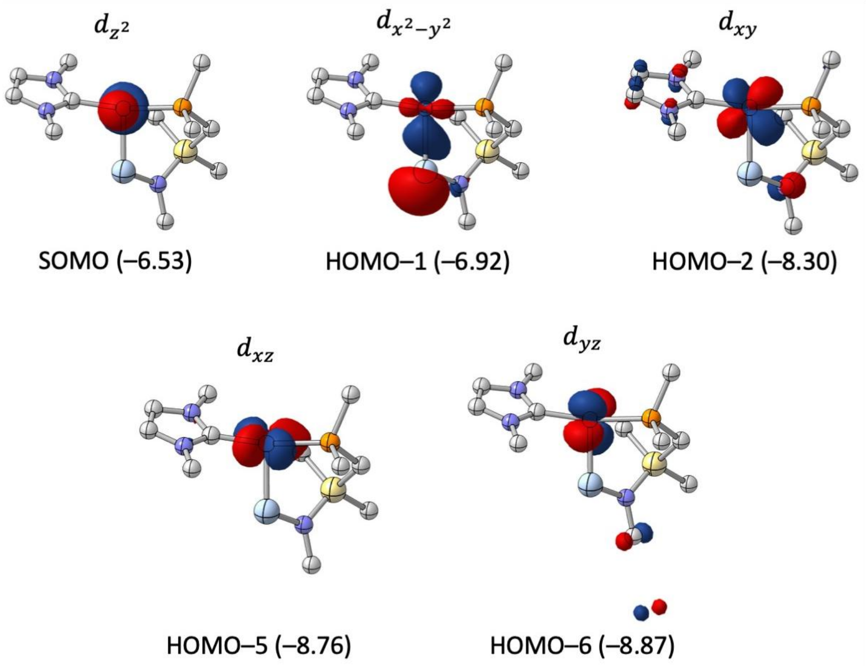

Figure S48. Selected QROs of the full molecular model of **2** involving the cobalt d-orbitals, hydrogen atoms, aryl substituents and silicon isopropyl groups not shown (UPBE0/def2-TZVP results, orbital energies in eV, isosurfaces at ±0.05 $a_0^{-3/2}$).

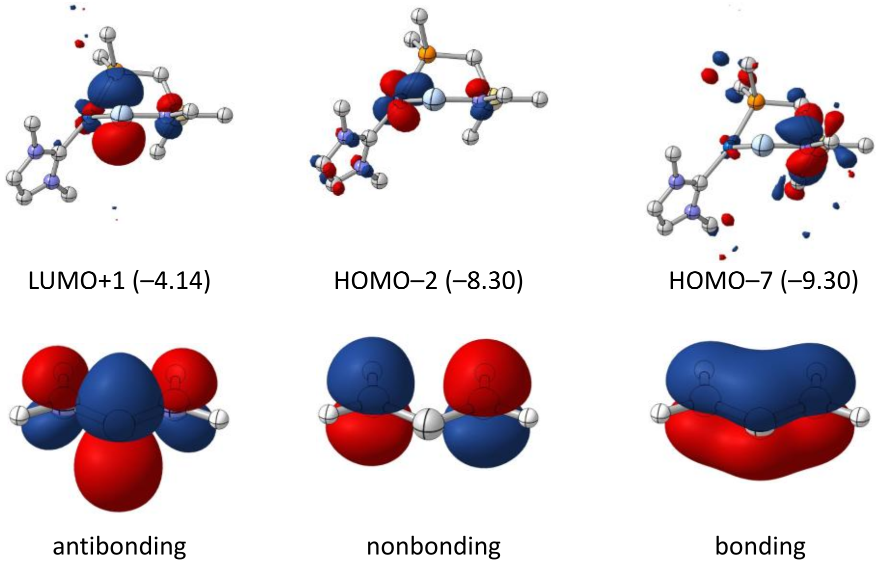

Figure S49. *Top*: Selected QROs highlighting the Co–Ge–N π-interaction in the full molecular model of **2**, hydrogen atoms, aryl substituents and silicon isopropyl groups not shown, orbital energies in eV; *bottom*: analogous π-orbitals in the parent diaminocarbene (UPBE0/def2-TZVP results, isosurfaces at ±0.05 $a_0^{-3/2}$).

Molecular models for further analysis

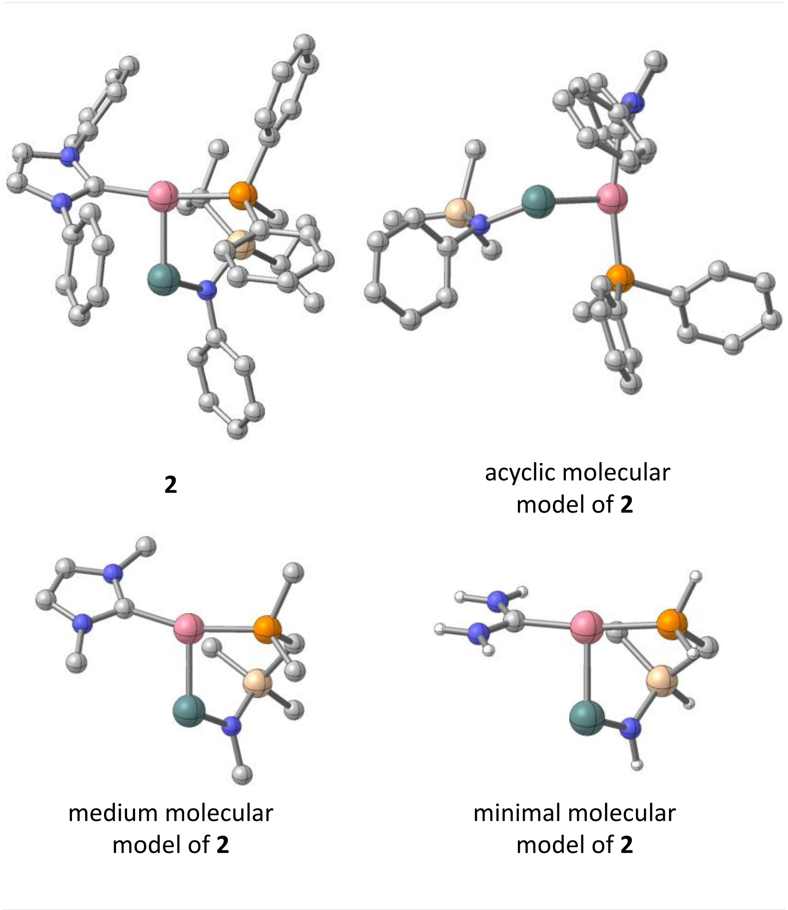

Figure S50. Optimized geometries of the full molecular model **2** and further models employed for bonding analysis (UPBE0/def2-SVP results).

Energy decomposition analysis of the acyclic molecular model of 2

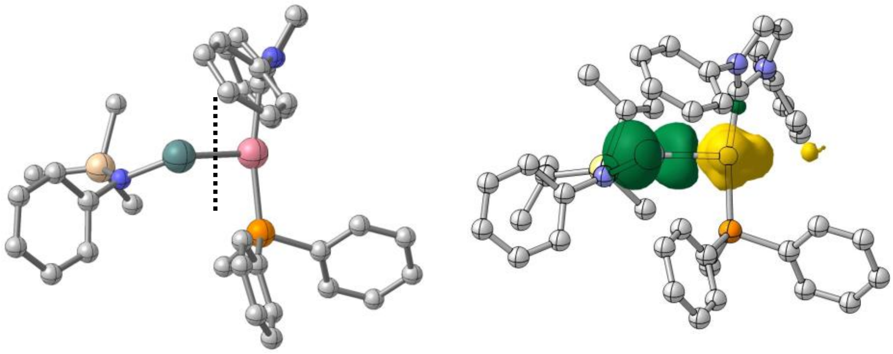

	$Ge^{+} + Co^{\bullet}$	$Ge^{\bullet} + Co^{+}$	$Ge^{-} + Co^{2+\bullet}$
Pauli Repulsion	159.8	134.2	186.9
Electrostatic	–109.4	–85.0	–249.4
Orbital	–134.8	–62.9	–181.4
Dispersion	–23.3	–23.8	–23.7
Total Bonding Interaction	–108.3	–37.5	–267.7

Figure S51. Spin density and energy decomposition analysis of the acyclic molecular model of **2**, isopropyl groups not shown (UPBE0/TZ2P results, energies in kcal mol^{-1}, isosurface at ±0.005 a_0^{-3}).

As the cyclic nature of the P/Ge-ligand prevents consistent fragmentation for EDA calculations we used an acyclic molecular model, in which the silicon-carbon bond is broken and a methyl group is placed on both ends (Fig. S47). During the geometry optimization the C^{NHC}–Co–P angle was frozen to retain the T-shaped structure (otherwise geometry optimization results in a trigonal-planar structure). According to the criterion of lowest orbital interaction for the choice of fragments[32] in EDA calculations, homolytic cleavage of the Ge–Co bond into $Ge^{\bullet}$ and Co^{+} fragments is favored over the two possible heterolytic fragmentations – as expected in view of the strong spin-polarization along this bond in the UKS wave function (cf. Fig. S43 and main text).

Choice of active space

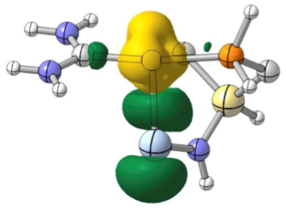

	Spin pop.
Co	1.62
Ge	–0.56
P	–0.01

	minimal model of **2**	**2**
Co–Ge	2.37 Å	2.37 Å
Co–P	2.26 Å	2.27 Å
Co–C^{NHC}	1.96 Å	1.96 Å
C^{NHC}–Co–P	172.9 °	168.3 °
C^{NHC}–Co–Ge	95.3 °	98.2 °

Figure S52. *Top*: UKS spin density and natural spin population of the minimal molecular model of **2** (UPBE0/def2-TZVP results, isosurface at ±0.005 a_0^{-3}) used for further MRCI-F12 benchmark calculations. *Bottom*: comparison of key structural parameters with the full molecular model of **2**.

The minimal molecular model for **2** preserves the T-shaped geometry and UKS spin polarization along the Co–Ge bond (Fig. S48). As a benchmark method for bonding analysis we performed explicitly correlated MRCI-F12 calculations on this molecular model based on CASSCF(5,5) wave functions. The minimum active space comprises the singly occupied d_{z^2}(Co) orbital, as well as the σ(Co–Ge)/σ*(Co–Ge) and the d_{xz}(Co)$_{\pi}$/π*(Co–Ge–N) correlating orbital pairs (Fig. S49). The larger (9,12) active space was chosen to include the 4d orbital space to capture potential double-d shell effects to electron correlation, which were found negligible, however (Fig. S50).[33] The same (5,5) active space captures all essential non-dynamic correlation effects also for the full molecular model of **2** (Fig. S51).

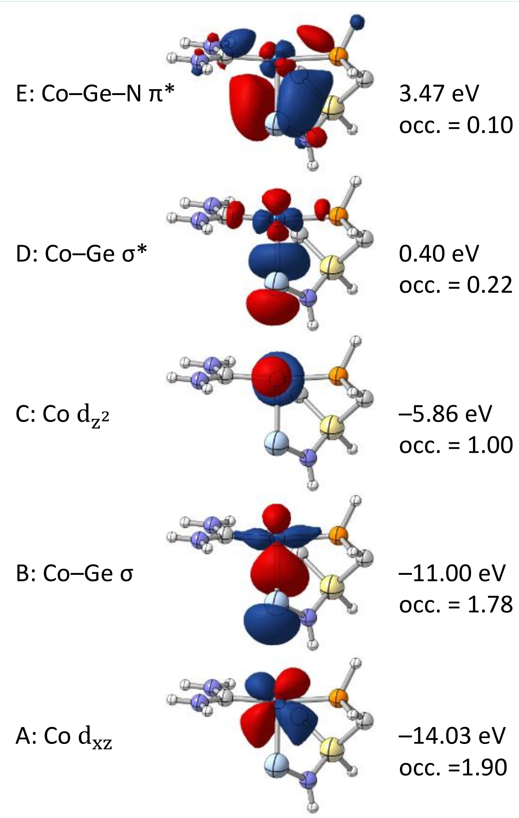

config. [ABCDE]	weight
[22100]	0.82
[20120]	0.06
[11111]	0.04
[21110]	0.04
[02102]	0.02

Figure S53. CASSCF(5,5) natural orbitals with characterization, energy and occupation numbers and leading configurations for the minimal molecular model of **2** (isosurface at ±0.05 $a_0^{-3/2}$).

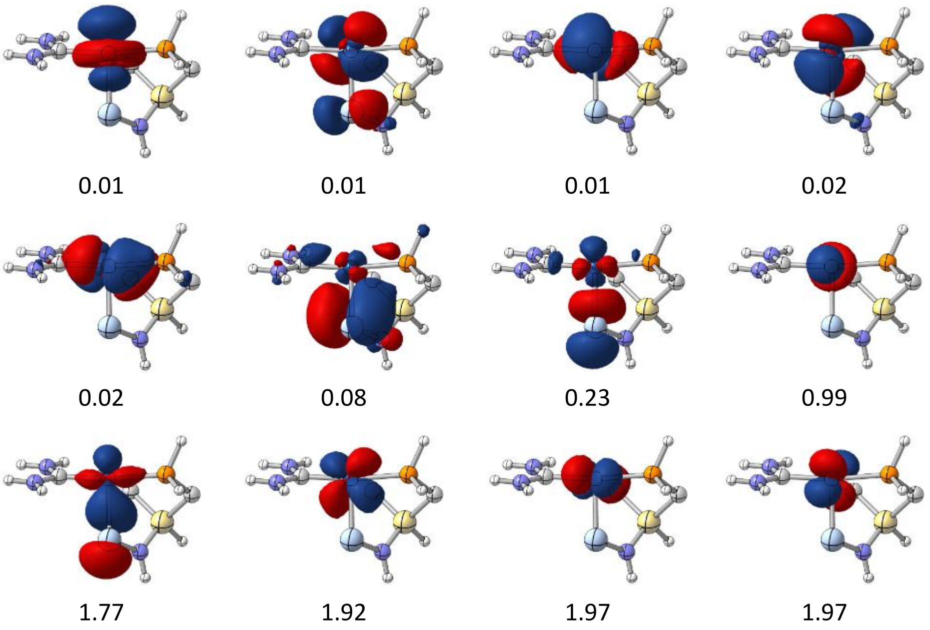

Figure S54. CASSCF(9,12) natural orbitals and occupation numbers of the minimal molecular model of **2**, (isosurface at ±0.05 $a_0^{-3/2}$).

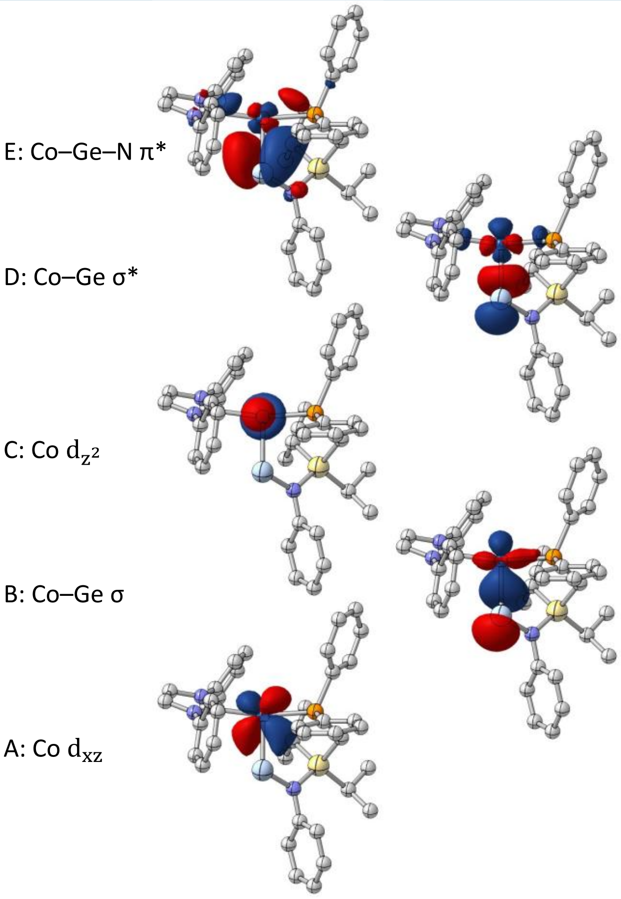

config. [ABCDE]	weight
[22100]	0.82
[20120]	0.06
[11111]	0.04
[21110]	0.04
[02102]	0.02

Figure S55. CASSCF(5,5) natural orbitals with characterization, energy and occupation numbers (NEVPT2 results in parenthesis) and leading configurations for the full molecular model of **2** (isosurface at ±0.05 $a_0^{-3/2}$).

Multireference and biradicaloid character of 2

Table S4. Comparison of metrics quantifying the multireference and biradicaloid character in the minimal molecular model of **2** obtained from CASSCF(5,5), NEVPT2, MRCI-F12 and UPBE0 (⟨S^2⟩ = 1.25) wave functions. $\langle S^2 \rangle_{excess}$ represents spin contamination of the UPBE0 wave function in excess of the value of 0.75 expected for the single unpaired d-electron present at Co.

MR metric	Method	Value
M	CASSCF	0.223
	NEVPT2	0.238
	MRCI-F12	0.222
n_{rad}	UPBE0 ($\langle S^2 \rangle_{excess} = 0.50$)	29.3%
	CASSCF	35.1%
	MRCI-F12	30.8%

Table S5. Comparison of the multireference and biradicaloid character in the full molecular model of **2** obtained from CASSCF(5,5), NEVPT2 and UPBE0 (⟨S^2⟩ = 1.26) wave functions. $\langle S^2 \rangle_{excess}$ represents spin contamination of the UPBE0 wave function in excess of the value of 0.75 expected for the single unpaired d-electron present at Co.

MR metric	Method	Value
M	CASSCF	0.231
	NEVPT2	0.241
n_{rad}	UPBE0 ($\langle S^2 \rangle_{excess} = 0.51$)	30.0%
	CASSCF	35.5%

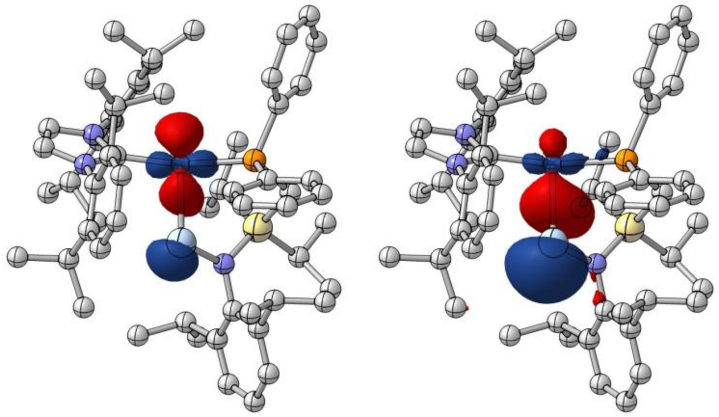

UCO 266α UCO 266β
⟨α|β⟩ = 0.72

Figure S56. Unrestricted corresponding orbitals (UCO) representing the Co–Ge σ-bond with imperfect ⟨α|β⟩ overlap in the full molecular model of **2** (UPBE0 results, isosurface at ±0.05 $a_0^{-3/2}$).

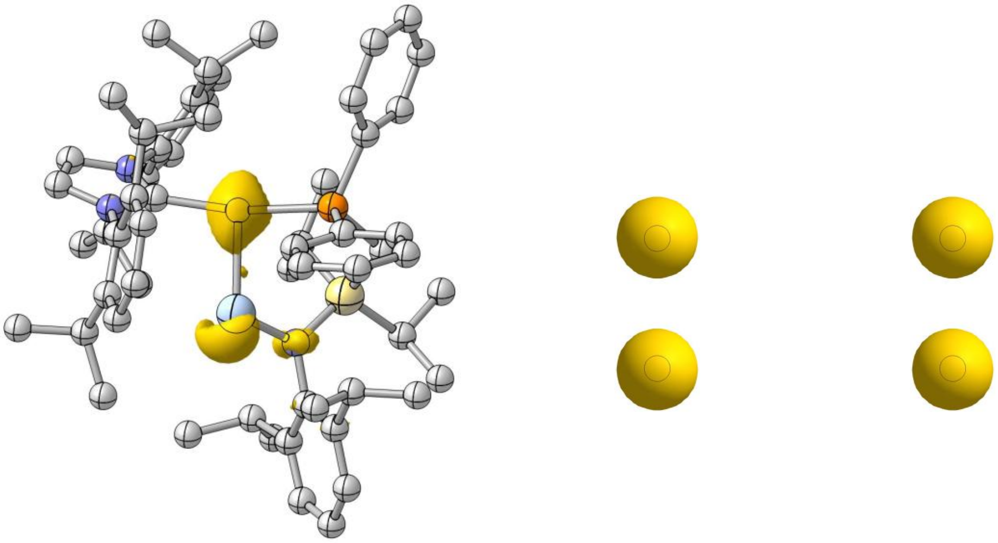

UKS, N_{FOD} = 2.241132 (N^{α}_{FOD} = 1.022821) (N^{β}_{FOD} = 1.218311)

RKS, N_{FOD} = 0.355283

UKS, N_{FOD} = 0.355283 (N^{α}_{FOD} = 0.177642) (N^{β}_{FOD} = 0.177642)

Figure S57. Fractional occupation weighted density (FOD) plot of the full molecular model of **2** (hydrogens not shown) and comparison with RKS/UKS results of dissociating H_2 at 2 Å (PBE0/def2-TZVP, T=10000K results, isosurface at ±0.005 a_0^{-3}).

Different metrics for the multireference and biradicaloid character from multireference and broken-symmetry wave functions were evaluated for the minimal (Table S4) and the full molecular model (Table S5) of **2**. The *M* metric,[34] based on frontier natural orbital populations, clearly indicates a pronounced multireference character for both systems, with a value similar to that obtained for the prototypical ozone case. Equivalent results are obtained with the n_{rad} metric, which is based on the ⟨S^2⟩ expectation value for broken-symmetry Kohn-Sham wave functions or the double-excitation CI coefficient in case of CASSCF-based results.[35] The

biradicaloid character of the order of 30% obtained from the UKS wave functions here indicates a rather strong coupling of the two electrons involved in the Co–Ge bond, far below typical values exceeding 90% for authentic biradical(oid)s: e.g., we compute 86-100% biradical character for prototypical borylene-N_2/N_4 complexes published recently[36] (92-94% based on the leading CI double-excitation coefficient obtained from a CASSCF/DLPNO-NEVPT2 wave function of the same systems, compounds 3-Dur, 4-Dur and 4-Tip in ref. [36]). Consistently, the two magnetic orbitals representing the Co–Ge σ-bond obtained from UPBE0 calculations exhibit a reasonably large overlap (Figure S52).[37] We thus attribute the spin polarisation observed along this bond in UKS calculations solely to the recovery of strong non-dynamic electron correlation effects and not to a biradicaloid nature of **2** – the excess spin-density along this bond is merely an unphysical, technical artifact. Another measure of static correlation from DFT is the fractional occupation-number weighted electron density (FOD). The FOD plot for the full molecular model shown in Figure S53 clearly shows the density of 'hot' electrons[38] along the Co–Ge bond with minor contributions involving the germanium bound nitrogen. This picture consistently reflects our choice of the CASSCF(5,5) active space involving the Co–Ge bond and the N–Ge–Co π-system.

Evaluation of methods for QTAIM and ELF analysis

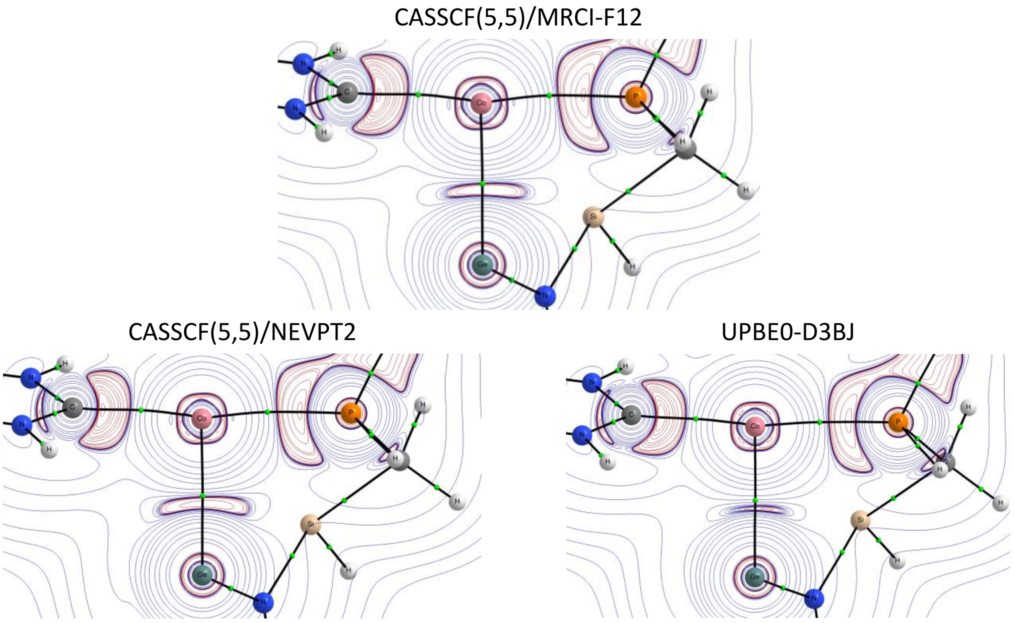

Figure S58. Comparison of the 2D plots of the Laplacian in the Ge–Co–P plane of the minimal molecular model of **2** obtained from CASSCF(5,5)/MRCI-F12, CASSCF(5,5)/NEVPT2 and BS-UKS wave functions, bond critical points in green.

Table S6. Selected properties of the Co–Ge bond critical point in the minimal model system of **2**, obtained from MRCI-F12, NEVPT2 and UPBE0 ($\langle S^2 \rangle = 1.25$) wave functions.

Method	$\rho(r_{bcp})$[a] (e Å^{-3})	$\nabla^2\rho(r_{bcp})$[b] (e Å^{-5})	$H(r_{bcp})/\rho(r_{bcp})$[c] (Hartree/e)	$G(r_{bcp})/\rho(r_{bcp})$[d] (Hartree/e)	ε[e]	δ(Ge,Co) [f]
**MRCI-F12**	0.51	–0.03	–0.45	0.44	0.21	0.78
**NEVPT2**	0.51	0.27	–0.43	0.47	0.21	0.75
**UPBE0-D3**	0.48	0.82	–0.40	0.52	0.06	0.91

[a] Electron density, [b] Laplacian of ρ, [c] relative total energy density, [d] relative kinetic energy density, [e] ellipticity, [f] delocalization index.

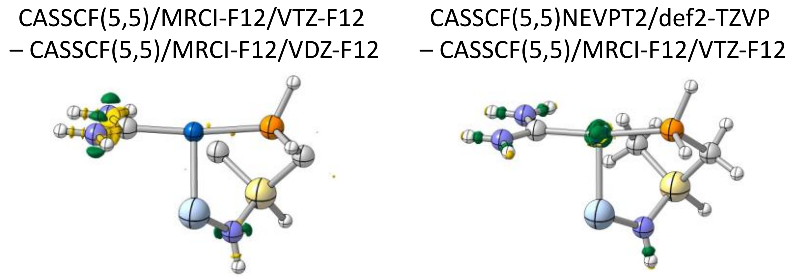

Figure S59. Density difference plots of the minimal molecular model of the minimal molecular model of **2**, (40 core orbitals frozen in the treatment of dynamic correlation; isosurfaces at ±0.005 a_0^{-3}).

The density difference plots in Fig. S55 further highlight the suitability of natural orbitals from partly contracted NEVPT2 calculations. The MRCI-F12/VTZ reference is converged with respect to the basis set expansion as only minor density changes remain going from VDZ-F12 to VTZ-F12 (top left). The $\rho^{NEVPT2}–\rho^{MRCI-F12}$ plot shows only minor differences localized on the cobalt center.

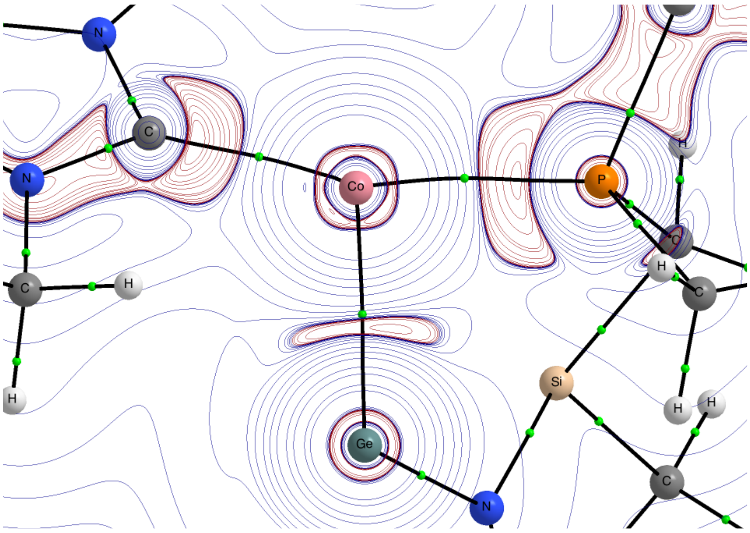

Figure S60. 2D plot of the Laplacian in the Ge–Co–P plane of the medium molecular model of **2**, bond critical points in green (NEVPT2 results).

Table S7. Characteristic properties of the electron density at selected bond critical points along the bond graph of the medium molecular model system of **2** (NEVPT2 results).

Bond	$\rho(r_{bcp})$[a] (e Å^{-3})	$\nabla^2\rho(r_{bcp})$[b] (e Å^{-5})	$H(r_{bcp})/\rho(r_{bcp})$[c] (Hartree/e)	$G(r_{bcp})/\rho(r_{bcp})$[d] (Hartree/e)	ε[e]	δ(A,B) [f]
Co–Ge	0.51	0.46	–0.44	0.50	0.24	0.79
C^{NHC}–Co	0.73	9.29	–0.39	1.29	0.43	0.55
P–Co	0.60	5.10	–0.39	0.99	0.07	0.64
Ge–N	0.92	12.20	–0.43	1.36	0.14	0.78

[a] Electron density, [b] Laplacian of ρ, [c] relative total energy density, [d] relative kinetic energy density, [e] ellipticity, [f] delocalization index.

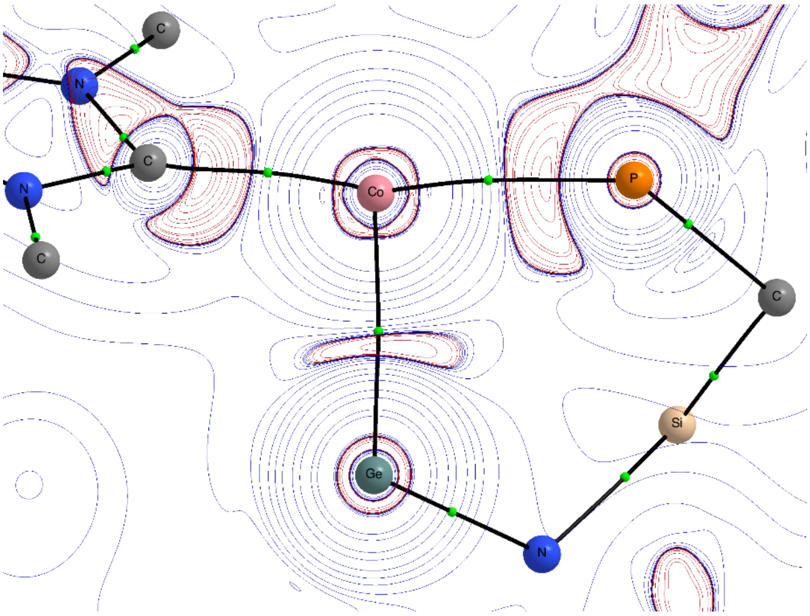

Figure S61. 2D plot of the Laplacian in the Ge–Co–P plane of the full molecular model of **2**, additional substituents omitted for clarity (NEVPT2 results).

Table S8. Characteristic properties of the electron density at selected bond critical points along the bond graph of the full molecular model of **2** (NEVPT2 results).

Bond	$\rho(r_{bcp})$[a] (e Å^{-3})	$\nabla^2\rho(r_{bcp})$[b] (e Å^{-5})	$H(r_{bcp})/\rho(r_{bcp})$[c] (Hartree/e)	$G(r_{bcp})/\rho(r_{bcp})$[d] (Hartree/e)	ε[e]	δ(A,B) [f]
Co–Ge	0.50	0.36	–0.42	0.47	0.24	0.75
C^{NHC}–Co	0.73	9.57	–0.39	1.30	0.23	0.56
P–Co	0.57	5.37	–0.37	1.03	0.19	0.61
Ge–N	0.88	11.43	–0.41	1.32	0.12	0.75

[a] Electron density, [b] Laplacian of ρ, [c] relative total energy density, [d] relative kinetic energy density, [e] ellipticity, [f] delocalization index.

Table S9. Selected ELF properties of the Co–Ge bond in the minimal molecular model of **2**, obtained from MRCI-F12, NEVPT2 and UPBE0 ($\langle S^2\rangle = 1.25$) wave functions.

Method	Bond	N(Ω)[a]	σ^{2}[b]
MRCI-F12	Co–Ge[c]	1.59 (Co: 0.66, Ge: 0.91)	1.15

	Co–P	2.16 (Co: 0.36, P: 1.80)	1.00
	Co–C^{NHC}	2.25 (Co: 0.28, C: 1.95)	0.98
NEVPT2	Co–Ge[c]	1.60 (Co: 0.62, Ge: 0.96)	1.14
	Co–P	2.17 (Co: 0.36, P: 1.81)	0.98
	Co–C^{NHC}	2.24 (Co: 0.27, C: 1.95)	0.95
UPBE0	Co–Ge[c]	1.53 (Co: 0.61, Ge: 0.91)	1.25
	Co–P	2.28 (Co: 0.47, P: 1.81)	1.22
	Co–C^{NHC}	2.35 (Co: 0.36, C: 1.96)	1.19

[a] Population of the corresponding disynaptic ELF-Basin, [b] variance of the ELF-Basin population, [c] ELF populations and variances were summed up for both basins.

Table S10. Selected ELF properties of the medium molecular model of **2**.

Method	Bond	N(Ω)[a]	$\sigma^{2[b]}$
NEVPT2	Co–Ge[c]	1.67 (Co: 0.62, Ge: 1.04)	1.17
	Co–P	2.21 (Co: 0.42, P: 1.79)	1.02
	Co–C^{NHC}	2.43 (Co: 0.26, C: 2.15)	1.02

[a] Population of the corresponding disynaptic ELF-Basin, [b] variance of the ELF-Basin population, [c] ELF populations and variances were summed up for both basins.

Table S11. Selected ELF properties of the full molecular model of **2**.

Method	Bond	N(Ω)[a]	$\sigma^{2[b]}$
NEVPT2	Co–Ge[c]	1.67 (Co: 0.62, Ge: 1.03)	1.20
	Co–P	2.15 (Co: 0.35, P: 1.79)	1.01
	Co–C^{NHC}	2.40 (Co: 0.25, C: 2.13)	1.05

[a] Population of the corresponding disynaptic ELF-Basin, [b] variance of the ELF-Basin population, [c] ELF populations and variances were summed up for both basins.

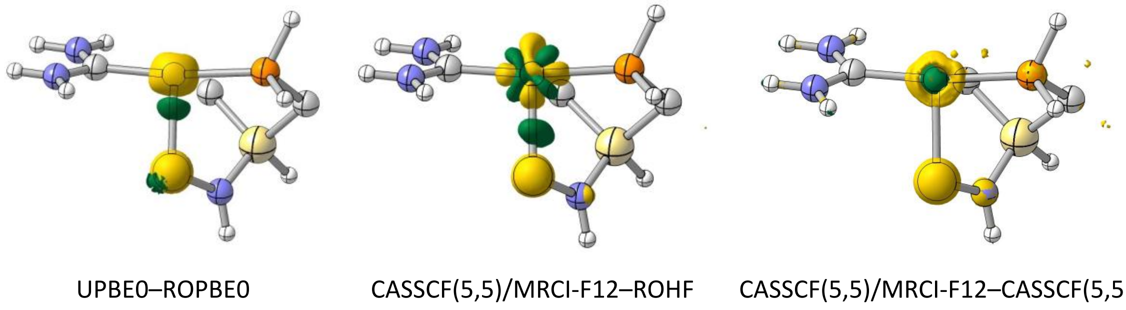

Figure S62. Density difference plots of unrestricted (⟨S^2⟩ = 1.25) and restricted open-shell calculations on the minimal molecular model of **2** (negative in green, positive in yellow).

As detailed above, the broken symmetry approach to DFT is a means to capture non-dynamic correlation effects in unrestricted methods based on a single-determinantal wave function, such as approximate Kohn-Sham DFT. Figure S58 documents the overall similar character of the UKS–ROKS and MRCI–ROHF density difference plots, which depict the effect of non-

dynamic correlation: In both cases, electron density from the Co–Ge interatomic region is transferred onto the atoms. Additional differences visible in the latter about the cobalt atom originate in dynamic correlation effects introduced in the MRCI treatment as can be seen by comparison with the MRCI-CASSCF plot where the density differences are localized on the atoms.

Table S12. NPA charges, Wiberg bond index and atomic charges q obtained from QTAIM analysis for the full molecular model of **2** and comparison with the reactants **1** and iPr–Co(η^2vtms)$_2$ (UPBE0/def2-TZVP results).

Compound	NPA (Ge)	NPA (Co)	WBI Co–Ge	q(Ge)	q(Co)
**2**	0.72	0.41	0.59	0.72	0.39
**1**	0.99	–	–	0.98	-
iPr–Co(η^2vtms)$_2$	–	0.73	–	-	0.69

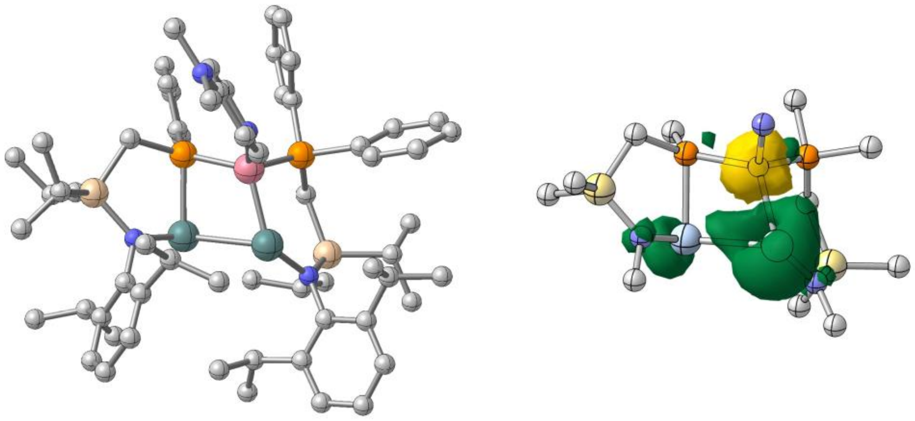

8 Spin density of **8** in the Co–Ge–Ge core
⟨S^2⟩ = 1.17

Figure S63. Optimized structure (left) and spin density (right) of compound **8**, hydrogen atoms (left) and spin-free substituents (right) not shown (broken symmetry PBE0-D3BJ/def2-TZVP//def2-SVP results, isosurface at ±0.005 a_0^{-3}).

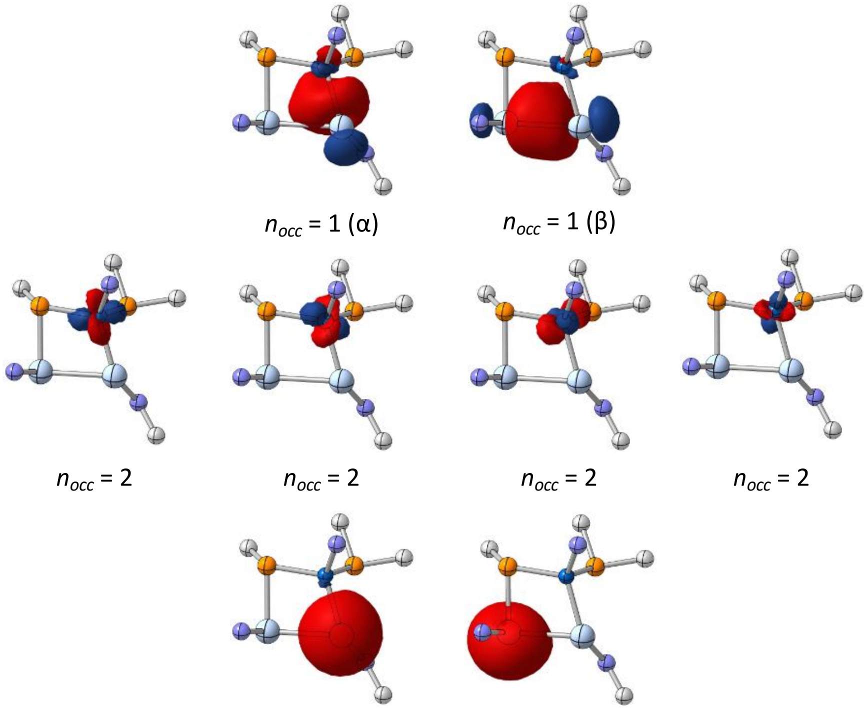

Figure S64. Top: α- and β-NLMO corresponding to bonding interactions in the Co–Ge and Ge–Ge moiety; middle: α/β averaged NLMOs corresponding to doubly occupied Co d-orbitals; bottom: α/β averaged NLMOs corresponding to Ge lone pairs (broken symmetry PBE0-D3BJ/def2-TZVP//def2-SVP results, isosurface at ±0.05 a_0^{-3}).

Due to the size of compound **8**, we used a molecular model for CASSCF/NEVPT2 calculations. During the optimization of this model the atomic positions of the metal and ligand-backbone were kept frozen. As can be seen from the spin density in Figure S61, this model features the same problem of non-dynamic correlation present in **8**.

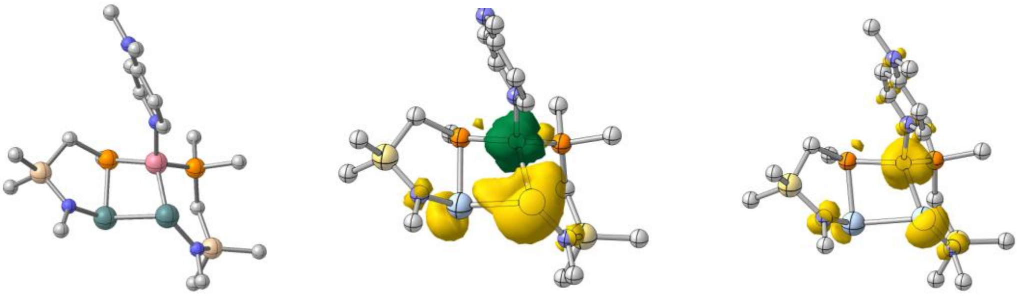

Figure S65. Optimized structure (left), spin density (middle) and FOD plot (right) of the truncated model of compound **8** used for CASSCF calculations, hydrogen atoms not shown (broken symmetry PBE0-D3BJ/def2-TZVP//def2-SVP results, FOD: PBE0/def2-TZVP, T=10000K results, isosurfaces at ±0.005 a_0^{-3}).

.

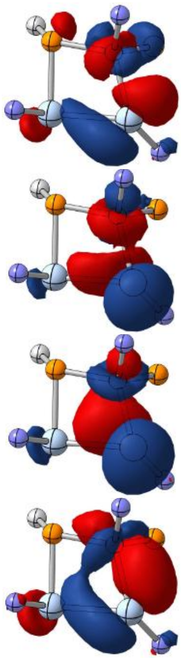

D
3.99 eV
occ. = 0.31 (0.33)

C
2.86 eV
occ. = 0.39 (0.41)

B
–4.17 eV
occ. = 1.61 (1.59

A
–4.79 eV
occ. = 1.69 (1.66)

config. [ABCD]	weight
[2200]	0.69
[2020]	0.11
[1111]	0.08
[0202]	0.04

Figure S66. CASSCF(4,4) natural orbitals with energy and occupation numbers (NEVPT2 results in parenthesis) and leading configurations for the truncated molecular model of **8** (isosurface at ±0.04 $a_0^{-3/2}$).

4. References

1. P. M. Keil, T. Szilvási, T. J. Hadlington, *Chem. Sci.* **2021**, *12*, 5582-5590.
2. P. M. Keil, S. Ezendu, A. Schulz, M. Kubisz, T. Szilvási, T. J. Hadlington, *J. Am. Chem. Soc.* **2024**, *146*, 23606–23615.
3. P. M. Keil, T. J. Hadlington, *Z. Anorg. Allg. Chem.* **2022**, *648*, e202200141.
4. J. Du, L. Wang, M. Xie, L. Deng, *Angew. Chem. Int. Ed.* **2015**, *54*, 12640-12644.
5. S. J. Bonyhady, C. Jones, S. Nembenna, A. Stasch, A. J. Edwards, G. J. McIntyre, *Chem. Eur. J.* **2010**, *16*, 938-955.
6. M. Muhr, P. Heiß, M. Schütz, R. Bühler, C. Gemel, M. H. Linden, H. B. Linden, R. A. Fischer, *Dalton Trans.* **2021**, *50*, 9031-9036.
7. Stoll, S.; Schweiger, A. EasySpin, a comprehensive software package for spectral simulation and analysis in EPR. Journal of Magnetic Resonance 2006, 178 (1), 42-55. DOI: https://doi.org/10.1016/j.jmr.2005.08.013.
8. A. Schulz, T. J. Hadlington, *Dalton Trans.* **2024**, DOI: 10.1039/D4DT02372B.
9. G. M. Sheldrick, *Acta Cryst.*, 2015, *A71*, 3-8
10. G. M. Sheldrick, *Acta Cryst.,* **2015**, C*71*, 3-8.
11. F. Neese, *Wiley Interdiscip. Rev. Comput. Mol. Sci.* **2018**, *8*, e1327.
12. C. Adamo, V. Barone, *J. Chem. Phys.* **1999**, *110*, 6158-6170.
13. F. Weigend, R. Ahlrichs, *Phys. Chem. Chem. Phys.* **2005**, *7*, 3297-3305.
14. (a) S. Grimme, S. Ehrlich, L. Goerigk, *J. Comput. Chem.* **2011**, *32*, 1456-1465; (b) S. Grimme, J. Antony, S. Ehrlich, H. Krieg, *J. Chem. Phys.* **2010**, *132*, 154104.
15. F. Neese, *J. Am. Chem. Soc.* **2006**, *128*, 10213-10222.
16. S. Grimme, A. Hansen, *Angew. Chem. Int. Ed* **2015**, *54*, 12308-12313.
17. E. D. Glendening, C. R. Landis, F. Weinhold, *J. Comput. Chem.* **2013**, *34*, 1429-1437.
18. M. J. Frisch, G. W. Trucks, H. B. Schlegel, G. E. Scuseria, M. A. Robb, J. R. Cheeseman, G. Scalmani, V. Barone, G. A. Petersson, H. Nakatsuji, X. Li, M. Caricato, A. V. Marenich, J. Bloino, B. G. Janesko, R. Gomperts, B. Mennucci, H. P. Hratchian, J. V. Ortiz, A. F. Izmaylov, J. L. Sonnenberg, Williams, F. Ding, F. Lipparini, F. Egidi, J. Goings, B. Peng, A. Petrone, T. Henderson, D. Ranasinghe, V. G. Zakrzewski, J. Gao, N. Rega, G. Zheng, W. Liang, M. Hada, M. Ehara, K. Toyota, R. Fukuda, J. Hasegawa, M. Ishida, T. Nakajima, Y. Honda, O. Kitao, H. Nakai, T. Vreven, K. Throssell, J. A. Montgomery Jr., J. E. Peralta, F. Ogliaro, M. J. Bearpark, J. J. Heyd, E. N. Brothers, K. N. Kudin, V. N. Staroverov, T. A. Keith, R. Kobayashi, J. Normand, K. Raghavachari, A. P. Rendell, J. C. Burant, S. S. Iyengar, J. Tomasi, M. Cossi, J. M. Millam, M. Klene, C. Adamo, R. Cammi, J. W. Ochterski, R. L. Martin, K. Morokuma, O. Farkas, J. B. Foresman, D. J. Fox, Wallingford, CT, **2016**.
19. (a) C. Angeli, R. Cimiraglia, S. Evangelisti, T. Leininger, J. P. Malrieu, *J. Chem. Phys.* **2001**, *114*, 10252-10264; (b) C. Angeli, R. Cimiraglia, J.-P. Malrieu, *J. Chem. Phys.* **2002**, *117*, 9138-9153.
20. W. Zou, *Molden2AIM (Version 5.0.2)*, https://github.com/zorkzou/Molden2AIM.
21. T. Shiozaki, G. Knizia, H.-J. Werner, *J. Chem. Phys.* **2011**, *134*, 034113.
22. H.-J. Werner, P. J. Knowles, G. Knizia, F. R. Manby, M. Schütz, *Wiley Interdiscip. Rev. Comput. Mol. Sci.* **2012**, *2*, 242-253.
23. K. A. Peterson, T. B. Adler, H.-J. Werner, *J. Chem. Phys.* **2008**, *128*, 084102.
24. (a) F. Weigend, *J. Comput. Chem.* **2008**, *29*, 167-175; (b) F. Weigend, A. Köhn, C. Hättig, *J. Chem. Phys.* **2002**, *116*, 3175-3183; (c) K. E. Yousaf, K. A. Peterson, *Chem. Phys. Lett.* **2009**, *476*, 303-307.
25. N. B. Balabanov, K. A. Peterson, *J. Chem. Phys.* **2005**, *123*, 064107.
26. J. G. Hill, J. A. Platts, *J. Chem. Phys.* **2008**, *128*, 044104.
27. F. Weigend, *Phys. Chem. Chem. Phys.* **2002**, *4*, 4285-4291.
28. T. A. Keith, *AIMALL (Version 17.01.25)* **2016**, Overlad Park KS, USA, http://aim.tkgristmill.com.
29. T. Lu, F. Chen, *J. Comput. Chem.* **2012**, *33*, 580-592.
30. A. Savin, R. Nesper, S. Wengert, T. F. Fässler, *Angew. Chem. Int. Ed.* **1997**, *36*, 1808-1832.
31. G. te Velde, F. M. Bickelhaupt, E. J. Baerends, C. Fonseca Guerra, S. J. A. van Gisbergen, J. G. Snijders, T. Ziegler, *J. Comput. Chem.* **2001**, *22*, 931-967.
32. E. Van Lenthe, E. J. Baerends, *J. Comput. Chem.* **2003**, *24*, 1142-1156.
33. C. Y. Legault, Université de Sherbrooke, http://www.cylview.org, **2009**.
34. G. A. Andrienko, **2015**.
